# Meteor over New York City: Brines in a primitive CM asteroid

**DOI:** 10.1126/sciadv.aea2105

**Published:** 2026-07-15

**Authors:** Peter Jenniskens, Michael E. Zolensky, Austin Gordon, Jamie Gordon, Mike Hankey, Elizabeth A. Silber, Miro Ronac Giannone, Jangmi Han, Loan Le, Marc D. Fries, Karen Ziegler, Queenie H. S. Chan, Diptimayee Behera, Jonathan S. Watson, Mark A. Sephton, James Brakeley, Bianka Munday, Yoko Kebukawa, Zack Gainsforth, Masanori Suzuki, Gregory A. Brennecka, Jan H. Render, Henner Busemann, Daniela Krietsch, Colin Maden, Kees C. Welten, Kunihiko Nishiizumi, Marc W. Caffee, Takahiro Hiroi, Stefan Ruchti, Philippe Schmitt-Kopplin, Jasmine Hertzog, Vincent Carré, Daniel P. Glavin, Jason P. Dworkin, Hannah L. McLain, Angel Mojarro, José Aponte, Denise Buckner, Nanako O. Ogawa, Yoshinori Takano, Naohiko Ohkouchi, Sonia M. Tikoo, Ji-In Jung, Eva M. Riveros, Jon M. Friedrich, Denton S. Ebel

**Affiliations:** ^1^ SETI Institute, Mountain View, CA 94043, USA.; ^2^ NASA Ames Research Center, Moffett Field, CA 94035, USA.; ^3^ ARES, NASA Johnson Space Center, Houston, TX 77058, USA.; ^4^ Independent researcher, Hillsborough, NJ 08844, USA.; ^5^ AllSky7 Network, American Meteor Society, Monkton, MD 21111, USA.; ^6^ Sandia National Laboratories, Albuquerque, NM 87123, USA.; ^7^ Amentum, NASA Johnson Space Center, Houston, TX 77058, USA.; ^8^ University of New Mexico, Albuquerque, NM 87131, USA.; ^9^ Centre for Dynamic Earth and the Solar System (CeDESS), Department of Earth Sciences, Royal Holloway University of London, Surrey TW20 0EX, UK.; ^10^ Department of Earth Science and Engineering, Imperial College London, London SW7 2BX, UK.; ^11^ Earth and Planetary Sciences, Institute of Science Tokyo, Tokyo 152-8551, Japan.; ^12^ Space Sciences Laboratory, University of California, Berkeley, Berkeley, CA 94720, USA.; ^13^ Department of Chemistry and Life Science, Yokohama National University, Yokohama 240-8501, Japan.; ^14^ Citycom Co. Ltd., Tokyo 160-0023, Japan.; ^15^ Lawrence Livermore National Laboratory, Livermore, CA 94550, USA.; ^16^ Institute of Geochemistry and Petrology, ETH Zürich, 8092 Zürich, Switzerland.; ^17^ Department of Physics and Astronomy, Purdue University, West Lafayette, IN 47907, USA.; ^18^ Department of Earth, Environmental and Planetary Sciences, Brown University, Providence, RI 02912, USA.; ^19^ Research Unit Analytical BioGeoChemistry, Helmholtz Zentrum Munich, 85764 Neuherberg, Germany.; ^20^ Analytical Food Chemistry, Technical University of Munich, 85354 Freising, Germany.; ^21^ Center for Astrochemical Studies, Max Planck Institute for Extraterrestrial Physics, 85748 Garching, Germany.; ^22^ LCP-A2MC, Université de Lorraine, Metz 57000, France.; ^23^ Solar System Exploration Division, NASA Goddard Space Flight Center, Greenbelt, MD 20771, USA.; ^24^ Center for Research and Exploration in Space Science and Technology (CRESST) and Department of Physics, The Catholic University of America, Washington, D.C. 20064, USA.; ^25^ Center for Space Science and Technology (CSST), University of Maryland, Baltimore County, Baltimore, MD 21250, USA.; ^26^ Oak Ridge Associated Universities, Oak Ridge, TN 37830, USA.; ^27^ Center for Research and Exploration in Space Science and Technology (CRESST) II, NASA Goddard Space Flight Center, Greenbelt, MD 20771, USA.; ^28^ Biogeochemistry Research Center (BGC), Japan Agency for Marine-Earth Science and Technology (JAMSTEC), Yokosuka, Kanagawa 237-0061, Japan.; ^29^ Department of Geophysics, Stanford University, Palo Alto, CA 94305, USA.; ^30^ Department of Chemistry and Biochemistry, Fordham University, New York, NY 10458, USA.; ^31^ Department of Earth and Planetary Sciences, American Museum for Natural History, New York, NY 10024, USA.; ^32^ Lamont-Doherty Earth Observatory, Columbia University, Palisades, NY 10065, USA.; ^33^ Department of Earth and Environmental Sciences, Graduate Center of the City University of New York, New York, NY 10065, USA.

## Abstract

The CI (Ivuna-type) carbonaceous material returned from asteroids Ryugu and Bennu contain mobilized sodium from the evaporation or freezing of liquid water into brines, shedding light on the internal structure of ice-rich CI-type worlds and the formation of prebiotic organic compounds. The formation of brines has not been demonstrated in CM (Mighei-type) carbonaceous chondrites, which also supplied organic matter to the early Earth. Here, we announce the fall of a primitive meteorite from a daytime fireball over the New York metropolitan area in July 2024. It is a CM2 breccia that contains unique CM1 clasts rich in water and sodium. The meteorite contains abundant amino acids and other products of organic chemistry in brines that reveal subsurface processes on CM-type asteroid parent bodies.

## Teaser

A sonic boom that shook New York City announced the fall of a meteorite that preserved the briny subsurface of an alien world.

## INTRODUCTION

The large primitive asteroid Ceres has craters with bright white areas that are dominated by sodium carbonates, thought to be evaporation deposits from sublimated briny fluids originating from an icy subsurface layer (*1*–*4*). Did other, smaller, primitive asteroid parent bodies have similar icy subsurface layers? These brines can enable organic chemistry for the origin of life by allowing phosphate to remain in solution and catalyze chemical reactions between organics and precipitate minerals (*5*–*7*).

An evaporite sequence from late-stage brine flowing through primitive asteroids has now been detected in Ivuna-type carbonaceous chondrites (CI) Ryugu and Bennu samples (*6*, *8*). Similar processes are expected to occur in other carbonaceous chondrite types, but terrestrial weathering would obscure evidence of salt precipitation (*5*).

So far, Mighei-type (CM) carbonaceous chondrites are not known to contain indigenous evaporites. They are the most common water-rich primitive meteorites to be recovered on Earth. They also experienced past aqueous alteration that created abundant phyllosilicates (*9*) but to a lesser degree than CI chondrites. Evaporates are most likely in petrographic type CM1 carbonaceous chondrites, which are more heavily aqueously altered than type CM2, but all CM1 meteorites known to date are finds, prone to terrestrial weathering (*9*, *10*).

## RESULTS AND DISCUSSION

### Impact and recovery of Hillsborough

**Fig. 1.**
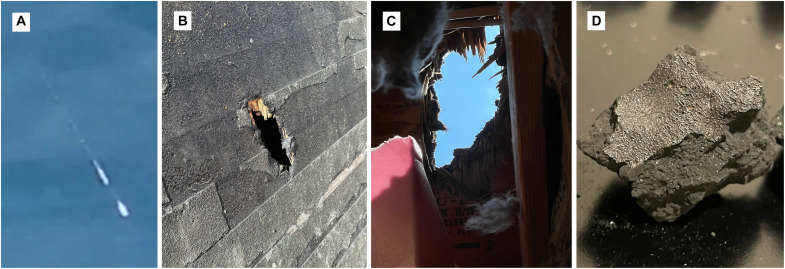
Impact and recovery of Hillsborough. (**A**) The daytime meteor with wake in an enhanced single video frame from Northford, CT. (**B**) The impact site in Hillsborough (roof tile width, 14 cm). (**C**) View of the impact site from inside. (**D**) Meteorite fragment with fusion crust. Photo credits: (A) M. Kirschner and M. Hankey, American Meteor Society; [(B) to (D)] A. Gordon.

On 16 July 2024, at 15:17:27.6 UTC, a daytime fireball with sonic boom was reported to the American Meteor Society by 60 visual observers in the states of New York, New Jersey, Connecticut, Rhode Island, and Pennsylvania, confirming media reports of a meteor over the New York metropolitan area. The meteor was filmed by two stations of the Allsky7 camera network (Fig. 1A), as well as by a doorbell camera in Wayne, NJ. Triangulation of those records show that the meteor moved in a 256° direction (East to West) over Staten Island toward New Jersey, from 44.4- to 34.9-km altitude at an angle of 29° to the horizontal (Supplementary Materials and Methods and table S1). Each flare left a wake of dust. A surviving mass caused a final flare at 28.2 km. From 2 to 16 min later, the Newark Airport Doppler weather radar detected falling meteorites of 10 to 0.1 g, respectively (figs. S1 and S2), but none of these were found.

The sonic boom was not detected by conventional infrasound arrays but recorded by doorbell video at locations near the trajectory. Two sharp overpressure maxima were identified as the leading and trailing shock signatures of the N-wave (*11*) with a peak-to-peak spacing less than the full period of the pulse. The dominant acoustic period was 0.5 ± 0.1 s. Using the recently refined empirical bolide energy relation (*12*), the kinetic energy deposited by the fireball was 1.31 ± 0.09 tons TNT equivalent. With the triangulation-derived initial speed of 14.4 ± 0.6 km/s (table S1), this implies a preatmospheric mass of 53 ± 6 kg.

Shortly thereafter, a large meteorite hit the roof of a house in Hillsborough, NJ (Fig. 1, B and C). The owner of the home heard a loud crash at approximately 15:20 UTC and found a hole in the ceiling of the master bedroom accompanied by black matter that covered the bed, carpet, and surrounding areas. The meteorite had broken into many fragments (Fig. 1D), including dust, and a strong sulfur-like odor was present in the air. Immediate care was taken to preserve and document the entire scene using disposable gloves and aluminum foil, with fragments placed in glass jars. The total recovered mass was ~1.35 kg.

### Petrography and evidence for brine mobility

Hillsborough is the 22nd observed CM-type meteorite fall, but only the 2nd fall classified as CM1/2, following the fall of Kolang in Indonesia in 2020 (*10*). This CM is unique in having escaped rain and soil and having been taken out of the terrestrial environment quickly.

**Fig. 2.**
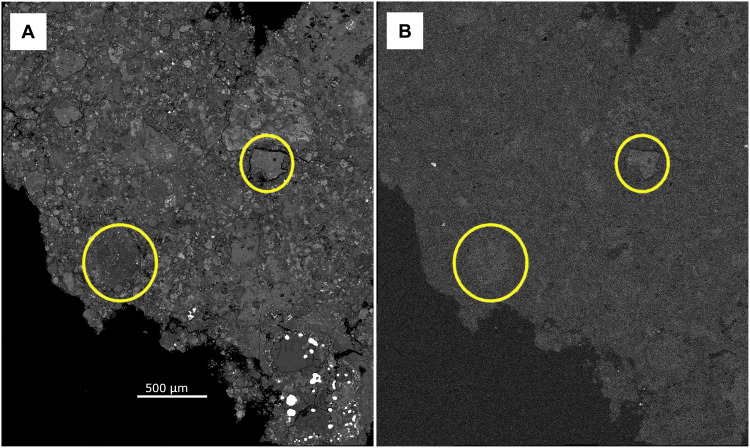
C1 clasts in Hillsborough. (**A**) Back-scattered electron image with two C1 clasts circled. (**B**) Na x-ray map of the same area as (A), indicating Na enrichment of the C1 clasts relative to the bulk of Hillsborough.

The stones were extremely friable, and no fluids could be used for cutting or polishing. Scanning electron microscopy analysis shows that the meteorite is unusually finely brecciated for a CM (Fig. 2). Some stones consist entirely of finely comminuted, submillimeter-sized fragments (fig. S3). Most of these clasts (95 to 98%) are of CM2 type, the largest continuous clast 5 mm in size.

A notable feature in Fig. 2A is the presence of a few small (<0.5 mm), matrix-dominated clasts of C1 material. Figure 2B shows how these clasts stand out in the Na element map as their bulk matrix contains, locally, in excess of 5 wt % Na_2_O (see table S2), which is significantly enriched over the normal 0.09 to 0.36 wt % in CM chondrites (*13*).

**Fig. 3.**
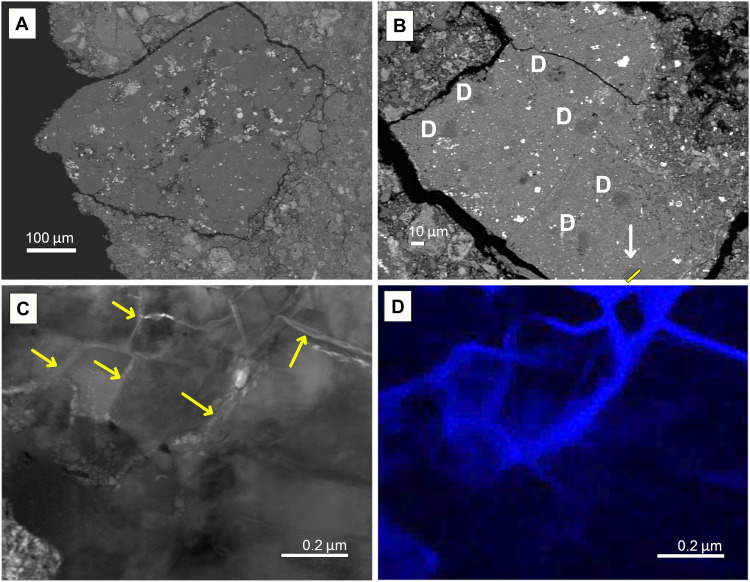
Na enriched veins in C1 clasts shown in Fig. 2. (**A**) C1 clast containing numerous magnetite (white), pyrrhotite (light gray), and dolomite grains (dark gray). (**B**) C1 clast with numerous dolomites marked “D,” indicating the dolomite crystal where the FIB was cut (bottom, arrowed, and outlined in yellow). (**C**) Bright-field TEM image of the FIBed dolomite crystal. Numerous cracks are arrowed. (**D**) Na x-ray map of the same area as in (C).

Scattered within the fine-grained matrix are crystals of dolomite and magnetite (plaquettes and framboids). Even the C1 clast dolomites (labeled “D” in Fig. 3B) have high Na, which is unusual. Transmission electron microscopy (TEM) analysis of a focused ion beam (FIB) section from a typical dolomite found that Na was concentrated within cracks (Fig. 3, C and D), as an unidentified amorphous phase inconsistent with contamination from handling or weathering since the recovery. TEM examination of a second dolomite revealed the same phenomenon.

The bulk of the carbonates in the meteorite is calcite, determined by Raman and electron probe microanalysis (EPMA). Fine-grained pyrrhotite (Fe_1-x_S) is abundant. For the most part, matrix serpentine is fully hydrated (based on low EPMA total below 90 wt %; see table S2). Hillsborough CM2 serpentine compositions span the entire range observed for CM2 chondrites, while C1 serpentine compositions plot at the iron poor edge of this range (fig. S4).

X-ray computed tomography of a 0.99-g fragment (fig. S5) resulted in a density of 1.89 ± 0.01 g/cm^3^, at the lower end for CM chondrites (*14*). This density would suggest a preatmospheric meteoroid diameter of ~38 cm. Using the above bulk density and an average CM chondrite grain density (*14*) yields a porosity of 35%, which is high for a CM chondrite. This fragment contained ~7% apparently unbrecciated clasts, primarily subequant in shape (figs. S6 and S7).

### Geochemistry and bulk properties

Further analyses were performed on bulk material to confirm the CM identification and understand the bulk CM2 host material. The oxygen isotopes of six fragments were analyzed by laser fluorination. Sample weights were between 3.8 and 7.4 mg. The results (fig. S8) all lie within the CM carbonaceous chondrite field (*15*). The average values are δ^18^O = 11.27 ± 1.60 per mil (‰), δ^17^O = 2.97 ± 0.98‰, and Δ^17^O = −2.98 ± 0.19‰ (2σ errors). Titanium isotopic compositions (11.3 mg piece) yielded ε^46^Ti = 0.59 ± 0.04, ε^48^Ti = −0.02 ± 0.04, and ε^50^Ti = 3.33 ± 0.08 (normalized to ^49^Ti/^47^Ti = 0.749766, where ε represents parts per 10,000 deviation from a terrestrial standard, with 2σ errors), plotting among other CM chondrites (fig. S9) (*16*).

The optical, near-infrared (NIR), and mid-IR reflectance spectra were measured in the range 0.3 to 40 μm (fig. S10) and show the typical bands of CM2 chondrites (fig. S11) (*17*). There is a strong OH band at 2.7 to 3.5 μm with a sharp low-wavelength band edge and a weak 0.7-μm band from charge transfer by iron in phyllosilicates.

The low ^10^Be and ^26^Al contents of ~2.6 and ~8.5 disintegrations per minute (dpm)/kg in Hillsborough measured by accelerator mass spectrometry (MS) (table S5) suggest a cosmic ray exposure (CRE) age of ~0.2 Ma, overlapping with one of the main CM-chondrite peaks (*18*, *19*).

Hillsborough is a regolith breccia that likely experienced a comparatively long exposure to solar wind (SW) at its parent body’s surface: The concentrations of trapped ^4^He and, particularly, ^20^Ne (tables S6A) are among the highest observed in CM chondrites [(*19*) and references therein]. The ^3^He/^4^He and ^20^Ne/^22^Ne ratios (table S6A) almost reach the ratios measured in pure SW returned by the Genesis mission (*20*).

Hillsborough’s exposure as a 53-kg object in the past ~0.2 Ma ago was preceded by exposure within the regolith of a larger object. The nominal ^21^Ne-derived CRE age is 2.2 to 5.7 Ma, longer than the CRE age derived from ^10^Be and ^26^Al. The regolith residence time, where Hillsborough was exposed to galactic cosmic rays (active within the first few meters) and perhaps solar cosmic rays (active within the upper few centimeters of a rock) cannot be determined without additional information regarding its shielding.

A meteorite fragment protected from magnets showed a low natural remanent magnetization of 1.27 × 10^−5^ Am^2^/kg (fig. S12). Hillsborough may have experienced aqueous alteration and formation of secondary magnetic minerals, such as magnetite and pyrrhotite, in a weak paleofield with an intensity <~400 nT, or it experienced a loss of primary magnetic mineral orientations during brecciation (*21*).

### Organic matter and prebiotic compounds

Bulk Hillsborough contains organic matter with a total carbon weight percentage of 1.76 ± 0.05 wt %, nitrogen 0.074 ± 0.003 wt %, and sulfur 6.13 ± 0.22 wt %, respectively (table S7B). The carbon and nitrogen contents are only slightly lower than the typical CM values of 2.2 and 0.10 wt %, respectively (*19*), and sulfur is in line with other CM (fig. S14). The isotopic signatures are δ^13^C [‰ versus Vienna Pee Dee belemnite (VPDB)] = −0.2 ± 0.4, δ^15^N (‰ versus air) = +28.5 ± 1.1, and δ^34^S [‰ versus Vienna Cañon Diablo Troilite (VCDT)] = −1.0 ± 0.6, respectively. Those values, too, fall among other fresh CM chondrites (*22*).

Fourier transform IR (FTIR) analyzed transmission spectra on individual grains (fig. S15) look like those of typical CM carbonaceous chondrites. The aliphatic C-H bands at 2960 and 2930 cm^−1^ are weak. The aromatic versus aliphatic nature of the macromolecular organic material, some found in nanoglobules (fig. S17), is revealed by carbon x-ray absorption near-edge structure (C-XANES) and N-XANES (fig. S16). The results fall in line with other CMs (*23*).

**Fig. 4.**
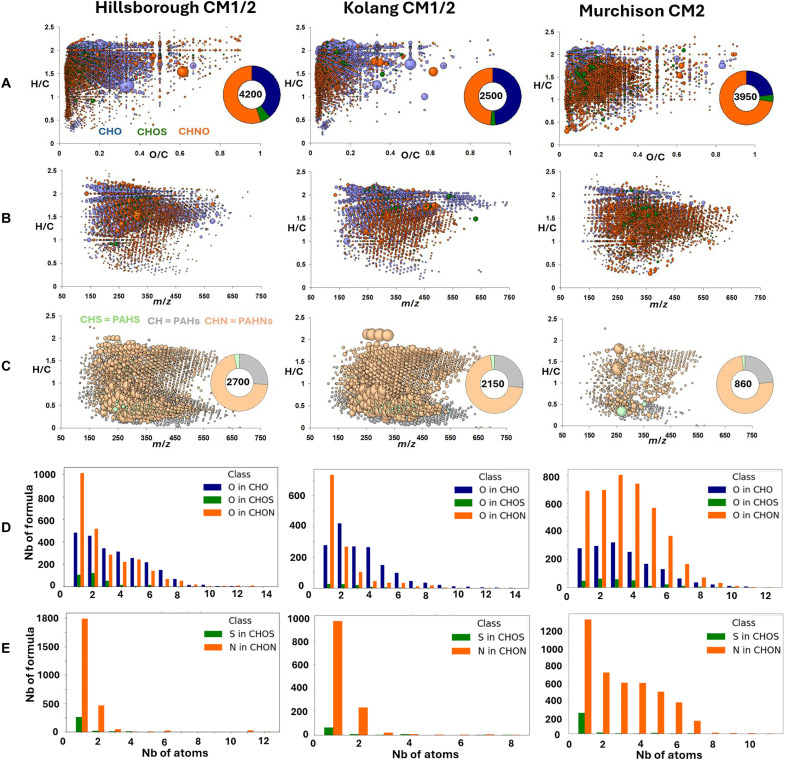
Diversity of organic compounds. Data visualization of the surface laser desorption analysis with LD(+)-FT-ICR MS of CM1/2 Hillsborough compared to CM1/2 Kolang and CM2 Murchison showing all oxidized CHO, CHNO, CHOS, and nonoxidized CH, CHN, and CHS chemical families with (**A**) classical van Krevelen diagrams; (**B**) and (**C**) mass derived van Krevelen; (**D**) distribution of the chemical formula as a function of their abundance in oxygen; and (**E**) distribution of the formulas as a function of their abundance in nitrogen and sulfur. The bubble size is proportional to the intensity in the mass spectra, while the pie charts give the number of compounds.

Laser desorption ionization FT-ICR (ion cyclotron resonance) MS in positive mode (*24*) of fresh surface (Fig. 4, A to C) presented oxygenated compounds (CHO, CHNO, and CHOS) that were more abundant than nonoxygenated heterocyclic aromatic compounds (HACs) composed of nitrogen containing CHN or sulfur containing CHS as well as alkylated polyaromatic hydrocarbons (PAHs; CH). The ratio of these oxygenated to nonoxygenated HACs and PAHs is reduced significantly from 6.1 to 1.9 and 1.3 for Murchison (least aqueously altered), Hillsborough, and Kolang, respectively (figs. S18 and S19B). The data show a concurrent decrease in the number of compounds containing multiple nitrogen (Fig. 4E). Results from FT-ICR in negative mode (fig. S18) also show a deoxygenation from Murchison to Hillsborough (fig. S19), leaving among the methanol soluble compounds in Hillsborough mostly those with low aromaticity index (fig. S20). Organomagnesium molecules can rapidly hydrolyze in water-rich environments (*25*) but the processes that caused the deoxygenation in Hillsborough affected these compounds less (fig. S19B).

PAHs and HACs in carbonaceous chondrites may either derive from the interstellar medium and/or result from secondary parent body processes, reflecting conditions of aqueous alteration (*26*, *27*). Assuming that all organic matter started out in similar form, the trends suggest the formation of reduced carbon through molecular deoxygenation in a coevolution of mineral oxidation in brines (Fig. 4D). More aqueous alteration of minerals resulted in more reduced organic compounds.

Analysis of hot water extracts of the Hillsborough meteorite (samples H02 and H03) using liquid chromatography (LC)–gas chromatography MS (GC-MS) revealed a complex mixture of C2 up to C11 unfunctionalized aliphatic primary-amine amino acids [H_2_N(CH_2_)_n_COOH], with an overall distribution similar to that observed in Murchison analyzed in parallel (fig. S21) but unlike the much lower abundance of C > 5 observed in highly aqueously altered CI and CM1 meteorites (*28*). The C6 and higher amino acids are not well separated because of numerous overlapping isomers. The total abundance of identified C2 to C5 amino acids spans a wide range (~44 to 625 nmol/g), in contrast to Murchison (~121 to 167 nmol/g; tables S9 and S15), reflecting a larger range of alteration among clasts in the Hillsborough breccia.

The terrestrially rare nonprotein amino acids including α-aminoisobutyric acid and racemic isovaline (D/L ~1; tables S9 to S11, S15, and S16) in the Hillsborough meteorite water extract are likely extraterrestrial in origin. The higher relative abundance of glycine and other alpha-amino acids compared to beta–, gamma–, and delta–amino acid isomers suggests a formation by HCN polymerization and/or Strecker-cyanohydrin synthesis (*29*) inside Hillsborough’s parent body during an early aqueous alteration phase at moderate temperatures. The dominance of α-amino acids is in contrast to their comparatively low abundance in highly aqueously altered CI1 and CM1 meteorites (*30*).

Carboxylic acids in Hillsborough H03 are predominantly ^13^C enhanced and thus likely extraterrestrial in origin (table S13). Oxalic acid in Hillsborough is less abundant and has lower ^13^C compared to Murchison, potentially reflecting differences in isotopic carbon reservoirs (*29*).

The Hillsborough meteorite did not escape some contamination from the fall and subsequent handling, resulting in high ε-amino-*n*-caproic acid abundances from contamination with Nylon-6 (commonly found in carpet and building materials) and biologically common protein amino acids with an l-enantiomeric excess (D/L ~0.05 to 0.32; tables S10 and S16). Acetic acid is ^13^C depleted in both Hillsborough and Murchison, which could point to terrestrial contamination (table S13). Pyrolysis GC-MS showed siloxanes (fig. S24) that are likely contaminants due to the meteorite falling through a fiberglass isolated roof. The distributions of thiophenes and PAHs released from Hillsborough (figs. S24 to S27) are similar to that of Murchison (*30*).

Accounting for these terrestrial contaminants, the total abundance of likely extraterrestrial amino acids in fragments H02 and H03 was 0.56 and 3.75 times that of the CM2 Murchison, respectively. This large difference may reflect different levels of amino acid decomposition by aqueous alteration in distinct lithologies.

The complex distribution of amino acids observed in Hillsborough, similar to Murchison and other moderately altered CM2 chondrites, demonstrates that these prebiotic organic molecules were formed inside the Hillsborough parent body, likely through chemical reactions that occurred during an early aqueous alteration phase. Later brine chemistry may have added complexity in some clasts. The exogenous delivery of amino acids, carboxylic acids, and other soluble organic molecules by CM-type bodies and their fragments could have been an important source of the prebiotic organic inventory that led to the emergence of life on Earth.

### CM parent body and origin of CM chondrites

We have previously observed Na enrichments associated with dolomite in a xenolithic C1 clast in the Zag ordinary chondrite (*31*), a material associated with halite and sylvite that formed on a separate parent body (*32*). However, in the case of Hillsborough, it appears likely that its C1 clast is indigenous to the CM parent body and should be classified as CM1. This is a logical proposal as exogenous C1 material is rare or absent from other CM chondrites, whereas CM1 material is frequently present (*33*).

Since the Na is most concentrated in fractures in dolomite in the Hillsborough CM1 lithology, we speculate that the enrichment is due to the passage of Na-rich, late stage brines though the CM parent body. This is evidence of significant water mobility in CM chondrites otherwise having fairly homogeneous elemental compositions (*9*). Previously, veins of phases in CM chondrites pointed to flowing water at the centimeter scale at least, while rims showed that S, Ca, and organics were transported from adjacent matrix (*34*). In the case of the Zag C1 clast (*31*), oxygen isotope geothermometry yielded a cocrystallization temperature of less than −15°C, assuming equilibrium between magnetite grains intergrown within and around a dolomite, and, hence, the late-stage fluid was an icy brine (*35*). Rather than elevated temperature (>120°C) or a higher water/rock ratio (*9*), a prolonged exposure to liquid water following impacts may be responsible for the higher aqueous alteration of the CM1 clasts, as in other CM1s (*33*). If so, Hillsborough preserved clasts from the subsurface of its parent body.

After formation, that parent body built up a layer of regolith at the surface and then experienced an exceptionally large collision event that created a family of asteroids. About 0.2 Myr ago, one of those asteroids had already evolved from the asteroid belt into a near-Earth asteroid (NEA) orbit before it lost an ~53-kg meteoroid (*36*). We identified as many as 27 known NEA with orbits similar to Hillsborough but with no spectral information.

Asteroid families composed of CM-type materials with the characteristic 0.7-μm band are found both in the inner and outer parts of the asteroid belt (*36*). Hillsborough’s approach orbit had an inclination of *i* = 4.2° ± 2.1° and (table S1), suggesting that the parent NEA had dynamically evolved via the 𝛎_6_ resonance on the near side of the belt [at semi-major axis a ~ 2.1 astronomical unit (au)]. It is possible that this NEA originated from the Erigone family in the inner main belt (*i* = 4.7°), a collisional family from ~73-km-sized asteroid 163 Erigone, some of which have the 0.7-μm band (*37*). This family includes NASA’s Lucy mission target asteroid 52246 Donaldjohanson (*37*).

However, the observations do not exclude that the Hillsborough NEA arrived via the 3:1 resonance (*a* = 2.50 au). All four documented CM2 falls arrived along this route from a low inclined (*i* < 3°) source in the outer main belt (*36*). That source remains unidentified. If so, Hillsborough confirms that the proposed Veritas asteroid family (*38*) is too inclined to be the source (*i* = 9.1°). A locally higher level of aqueous alteration in the subsurface of CM worlds would make it more likely that a collisional family associated with ~200-km asteroid 24 Themis is the source (*36*). This family is located at a low *i* = 1.1° in the outer asteroid belt. In particular, the <10-Ma-old Beagle cluster in the Themis family is a candidate source region. Only a fraction of the Themis family asteroids has the 0.7-μm band (*39*, *40*). Some family members have hydroxyl-bearing minerals (*41*), some show cometary activity (*40*), and Themis itself has water ice at the surface (*42*). Via the 2:1 resonance, Themis family asteroids can evolve into the population of Jupiter family comets (*43*), a source of CM-type micrometeorites (*44*).

## MATERIALS AND METHODS

Daytime Allsky7 (*45*) camera video records from stations AMS149 and AMS15, with a convergence angle of 38.9°, were calibrated against star background images from the night prior using methods described in (*46*). A linear trajectory and velocity deceleration profile were calculated from a continuous record of the meteor between 44.4- and 34.9-km altitude (26 and 21 frames at 25 frames/s, respectively). In addition, video from a citizen scientist security camera in Wayne, NJ, was calibrated against a night-time star background image matched to near- and far-field foreground features as in (*47*). These data were used to evaluate the uncertainties in the Allsky7 measurements. Both the AMS149 and Wayne cameras captured an additional flare at ~28.2-km altitude.

The Hillsborough meteorite fall was recorded by weather radars in the National Oceanic and Atmospheric Administration’s *NEXRAD* network, as well as Terminal Doppler Weather Radar (TDWR) radars operated by the Federal Aviation Administration. NEXRAD uses WSR-88D radars operating in the S band (10.6-cm wavelength), while TDWR radars operate in the C band (5.3-cm wavelength).

For petrography, samples were imaged and analyzed in the E-beam laboratories of Astromaterials Research and Exploration Science (ARES) at NASA Johnson Space Center. A JEOL 7600F scanning electron microscope was used to collect images and x-ray spectra. Mineral compositional analyses were measured using a JEOL JXA 8530F electron microprobe (EPMA) at 15 kV and 20 nA. Natural minerals and alloys were used as standards. One ~100-nm-thick section was made from a dolomite crystal from one of the C1 grains using a Quanta 3D FEG Dual Beam FIB instrument. The mineralogy, chemistry, and microstructures from the FIB slice were investigated using a JEOL 2500SE scanning TEM with a JEOL Silicon Drift Detector energy dispersive x-ray spectrometer.

A 0.9946-g fragment of Hillsborough was imaged at the American Museum of Natural History’s Microscopy and Imaging Facility by computed x-ray microtomography (μCT), using a ZEISS Xradia 630 Versa μCT system with current of 125 μA and potential of 80.0 kV (10.0 W power). Data were collected at a resolution of 8.0 μm per voxel edge. Using Blob3D (*48*), the total internal volume of the sample was calculated by digitally isolating the chondrite from the air surrounding the sample. The mass was measured using a calibrated analytical balance. The porosity was calculated by assuming the mean grain density of CM chondrites (2.92 g/cm^3^). ImageJ and the TrakEM2 application (*49*) were used to visually identify and digitally isolate unbrecciated CM clasts within the sample following the same methods used to isolate individual chondrules by (*50*). Blob3D was used to calculate clast volume and axial dimensions. A best-fit ellipsoid’s principal axes was used to calculate the shape according to (*51*) using the shape categories of (*52*).

The oxygen isotopes of six fragments were analyzed at the University of New Mexico by laser fluorination (*46*). Sample weights for each measurement were between 3.8 and 7.4 mg. Each sample was compared 20 times to a standard.

Titanium was purified using a two-stage ion-exchange chromatography procedure following the methods outlined in (*53*). After dissolution, the sample solution was converted into 2-ml 12 M HNO_3_ and loaded onto DGA Resin cartridges in combination with a vacuum box from Eichrom Technologies. After loading, most elements were eluted with 12 ml of 12 M HNO_3_ before Ti was collected with 12 ml of 6 M HNO_3_. The cut containing Ti was then converted into 2 ml of 0.4 M hydrochloric acid (HCl)–1 M HF and was further purified using 2 ml of precleaned and preconditioned Bio-Rad AG-1X8 (100 to 200 mesh) anion exchange resin in Bio-Rad polyprep columns. Remaining Ca, V, and other impurities were eluted with an additional 6 ml of 0.4 M HCl–1 M HF before Ti was collected with 8 ml of 6 M HCl. The Ti cuts were dried down and converted into 2% HNO_3_–0.005 M HF for multi-collector induced coupled plasma MS (MC-ICPMS) measurements. Yields of this entire procedure were >90%, and procedural blanks are typically ~2 ng, which is negligible compared to ~7 μg of Ti that was processed for Hillsborough. To assess the accuracy of our methods, we also processed US Geological Survey rock standard BCR-2 alongside Hillsborough, for which ε^46^Ti = −0.02 ± 0.08, ε^48^Ti = 0.01 ± 0.03, and ε^50^Ti = −0.06 ± 0.10 were measured, values that are consistent with previously reported data for this standard [e.g., (*53*)].

Isotopic measurements of Ti were performed at Lawrence Livermore National Laboratory using a Thermo Fisher Scientific Neoma MC-ICPMS in combination with a Cetac Aridus II desolvating introduction system and an ~50 μl/min Savillex PFA Nebulizer. Since certain isotope masses of Ti are affected by isobaric interferences from polyatomic species (e.g., ^36^Ar^14^N), the isotope measurements were performed in medium-resolution mode on the flat low-mass shoulder plateaus of the peak to resolve and avoid these interferences. Using a Jet and X cone setup, this setup yielded ~35 V on ^48^Ti with 250 ng/g solutions. Atomic masses 43 to 53 were acquired with a single cup configuration, including all Ti isotopes, as well ^43^Ca, ^44^Ca, ^51^V, ^52^Cr, and ^53^Cr to monitor and correct isobaric interferences from ^46^Ca, ^48^Ca, ^50^V, and ^50^Cr. All signals were collected with Faraday cups connected to 10^11^-Ω amplifiers. Measurements consisted of 50 cycles of 8-s integration time. To account for instrumental mass fractionation, samples were bracketed with the OL-Ti reference standard.

Chip and powder (ground and dry-sieved to <125 and 125- to 500-μm fractions) samples of Hillsborough meteorite were loaded onto black Teflon-coated dishes of 4 mm in diameter, and their reflectance spectra were measured at the Reflectance Experiment Laboratory (RELAB). Bidirectional visible and NIR (VNIR) reflectance spectra were measured at 30° incidence and 0° emergence angles over the wavelength range of 0.3 to 2.6 μm with Spectralon as the standard. The samples and standard were spun at a speed of 1.5 rotation per second. The field of view was about 2.8 mm. Biconical FTIR reflectance spectra were measured over the wavelength range of 0.8 to 100 μm with diffuse gold as the standard using a PIKE AutoDiff accessory attached to a Thermo Fisher Scientific Nexus 870 spectrometer. The FTIR spectra were scaled and connected to the VNIR spectra at 2.5 μm to improve their reflectance accuracy. The VNIR spectra were compared with other CM chondrite spectra, and the 3-μm hydration band spectra were fitted with a continuum and Gaussians to compare the 2.7-μm band position and shape with those of many other carbonaceous chondrites (*17*, *54*).

For cosmogenic radionuclide analysis, two aliquots (of 5.10 and 50.04 mg) of the Hillsborough meteorite were dissolved in a 3:1 mixture of HF/HNO_3_ in the presence of Be (0.61 to 0.63 mg) and Cl (3.4 to 3.5 mg) carriers by heating the samples in Parr Teflon digestion bombs for 20 to 24 hours at 125° to 140°C. After cooling off the samples to room temperature we separated Cl as AgCl (for cosmogenic ^36^Cl analysis) and took a small aliquot of the dissolved sample for chemical analysis by inductively coupled plasma optical emission spectroscopy (table S5). Be and Al were separated using ion exchange chromatography and acetyl-acetone solvent extraction procedures described previously [e.g., (*55*)]. The Be and Al fractions were purified, converted to BeO and Al_2_O_3_ and mixed with Nb powder before being loaded into a stainless steel cathode. The ^10^Be/Be and ^26^Al/Al ratios were measured by accelerator MS at Purdue University (*56*).

All noble gas isotopes of He to Xe were measured in a sample of 18.247 ± 0.015 mg at ETH Zürich. The gases were released by melting the sample at ~1700°C in one step in a Mo crucible. A reextraction at slightly higher temperature demonstrated the completeness of the extraction in the main step (the reextraction contained <0.2% of the totally released ^4^He, ^20^Ne, ^36^Ar, ^84^Kr, and ^132^Xe, respectively). Blank corrections amounted to ≤0.5% of the measured concentrations for all isotopes, except for ^40^Ar (12%). Details of gas cleaning, separation, instrumentation, and component decomposition are given in (*19*, *57*, *58*).

A 0.149-g fragment was transported in a magnetically shielded case from Hillsborough to Stanford University and subjected to paleomagnetic and rock magnetic characterization. We conducted alternating field demagnetization of natural remanent magnetization. In addition, we measured anhysteretic remanent magnetization (ac field = 100 mT and dc bias fields ranging from 0.05 to 0.8 mT) and isothermal remanent magnetization acquisition (dc fields = 100 and 900 mT) (*59*). All experiments were conducted using a 2G Enterprises 755 superconducting rock magnetometer (sensitivity: 10^−12^ Am^2^) housed within an ~300-nT ambient field magnetically shielded room.

At the Biogeochemistry Research Center, stable isotope ratio MS (IRMS) was used to measure the stable isotope ratios and the relative abundances of C, N, and S (*60*). An ~10 mg of the Hillsborough sample was powdered and homogenized in an agate mortar and pestle before analysis. Approximately 1 mg of the powdered sample was analyzed using a sensitivity-modified Thermo Fisher Scientific Finnigan Flash EA1112 Elemental Analyzer coupled to a Delta Plus XP isotope ratio mass spectrometer via a ConFlo III interface (nano-EA/IRMS) (*61*), to quantify carbon and nitrogen contents and their isotope values (δ^13^C, δ^15^N). Sulfur content and δ^34^S values were analyzed using a separate nano-EA/IRMS system consisting of a modified Flash2000 Elemental Analyzer, a Shimadzu GC-2010 plus gas chromatograph, a ConFlo III interface, and a Delta Plus XP IRMS (*61*). For carbon and nitrogen analysis, 0.3 to 1 mg of sample was wrapped in a precleaned, smooth-wall tin capsule and introduced into the EA (*62*), whereas for sulfur analysis, 0.02 to 0.1 mg of sample was wrapped in a tin foil capsule. For calibration of δ^13^C and δ^15^N values, five interlaboratory-calibrated amino acid standards (*63*) were analyzed alongside the samples. Three international silver sulfide standards were used for δ^34^S calibration. Details of these standards are provided in table S7A, along with the analytical errors estimated by repeated analysis of BG-T (δ^13^C, δ^15^N) and IAEA-S-1 (δ^34^S).

To quantify the C, H, N, and S contents, about 900 mg of Hillsborough sample was powdered and homogenized in a ceramic mortar and pestle inside a laminar flow hood under high-efficiency particulate air (HEPA)–filtered positive pressure (equivalent to ISO Class 4–5). The powdered sample was stored in a N_2_-purged desiccator for 2 weeks to minimize the amount of absorbed atmospheric water that the samples may contain (mass loss was ~1.5 wt %). Approximately 11 mg of the powdered Hillsborough sample was analyzed with an Elementar vario MICRO cube. Samples were introduced directly from a sample carousel into the EA where they were combusted at 1150°C in a combustion tube containing WO_3_ and reduced at 850°C in a reduction tube containing copper granules. The N_2_, CO_2_, H_2_O, and SO_2_ gases were analyzed by a thermal conductivity detector. A total of 0.25 to 5 mg of sulphanilamide (C_6_H_8_N_2_O_2_S: C = 41.85 wt %; H = 4.68 wt %; N = 16.27 wt %; S = 18.62 wt %), purchased from Elemental Microanalysis, was used as a calibration standard, which was used to normalize and correct the data at regular intervals (i.e., every 10 samples). Acetanilide (C_8_H_9_NO: C = 71.09 wt %; H = 6.71 wt %; N = 10.36 wt % purchased from Sigma-Aldrich) and several terrestrial soil samples with various CHNS abundances (C = 3.26 to 8.98 wt %; organic C = 0.24 to 7.04 wt %; N = 0 to 0.43 wt %; S = 0.11 to 3.85 wt %) were used as in house standards to monitor the accuracy and precision of the measured elemental compositions throughout the runs (i.e., one standard every 20 samples), and the absolute SDs for the CHNS analyses determined by a triplicate analysis of the reference standard were 0.03% for C, 0.051% for H, 0.04% for N, and 0.278% for S. The C, N, and S abundances typically vary by 0.04 to 0.2 wt % from their reference values. Blanks (air) were run between every sample to reduce memory effect that may arise in the adsorption column of the EA. Each run takes about 10 min, and the entire batch of analyses was completed within a day.

FTIR micro-spectroscopy (micro-FTIR) measurements were conducted at the Institute of Science Tokyo as explained in the methods described in (*64*). Grains were pressed between two diamond windows (3.5-mm diameter and 0.3-mm thick). After separation of the two diamond windows, usually the samples are evenly distributed on both windows. IR absorption spectra were collected from each diamond window with a micro-FTIR (JASCO FT/IR-6100 + IRT-5200), equipped with a ceramic IR light source, a germanium-coated KBr beam splitter, a liquid-nitrogen cooled mercury-cadmium-telluride detector, and ×16 Cassegrain mirrors. An Ar flow heating stage (Linkam 10036L) was used to eliminate adsorbed water.

Ultrathin sections (approximately 100 nm thick) were prepared using a FIB system (SMI3200, SII NanoTechnology Inc.) at Photon Factory, High-Energy Accelerator Research Organization (KEK). C and N *K*-edge XANES analyses were performed with the scanning transmission x-ray microscope at BL19A at Photon Factory, High-Energy Accelerator Research Organization (KEK) (*65*), following similar methods shown in (*66*). Microspectral image stacks were acquired with 0.06-μm steps per pixel for 5 μm–by–5 μm to 5 μm–by–8 μm areas. C-stacks were analyzed using an autoencoder neural network with in-house software in Python (*67*).

For laser desorption ionization FT-ICR MS in positive mode, the raw meteorite samples were deposited on a stainless steel target at Helmholtz Zentrum Munich. A 355-nm wavelength laser with a spot diameter of 50-μm and 2000-Hz frequency was used. Ions generated by 100 laser shots were stored in a hexapole for 0.05 s before being transferred into the ICR cell. Laser power was adjusted for each sample to detect MS signals while limiting fragmentation and recombination phenomena. Ion source as well as instrument parameters were optimized via software ftmsControl (V2.3.0, Bruker Daltonics, Bremen, Germany). Before acquisition, the mass spectrometer was externally calibrated and the ICR detection cell was shimmed and gated using red phosphorus deposited on the matrix-assisted laser desorption/ionization plate. Mass spectra resulted from accumulation of 200 scans over a mass/charge ratio (*m*/*z*) range of 107.5 to 1500 and with an eight megaword time domain. At *m*/*z* 400, a mass resolution of 600 000 was achieved. Electrospray ionization in negative mode [ESI (−) FT-ICR MS] was performed with soluble organic matter methanol extract in a standardized way as described in (*68*).

At NASA Goddard Space Flight Center, all glassware, ceramics, and sample handling tools for amino acid analysis were rinsed with Milli-Q ultrapure water [18.2 MΩ·cm, <3 parts per billion (ppb) total organic carbon], wrapped in aluminum foil, and heated in a furnace at 500°C in air overnight. Most of the chemicals and reagents were purchased from Sigma-Aldrich. A stock amino acid solution was prepared by mixing individual amino acid standards (97 to 99% purity) in Milli-Q ultrapure water. All chiral amino acid standards were purchased as racemic mixtures (where the d amino acid concentration is equal to the l amino acid concentration) except for the d-threonine (Sigma-Aldrich), l-threonine (Sigma-Aldrich), d-isovaline (Acros Organics), and l-isovaline (Acros Organics), which were prepared as racemic mixtures by mixing the appropriate masses of each standard in Milli-Q ultrapure water to the standard mixture. Acid vapor hydrolysis used Tamapure-AA-10-HCL 20% (metallic impurity <10 pg/ml). Cation-exchange resin (AG50W-X8, 100 to 200 mesh, hydrogen from, Bio-Rad) was used for the removal of salts and interfering ions from the samples. During the desalting protocol, 1.5 N HCl, 2 M sodium hydroxide (NaOH), and 2 M NH_4_OH were used. The 2 M NaOH was prepared by dissolving 32 g of NaOH pellets (Sigma-Aldrich) in 400 ml of Milli-Q ultrapure water, the 1.5 N HCl was prepared from diluting the Tamapure HCl, and the 2 M NH_4_OH was prepared in vacuo using Milli-Q ultrapure water and ammonia gas (Air Products).

To extract the amino acid sample, the Hillsborough meteorite (81.9 mg), Murchison meteorite (82.3 mg), and fused silica FS-120 (109.8 mg), and procedural blank samples were flame-sealed in glass ampoules in 1 ml of Milli-Q ultrapure water and then heated at 100°C for 24 hours. After heating, the ampoules were opened and centrifuged at 3000 rpm for 3 min, and the water supernatants were then transferred from the ampoules by pipetting into preweighed amber glass vials. Another 1 ml of Milli-Q ultrapure water was added to each glass ampoule, the ampoules were recentrifuged, and the supernatant was transferred to the sample amber glass vials (this process was repeated one final time to maximize the recovery of the water extracts). After extraction, 45% of the supernatant was dried down under vacuum and subsequently subjected to 6 M HCl vapor hydrolysis at 150°C for 3 hours. The HCl acid–hydrolyzed and 45% of the nonacid vaporized supernatant were desalted using AG50W-X*, 100 to 200 mesh, hydrogen form, Bio-Rad cation exchange resin. Both the vapor-hydrolyzed (45%) and non–vapor hydrolyzed (45%) supernatant sample sets were eluted from the cation exchange desalting columns using 7 ml of 2 M NH_4_OH. After desalting, the eluent was dried under vacuum and brought up in 100 μl of Milli-Q ultrapure water. From this reconstituted sample, 20 μl was dried down with 20 μl of sodium borate buffer (pH 9). After drying down, the samples were brought up in 20 μl of Milli-Q ultrapure water and 5 μl of 0.1 M OPA/NAC derivatization agent and allowed to react for 15 min at room temperature before quenching with 75 μl of 0.1 M hydrazine.

Amino acid abundances, distribution, and enantiomeric ratios were determined by LC with fluorescence detection and time-of-flight MS (ToF-MS). The amino acids in the NH_4_OH eluates were derivatized with OPA/NAC for 15 min at room temperature followed by their separation and analysis using a Waters ACQUITY UPLC and Waters Xevo G2-XS Q-ToF-MS operating in positive ion mode. C2 to C6 amino acids were chromatographically resolved using a Waters BEH C18 column (2.1 mm by 50 mm, 1.7-μm bead) and a Waters BEH phenyl column (2.1 mm by 150 mm, 1.7-μm bead) in series. Both columns were maintained at 30.0°C. The mobile phase conditions for amino acid separations were as follows: flow rate, 150 μl/min; gradient, time in minutes (%B): 0 (0), 35 (55), and 45 (100). C5 amino acid isomers and enantiomers were chromatographically separated using the same chromatography conditions as for the C2 to C6 amino acids but required the implementation of a different gradient. The gradient used for C5 amino acid isomers and enantiomers was structured via time in minutes (%B): 0 (15), 25 (20), 25.06 (35), 44.5 (40), and 45 (100).

During the Waters Xevo *G2-XS* quadrupole ToF-MS analysis, a dual ESI system was used for the purpose of implementing lock mass corrections. The primary ESI source was operated using the following parameters: capillary voltage, 3.0 kV; sampling cone voltage, 40 V; source temperature, 120°C; desolvation gas (N_2_) temperature, 350°C; cone gas (N_2_) flow, 50 liter hour^−1^, desolvation gas flow rate, 750 liter hour^−1^. Because of the possibility that minor variations in the *m*/*z* scale may occur while executing experimental runs after instrument calibration is performed, a reference ESI source was implemented to supply an independent leucine enkephalin standard signal. The reference ESI source was operated using a sample infusion rate of 20 μl min^−1^, a sample fill volume of 250 μl, a lockspray infusion rate of 10 μl min^−1^, a capillary voltage of 3.0 kV, a reference cone voltage of 30 V, and a collision energy of 6.0 V. The ToF analyzer was operated in “Sensitivity mode,” which used a reflectron to provide a full width at half maximum resolution of <22,000 based on the [M + H] + of leucine enkephalin, *m*/*z* 556.2771.

For carboxylic acid analysis at NASA Goddard, all glassware and sample handling tools were rinsed with Milli-Q ultrapure water (18.2 MΩ·cm, <3 ppb total organic carbon), wrapped in aluminum foil, and combusted in a furnace at 500°C in air overnight. Solvents, standards, and reagents were purchased from Sigma-Aldrich, Thermo Fisher Scientific, and Alfa Aesar and used without purification, except 6 M HCl, which was double-distilled. Chemicals include semiconductor grade NaOH, HPLC grade dichloromethane (DCM), and *n*-propanol (99%). A stock solution of 23 carboxylic acids was prepared by mixing individual standards (97 to 99% purity) in Milli-Q ultrapure water. Two molar NaOH was prepared by dissolving 32 g of semiconductor grade NaOH pellets in 400 ml of Milli-Q ultrapure water. Functionalized aminopropyl silica gel (SiliCycle, SiliaBond, 40- to 63-μm particle size) was cleaned using methanol and DCM rinses and dried under vacuum.

A 45% portion of each non–vapor hydrolyzed hot water extract (prepared as a companion for amino acid analyses) was allocated for carboxylic acid analysis (36.5 mg of Hillsborough and 37.0 mg of Murchison). The extracts were made basic with the addition of 20 μl of 2 M NaOH, dried under reduced pressure, and derivatized with an *n*-propanol esterification protocol, modified from previously described methods (*69*). Dried residues were suspended in 20 μl of 6 M HCl, 30 μl of *n*-propanol, and 200 μl of DCM and then heated at 100°C for 16 hours in sealed PFTE-lined screw cap vials on a heating block; this reaction converts carboxylic acids into their respective *n*-propyl esters. Derivatized extracts were cooled to room temperature, passed through aminopropyl silica gel [5-mm length by 5–mm inside diameter (ID)], and the filtrate used for analysis.

Carboxylic acid concentrations and compound-specific ^13^C isotopes in samples and procedural blanks were quantified with GC-MS and isotope ratio MS (GC-MS/IRMS) with a custom setup that enables simultaneous measurements of molecular structures and their compound-specific stable isotopes from a single injection. The GC-MS/IRMS consists of a Thermo Fisher Scientific Trace GC Ultra, whose output is split: Approximately 10% is directed to a Thermo Fisher Scientific DSQ II triple-quadrupole MS through a transfer line heated to 320°C, and 90% to a Thermo Fisher Scientific MAT 253+ IRMS oxidation reactor through a Thermo Fisher Scientific GC-C III interface via a 250-μm deactivated silica column (Restek). Carboxylic acid abundances were quantified from peak areas generated using the average of three separate GC-MS measurements on the same sample and then blank-subtracted. Isotopes were quantified using ^13^C/^12^C (‰) ratios from the average of three separate IRMS measurements per sample.

The GC was equipped with two Rxi-5Sil MS (30-m length by 0.25-mm ID by 0.50-μm film thickness, Restek) and one PoraBond Q PT column (25-m length by 0.25-mm ID by 3.00-μm film thickness, two particle traps, Agilent), joined in series by SilTite μ-union connectors (Restek). Analyses used triplicate injections of derivatized carboxylic acids in aliquots of 3 μl through an injector heated at 270°C in splitless mode. The oven program began with a temperature of 50°C held for 1 min, ramped to 300°C at 5°C/min, and held at a final temperature of 300°C for 15 min. Ultrahigh purity grade helium (5.0 grade) carrier gas was delivered at a constant flow of 2.0 ml/min.

The MS used full-scan mode over *m*/*z* from 30 to 600, the filament was turned on 16 min after injection, and the ionization source was heated at 250°C in electron impact (EI) mode at 70 eV. Using Thermo Fisher Scientific Xcalibur software, carboxylic acid derivatives were identified and quantified by comparison to a mix of 23 carboxylic acid reference standards taken through the derivatization procedure and application of calibration curves, as described elsewhere (*69*).

Analytes sent to the IRMS were converted to CO_2_ in a ceramic oxidation reactor containing a copper (II) oxide/platinum/nickel wire (Thermo Fisher Scientific) heated to 940°C. Isotopic values were evaluated using high-purity CO_2_ reference gas (δ^13^C = −43.35‰ VPDB, SD = 0.08‰) that was precalibrated against commercial reference CO_2_, with 10 pulses introduced into the IRMS at the beginning of each run and 5 more at the end. IRMS data were analyzed using Thermo Fisher Scientific Isodat 2.5 software. The derivatized mix of 23 reference carboxylic acids used for quantification of GC-MS data was also analyzed by IRMS, and the same underivatized standards were analyzed on a Costech ECS 4010 combustion EA connected to the IRMS to correct for carbons added during derivatization. To calculate the δ^13^C value of each derivatized carboxylic acid, a correction was applied according to (*70*). The δ^13^C value for the carbon added through esterification is determined for each individual carboxylic acid, accounting for kinetic isotope effects during derivatization. The precision of the calculated value depends on the precision of the three measurements (i.e., derivatized sample, derivatized standard, and underivatized standard). Hillsborough, fused silica control, and coanalyzed Murchison samples were prepared by loading ~1 mg of fines into quartz pyroprobe tubes (CDS Analytical, catalog: 6201-3004) inside a HEPA-filtered laminar flow bench. All glassware used to handle samples and pyrolysis tubes were previously combusted at 550°C for ~16 hours in air.

Pyrolysis experiments were conducted on a CDS Analytical 6200 pyroprobe configured for manual loading and flash (10°C ms^−1^) heating ramps under a continuous flow (35 ml min^−1^) of ultrahigh purity (>99.9%) helium. Samples were heated in the pyrolysis furnace from 50° to 600°C (pyroprobe actual ~610°C) and held for 20 s to thermally degrade insoluble organic matter (IOM) and extract the stable hydrocarbon fraction. The pyroprobe housing and valves were held at 300°C, and volatiles were transferred via a heated transfer line (300°C) directly into a Thermo Fisher Scientific TRACE 1600 gas chromatograph coupled to a Thermo Fisher Scientific 9610 triple quadrupole MS system. The inlet temperature was held at 300°C and operated with a 10:1 split.

The GC was fitted with an Rtx-5 ms capillary column (30 m by 0.25 mm by 0.25 μm, Restek) with a 5-m Integra-Guard column, He carrier flow at 1.5 ml min^−1^, and MS transfer line set to 300°C. The GC oven was programmed with the following method: 40°C hold for 5 min, followed by a 3.5°C min^−1^ ramp to 300°C, and then a final isothermal hold at 300°C for 8.5 min (~88 min total). The MS source was held at 300°C and was operated in EI mode at 70 eV and simultaneous fullscan (*m*/*z* 50 to 500) and multiple reaction monitoring (MRM). Pyrolysis-GC–triple quadrupole MS operated in MRM mode works by using specific precursor-product reactions in which Q1 scans for a specific precursor ion, filtered ions enter a collision cell (q), and Q3 scans for its diagnostic production. MRM transitions targeted IOM-derived hydrocarbons and S-, N-, and O-containing compounds informed by prior pyrolysis experiments on CM, CR, and CI carbonaceous chondrites. Pyrolysis blanks preceded all standard pyrolysis experiments to control the cleanliness of the analytical set-up and prevent potential cross-contamination. Results were analyzed using Chromeleon 7.3.1 software. Compound identification was conducted via comparison with retention time, three MRM transitions of standards, mass fragmentation patterns of standards, and comparison to previously characterized analog materials (i.e., carbonaceous chondrites and coal standards).

For amino acid analysis at the Royal Holloway University of London, UK, a fragment of the Hillsborough meteorite (903.7 mg) and a fragment of the Murchison meteorite (368.8 mg) were separately wrapped in sterile aluminum foil and stored in a N_2_ purged desiccator before soluble organic matter extraction procedure. The fragments were powdered and homogenized in separate ceramic mortar and pestle inside a laminar flow hood under HEPA-filtered positive pressure (equivalent to ISO Class 4–5). A total of 10.8 mg from the powdered Hillsborough sample was analyzed for elemental abundances, and 757.1 mg went through a soluble organic extraction process described below. Environmental samples (total mass = 204.5 mg; including fiberglass insulation, rug floor dirt, and carpet) collected from where the meteorite fragment fell was analyzed to assess terrestrial contamination from the local environment. An empty ampoule the procedural blank of GC-MS analysis. An amino acid standard mixture was made by combining individual standard solutions (10^−3^ M) prepared from dissolution of standard amino acid crystals in ultrapure water. The amino acid standards mixture, procedural blank (empty and sterile ampoule), and environmental sample were subjected to the same experimental procedures as the meteorite sample.

All tools, glassware, and ceramics were sterilized by baking at 500°C in air for 24 hours. Millipore ultrapure water (18.2 MΩ cm, ≤3 ppb total organic carbon) was used for all laboratory work performed in this study. The amino acid 3-amino-3-methyl butanoic acid and 3-amino-2,2-dimethylpropanoic acid were provided by Astrobiology Analytical Laboratory at Goddard Space Flight Center, NASA. All other amino acid standard crystals/powder were purchased from Acros Organics, Sigma-Aldrich, and Fluka. HCl (37%), ammonium hydroxide (NH_4_OH) (28 to 30 wt %), isopropanol (IPA) (99.5%), and trifluoroacetic anhydride (TFAA) (≥99.0%) were purchased from Sigma-Aldrich. Acetyl chloride (99+ %) and pyrene (98%) were from Acros Organics. Prepacked columns, analytical grade 50 W-X8, hydrogen form (100 to 200 mesh) were acquired from Bio-Rad. NaOH pellets and DCM (99.8+ %) were bought from Thermo Fisher Scientific.

The Hillsborough meteorite stone was observed under an optical microscope inside a Bassaire laminar flow hood under HEPA-filtered positive pressure (equivalent to ISO Class 4–5) and found to be free of fusion crust. The sample was powdered in a ceramic mortar and pestle inside a laminar flow hood. The sample was homogenized and split into four equal portions of approximately 200 mg each, which were transferred to individual glass ampoules for hot-water extraction. One milliliter of Millipore ultrapure water was added to each sample. The ampoules were then flame-sealed and heated to 100°C for 24 hours in a heating block. After cooling to room temperature, the ampoules were snapped open and centrifuged for 5 min. Ten % of the water supernatant was preserved frozen (−80°C) for carboxylic acid analysis, and 45% was transferred to small test tubes (12 mm by 75 mm) individually, dried under vacuum, flame-sealed in larger test tube (20 mm by 150 mm) containing 1 ml of 6 N HCl, and then subjected to acid vapor hydrolysis for 3 hours at 150°C to determine the total (free + bound) amino acid content. The remaining water supernatant (the nonhydrolyzed fraction) was transferred to separate small test tubes. The residual meteorite powders were washed twice with 1 ml of ultrapure water, and the supernatant was transferred, combined, and dried under vacuum. After the hydrolysis procedure, the test tubes were rinsed with ultrapure water and then cracked open. The small test tubes were removed and dried under vacuum.

Cation exchange was performed on prepacked columns. The columns were prepared according to the following procedures. After removing the caps and snapping off the seals on the Luer tips, the columns were filled to the top with water (~10 ml) plus one bed volume (~2 ml). Once the volume of water was just above the resin bed, three bed volume (~6 ml) of 2 M NaOH was added to desorb any contaminating amino acids. The columns were then washed by filling to the top with water twice (20 ml) until the eluting solution has a neutral pH to remove residual NaOH. Three bed volume (~6 ml) of 1.5 M HCl was added to reacidify the columns. The columns were again washed with two bed volumes of water (20 ml) to remove excess HCl until neutral pH.

Both hydrolyzed and nonhydrolyzed samples were then brought up in 3 × 1 ml of ultrapure water and desalted on the cation exchange resin. Different fractions of the same sample were recombined by desalting in the same column. Purified amino acids were eluted by adding 2 × 3.5 ml fractions of 2 M NH_4_OH, and the eluates were collected in small test tubes, which were then evaporated to dryness by vacuum centrifugation.

Before GC-MS analysis, amino acids were derivatized by esterification with IPA and acylation with TFAA. The samples were resuspended in 2 × 50 μl of ultrapure water in inserts within GC vials. One hundred microliters of acetyl chloride:IPA mixture (30:70 v/v) was added to each of the samples. The vials were tightly capped, and the samples were heated in a heating block set at 110°C for 1 hour. The samples were then cooled in an ice bath and dried under a gentle stream of dry N_2_. After the samples were brought to room temperature, 100 μl of DCM and 50 μl of TFAA were added to the dried sample. The vials were capped tightly again and heated to 100°C for 10 min. The samples were then cooled to room temperature, and the excess reagent was removed under a slow stream of N_2_. Before injection, the derivatized samples were dissolved in 30 μl of DCM and 5 μl of pyrene in DCM (200 μg/ml) as an internal standard. The derivatized samples were then immediately analyzed by GC-MS.

Amino acids in the hot water extracts were analyzed by an Agilent Technologies 7890A series GC coupled to an Agilent Technologies 5975C mass selective detector (MSD). The separations of the d–, l–amino acid enantiomers were achieved using a CP-Chirasil-L Val GC Column (25 m–by–0.25 mm ID by 0.12 μm; Agilent Technologies). For d, l-isovaline enantiomers separation a 6890N series GC coupled to a 5973 MSD (both Agilent Technologies) and a CP-Chirasil-Dex CB GC Column (25 m–by–0.25 mm ID × 0.25 μm; Agilent Technologies) were used.

Helium was used as carrier gas, the column flow rate was set at 1.1 ml/min, and injection (1 μl) was in split mode (10:1) at 220°C. The source and quadrupole temperatures were maintained at 230° and 150°C, respectively, and the MSD transfer line was heated to 180°C. Standard autotunes with perfluorotributylamine and air/water checks were made on a daily basis. The oven program was set at an initial temperature of 90°C, held for 2 min, and then increased by 5°C/min to 200°C and held for 6 min. GC-MS methods and oven program were the same for both instruments used. Total ion current chromatograms were acquired and analyzed with Agilent Technologies MSD ChemStation (6890-5973) or MassHunter (7890-5975) software. Amino acids present in the meteorite samples were identified by comparison of the retention time and mass fragmentation pattern with a known amino acid standard mixture, and quantification was made by chromatographic data collected in the selected ion monitoring mode. Identification was added by retention time locking of GC method and creation of a custom library from standards, which include retention time and retention indices for the amino acids.

## Supplementary Materials

### This PDF file includes:

Supplementary Materials Tables S1 to S16 Figs. S1 to S28 References
