# Supplementary material for: Meteor over New York City: Brines in a primitive CM asteroid
Source: Sci Adv. 2026 Jul 15;12(29):eaea2105. doi: 10.1126/sciadv.aea2105 (PMC13371919; doi:10.1126/sciadv.aea2105)
Supplement: Supplementary file 1 — Supplementary Materials Tables S1 to S16 Figs. S1 to S28 References [file sciadv.aea2105_sm.pdf]

Science Advances
AAAS

Supplementary Materials for

Meteor over New York City: Brines in a primitive CM asteroid

Peter Jenniskens *et al.*

Corresponding author: Peter Jenniskens, pjenniskens@seti.org

Sci. Adv. **12**, eaea2105 (2026)
DOI: 10.1126/sciadv.aea2105

This PDF file includes:

Supplementary Materials
Tables S1 to S16
Figs. S1 to S28
References

Appendix A. Supplementary Materials

Meteor trajectory and meteoroid orbit

By: Mike Hankey, Peter Jenniskens

The meteoroid trajectory and orbital elements are given in Table S-1. Results for Hillsborough are compared to published results for the two night-time CM2 carbonaceous chondrite falls Winchcombe and Maribo.

Table S-1. Meteoroid trajectory and orbital elements (Equinox J2000).

	**Hillsborough** *This work*	**Winchcombe** *King et al., 2022 (47)*	**Maribo** *Borovička et al., 2019 (70)*
Date	2024-07-16	2021-02-28	2009-01-17
Time (UTC)	15:17:27.60	21:54:15.88	19:08:27.41
Solar longitude (º)	114.2392	340.2435	297.6885
Trajectory:			
First Height (km)	44.4 ± 0.5	90.623 ± 0.038	114.9 ± 0.2
First Latitude (ºN)	40.567 ± 0.003	51.87106 ± 29m	54.584 ± 0.003
First Longitude (ºE)	-74.188 ± 0.005	-3.10932 ± 17m	+13.719 ± 0.003
Last Height (km)	34.9 ± 0.5	27.554 ± 0.028	30.6 ± 0.3
Last Latitude (ºN)	40.530 ± 0.003	51.94011 ± 33m	54.711 ± 0.003
Last Longitude (ºE)	-74.390 ± 0.005	-2.09634 ± 11m	+11.592 ± 0.006
Radiant and speed:			
Apparent V∞ (km/s)	14.4 ± 0.6	13.547 ± 0.008	28.3 ± 0.3
Apparent R.A. (º)	164.8 ± 0.3	-.-	123.5 ± 0.3
Apparent Dec. (º)	+28.5 ± 0.1	-.-	+21.76 ± 0.15
Azimuth from N (º)	256.0 ± 0.3	263.342 ± 0.046	95.9 ± 0.3
Elevation (º)	29.1 ± 0.2	41.919 ± 0.029	31.2 ± 0.2
Geocentric V (km/s)	8.6 ± 1.1	8.123 ± 0.013	25.8 ± 0.3
Geocentric R.A. (º)	178.6 ± 9.0	56.638 ± 0.017	125.0 ± 0.3
Geocentric Dec. (º)	+19.9 ± 7.2	+17.713 ± 0.069	+19.8 ± 0.2
Orbital elements:			
a (AU)	2.13 ± 0.33	2.5855 ± 0.0077	2.43 ± 0.12
e	0.533 ± 0.074	0.6183 ± 0.0011	0.805 ± 0.010
q (AU)	0.995 ± 0.009	0.986839 ± 0.000012	0.475 ± 0.005
i (º)	4.1 ± 2.1	0.460 ± 0.014	0.25 ± 0.16
**ω** (º)	159.8 ± 4.2	351.798 ± 0.018	279.4 ± 0.6
Node (º)	114.29 ± 0.07	160.1955 ± 0.0014	297.46 ± 0.15
Last Perihelion	2024-06-29	2021-02-22.446	2005-05-16.0

Doppler weather radar reflections from falling meteorites
By: Marc D. Fries

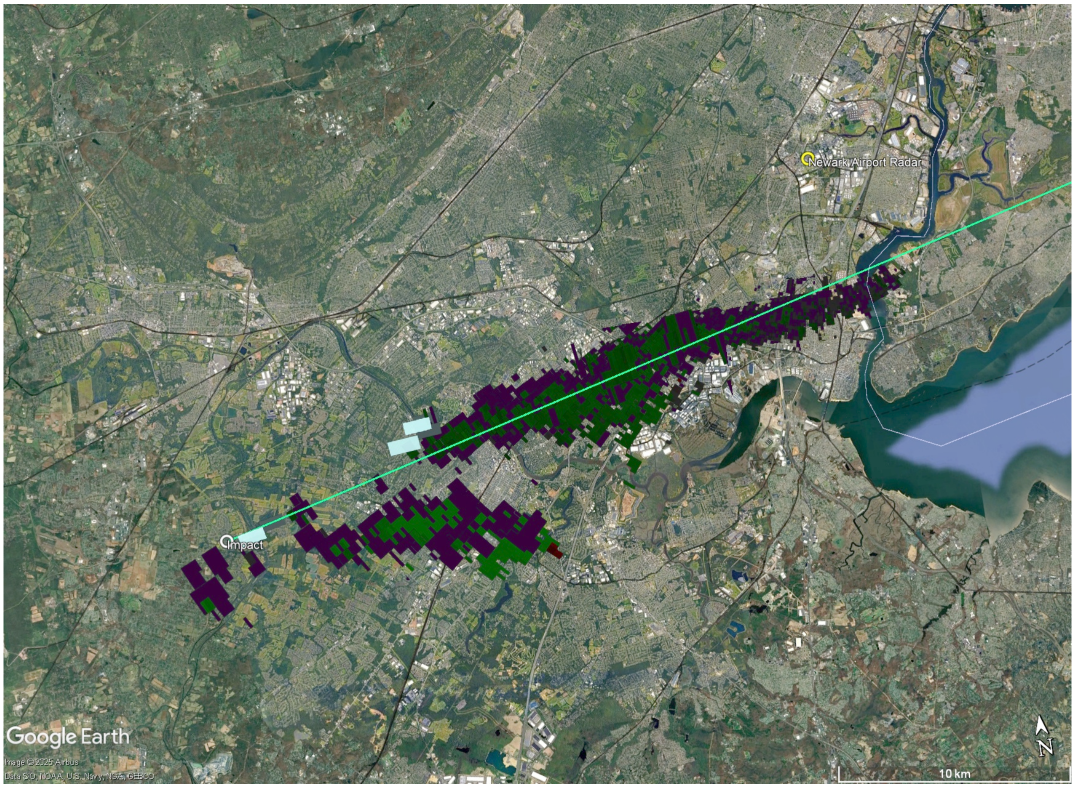

Fig. S-1. Combined ground-projected radar reflections from the Hillsborough meteorite fall. In light blue are the three NEXRAD-detected reflections, in dark colors those generated by the nearby TEWR (Newark Airport TDWR) radar, the location of which is identified at upper right. The green line is the ground-projected path of the fireball. The first radar signature to appear was that at far left and additional signatures appeared drifting ENE over a period of 14 minutes 58 seconds afterward, when smaller meteorites fell to lower altitudes under the influence of prevailing winds. Attribution in lower left corner reads: Google Earth. Image ©2025 Airbus. Data SIO, NOAA, U.S. Navy, NGA, GEBCO.

Falling material was first detected by the TEWR radar in the TDWR network which serves the Newark Liberty National Airport. The TEWR radar lies only 5-40 km laterally from the fall as measured from the nearest and farthest radar signatures (Figs. S-1, S-2). The first radar detection occurred at 15:18:52.0 UTC and an altitude of 13.339 km above mean sea level (AMSL). This is 84 s after the fireball terminus. TEWR recorded falling meteorites in an impressive 25 radar sweeps, with the last one recorded at 15:33:50.0 UTC and 6.4 km AMSL. The total elapsed time of detection is 14 minutes 58 seconds.

NEXRAD detections were sparse, with only a trio of pixels appearing in data from the KDIX (Fort Dix, NJ) radar (light blue in Fig. S-1). One is close to the fall location.

According to the Jörmungandr dark flight model (*71*) and using radiosonde (or "weather balloon") data from Upton, NY collected at 1200 UTC on 16 July 2024, the last material detected on radar is approximately 0.5 mm in diameter (~0.1 g) assuming spheres with density of 1.89 g/cc. The first material seen corresponds to meteorites of approximately 10 g in mass.

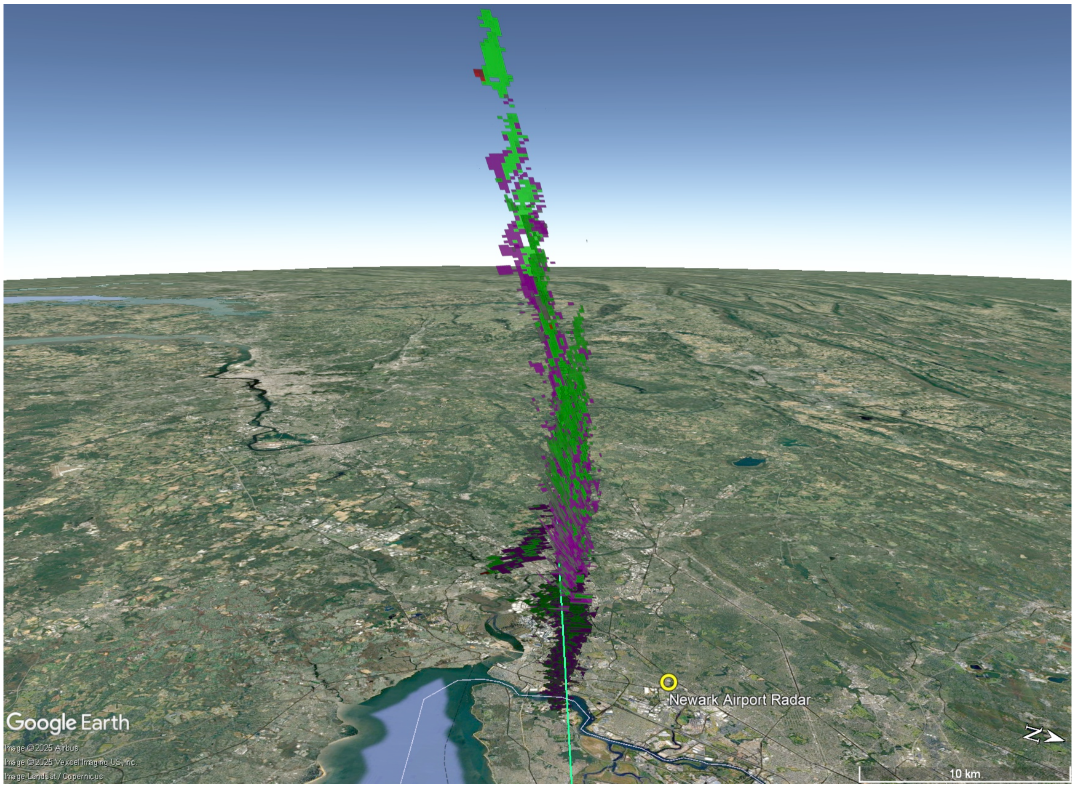

Fig. S-2. The same data as that in Fig. S-1 but viewed from an approximate 45-degree elevation along the direction of flight of the bolide. The highest altitude of a radar signature measures 13.339 km above mean sea level (AMSL) at the signature centroid and the lowest occurs at 6.470 km ASML. Attribution in lower left corner reads: Google Earth. Image ©2025 Airbus. Image ©2025 Vexel Imaging US, Inc. Image Landsat / Copernicus.

Petrography

By: Michael E. Zolensky, Jangmi Han, Loan Le, Marc D. Fries

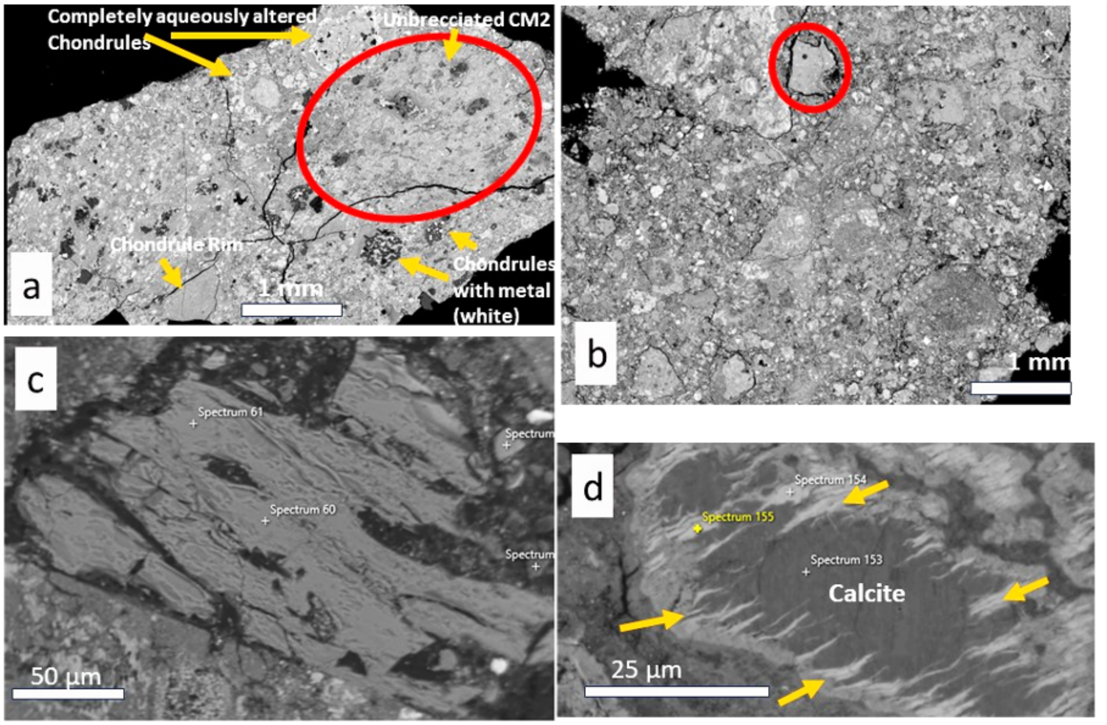

Fig. S-3. Backscattered electron images of Hillsborough. **(A)** Comminuted CM material with one of the larger pieces of unbroken CM2 lithology circled. One fine-grained chondrule rim, two completely aqueously altered chondrules, and two chondrules with remnant metal are also indicated. **(B)** A thoroughly-comminuted sample, containing one of the C1 clasts (circled). This same clast is shown in Figures 2 and 3 in the main paper. **(C)** Partially-dissolved olivine crystal. **(D)** Calcite crystal rimmed and partially penetrated by tochilinite flakes (arrowed).

The interior of the stones are dark gray to black and sparsely decorated with light-colored grains. The meteorite is unusually finely brecciated for a CM. The largest continuous fragments observed were 5 mm, and some investigated stones consist entirely of finely comminuted, sub-mm sized fragments (see Fig. S-3).

The stones were exceptionally difficult to polish owing to extreme friability. Tests revealed that some samples would rapidly disaggregate upon contact to water, alcohol and oil. Therefore, no fluids were used for cutting sample preparation, and so all polishing had to be performed dry. This behavior was similar to that we had previously observed for the Tarda chondrite. The result was that very poor, badly-scratched surfaces had to be used for EPMA, resulting in slightly reduced analytical accuracy.

CM Lithology

SEM/EDS/EPMA observations and measurements revealed that the bulk matrix is dominated by serpentine, tochilinite, calcite, pyrrhotite, pentlandite, and, to a lesser degree, Fe-Ni metal. Rare matrix phases are Ca-phosphate, magnetite and chromite. Intergrowths of serpentine and tochilinite are common, as is usual for CMs. Fragments of olivine and lesser low-Ca pyroxene are abundant in the matrix, and these exhibit considerable etching by aqueous fluids (see Fig. S-3B). The range of serpentine compositions spanned the entire range observed by the analyses for all other CMs (Fig. S-3B). Metal is mostly confined to small grains within olivine crystals. Aggregates and intergrown (exsolved) crystals of pyrrhotite and pentlandite are abundant. One rather unusual pentlandite crystal was found to contain 11 atomic% Mn.

Sinuous Ca-Al-rich inclusions (CAIs) comprise approximately 2% of the examined samples. These consist of spinel with inclusions of perovskite and are rimmed by probable diopside (the latter being too fine-grained for accurate EPMA measurements). These particular CAIs are typical for CM chondrites.

Chondrules and chondrule pseudomorphs are abundant, and most are fragmented to varying degrees. Most chondrules exhibit thick fine-grained rims, frequently multilayered with respect to bulk FeO content. Chondrules range up to 1 mm in diameter, but the majority are less than 100 µm in diameter. Porphyritic chondrule types predominate. The chondrules and silicate fragments show a range of alteration to hydrous phases and many completely lack anhydrous silicates (Fig. S-3A). There is excellent pseudomorphism of serpentine, tochilinite and calcite after primary ferromagnesian minerals. Fine-grained clasts are abundant, and appear to actually be fine-grained chondrule rims viewed along a plane that does not intersect the underlying chondrule (Fig. S-3A).

Olivine shows a wide compositional range from Fa_2 to Fa_{33} (n=8), with CaO up to 0.42 wt%, Cr_2O_3 up to 0.44 wt%, NiO up to 0.084 wt%, and Al_2O_3 up to 0.20 wt%. As is typical for CMs, calcite is generally fairly pure but some examined crystals contained up to 1.5 wt% MgO and 2.5 wt% FeO. Chromites contain up to 11.5 wt% Al_2O_3 and 3.1 wt% TiO_2.

C1 Lithology

A notable feature is the presence of a few small (<500µm), matrix-dominated clasts of C1 material. The bulk matrix in this lithology is still poorly characterized, but contains up to 2 wt% Na_2O, which is very unusual. EPMA measurements of the matrix (Table S-2) are consistent with its being serpentine (Fig. 2, main text), with a rather high Na content averaging 5.15 wt% Na_2O. Lee at al. (*72*) have recently reported sodium-bearing phosphates in a CM1 clast in the Cold Bokkeveld CM2 chondrite, but the C1 clasts in Hillsborough contains uniformly sodium-enriched phyllosilicate *matrix*. Since the phyllosilicate in this matrix is serpentine, the sodium must be some finely dispersed carrier phase containing only light elements such as oxides or carbonates, but not a phosphate. In fact, we found that scattered within the

fine-grained matrix of the Hillsborough C1 clast are crystals of dolomite and magnetite (plaquettes and framboids). The dolomites are rimmed by matrix enriched in Na (Fig. 2). To investigate the nature of this Na-rich phase we cut a focused ion beam (FIB) slice across one of the dolomites (Fig. 3). TEM observation revealed that the dolomite was a single continuous crystal, which was cut by numerous fractures (Fig. 3C). X-ray mapping revealed that the fractures are filled with a Na-rich, amorphous phase. It is likely that the phase was rendered amorphous by the electron and X-ray techniques.

Table S-2. EPMA analysis of phases in the C1 lithology (wt.%).

	Serpentine[1]	Dolomite[2]
Na_2O	5.15	10.46
SiO_2	34.38	0.92
MgO	16.69	18.36
Al_2O_3	0.88	0.07
P_2O_5	0.04	nd
S	0.51	0.16
K_2O	nd*	0.20
CaO	0.08	24.38
TiO_2	0.02	0.01
MnO	0.06	0.58
Cr_2O_3	0.09	0.02
FeO	31.59	5.92
CoO	0.04	0.02
NiO	0.14	0.08
Total	89.66	61.19

Notes: [1]Average of 3 measurements; [2]Average of 2 measurements; *nd: Below the detection limit.

Classification: CM1/2. The dominant lithology contains areas with chondrules almost completely replaced by hydrous silicates and intimately associated and mixed with chondrules and ferromagnesian mineral fragments partially replaced by serpentine, tochilinite and calcite (CM1/2), to areas more typical of CM2 meteorites (olivine and metal present). The bulk mineralogy is consistent with CM1 to CM2 meteorites.

The C1 clasts in Hillsborough have a mineralogy consistent only with CM1, whose mineralogy has been well described previously (*72–75*). With only two rare exceptions (see below), the matrix phyllosilicates in all other known C1 lithologies, including CI1 (including Ryugu and Bennu samples), CR1, and C1 clasts in non-carbonaceous meteorites (notably HEDs, ordinary chondrites, and ureilites) all contain an intimate mixture of serpentine and saponite rather than solely serpentine. The exceptions to this are a single unique type 1 chondrite Flensburg (*74*) and a single

CR1 chondrite GRO (*75*), both of which contain serpentine as the dominant phyllosilicate phase. Interestingly, Flensburg's serpentine contains up to 2 wt% Na_2O, which is also unusually high for this phase but still less than half the sodium content of serpentine in the Hillsborough C1 lithology. In addition, CM1 clasts are well known from CM2 meteorites, whereas CI1 or CR1 clasts are unknown from CM2s. Thus, we are confident in proposing that the C1 clasts in Hillsborough are CM1. Verification of this classification would necessarily involve very involved oxygen isotope measurements by secondary ion mass spectrometry (SIMS), beyond the scope of this preliminary investigation. This is because SIMS measurements of phyllosilicate bearing materials are notoriously difficult.

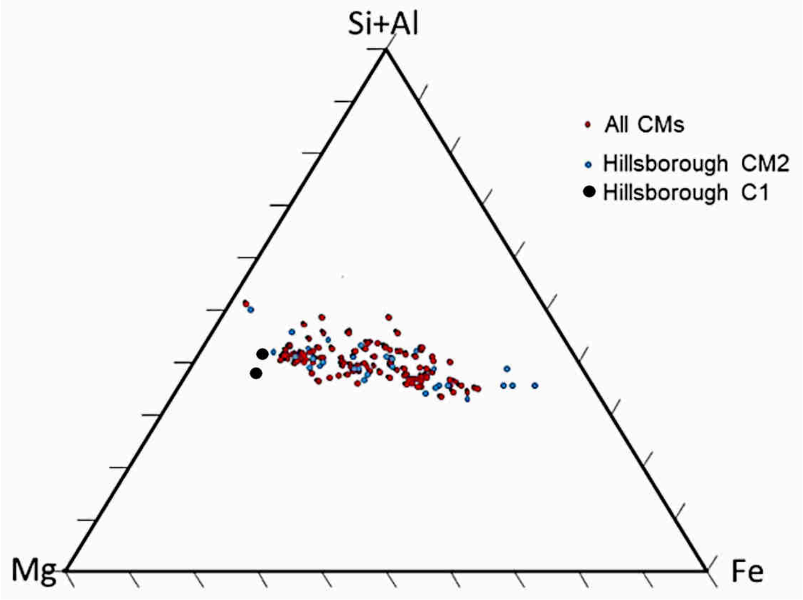

Fig. S-4. Atom percent plot of Hillsborough CM1/2 phyllosilicates (blue) and Hillsborough C1 clast (large •) compared to serpentine from several CM2 chondrites (red). It is interesting that the Hillsborough CM2 serpentine compositions span the entire range observed for other CM chondrites, while C1 clast serpentine compositions plot outside this range.

X-ray Computed Tomography
By: Eva M. Riveros, Jon M. Friedrich, and Denton S. Ebel

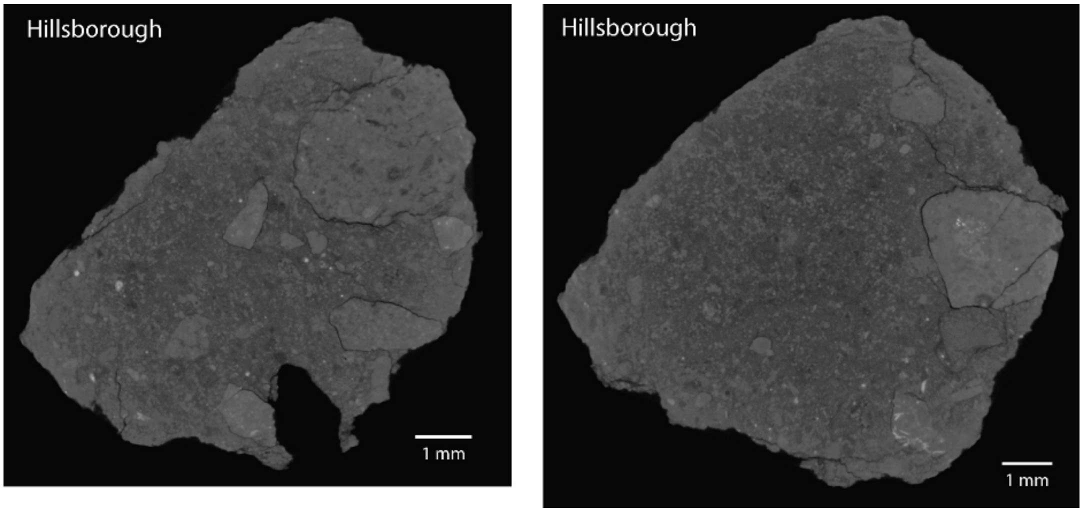

Fig. S-5. Two µCT "slices" of central portions of the same ~1g chip of the Hillsborough carbonaceous chondrite. Within these images, lighter greyscale values represent greater x-ray attenuation. The air around the sample and the low atomic weight material surrounding some clasts are represented by the darkest greyscale values while silicates are depicted by lighter greyscale values. Clasts of variable composition can be readily distinguished from others and from the surrounding heavily comminuted material. What are likely unbrecciated CM clasts are often surrounded by low average atomic weight material, probably air.

A 0.9946 g sample of the Hillsborough CM chondrite was imaged. Digitally isolating the chondrite yields a volume of 0.525 cm^3 and gives a resulting bulk density of 1.89 g/cm^3. This bulk density is near the lower end of the full range (1.88 – 2.47 g/cm^3) for CM chondrite bulk density reported by (*76*). Assuming the mean CM grain density of 2.92 g/cm^3 (*76*), the corresponding porosity is 35.3%. This lies at the higher end of porosity range (15.0 – 36.7%) reported for CM chondrites by (*76*). The low bulk density and high porosity of Hillsborough are probably due to the substantial void space (cracks) surrounding some clasts in the sample (Fig. S-5) in addition to the substantial unresolvable microporosity that exists in volumes below the resolution (8.0 µm/voxel) of our CT image scans.

The ~10 mm diameter fragment of Hillsborough contains many obvious clasts of material that appear to be unbrecciated, likely representing the CM unbrecciated material seen in SEM images of Hillsborough thin sections (cf. Fig. S-5). These clasts are rimmed by low X-ray attenuation material and we interpret this as porosity (air) rather than another low-Z material such as a carbon-rich component. We have digitally isolated these clasts and a 3D representation of them is shown in Fig. S-6. They comprise only 7% of the total volume of the ~ 10 mm diameter Hillsborough sample. These mostly sub-equant spheroidal clasts (Fig. S-7) possess shapes like those of other melt free impact breccias such as those found in CI chondrites (*77*).

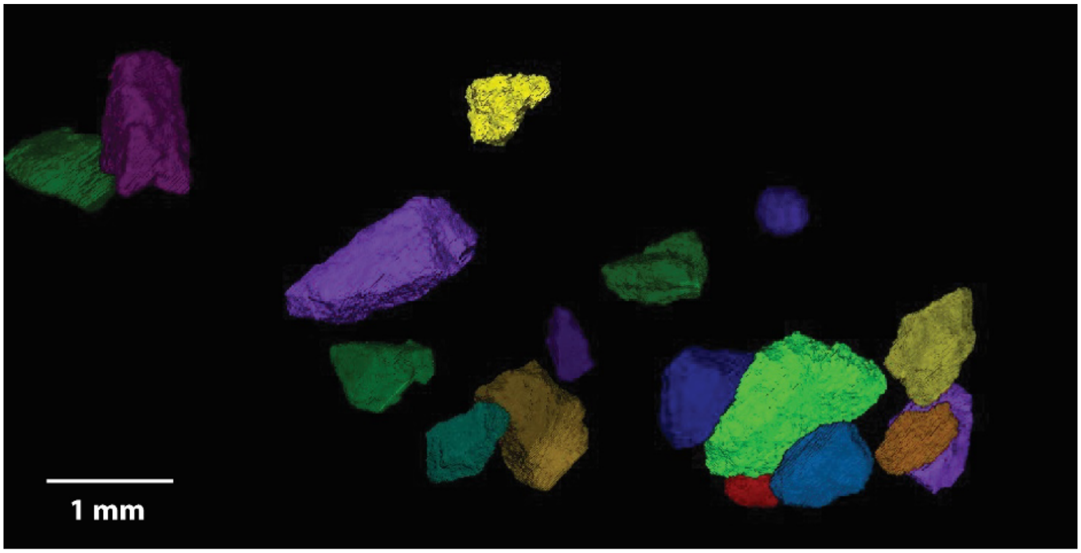

Fig. S-6. Digitally isolated clasts within the ~10 mm diameter Hillsborough chondrite subsample shown in Fig. S-5. These clasts are likely unbrecciated CM chondrite material.

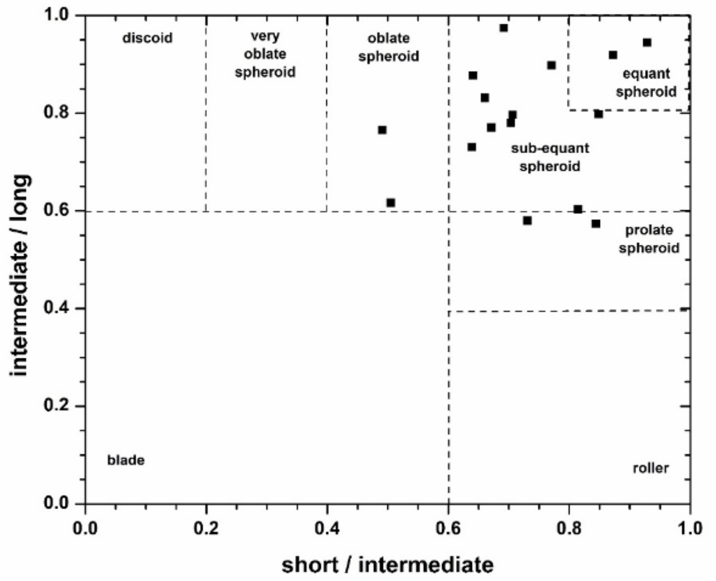

Fig. S-7. Zingg diagram (*51*) using axial ratios to examine unbrecciated chondrite clast shapes in Hillsborough using the terminology of (*52*). These shape data are based on best-fit ellipsoids constructed around each clast using Blob3D. The mostly sub-equant spheroidal clasts possess shapes like melt-free impact breccias on other solar system bodies.

Oxygen isotopes
By: Karen Ziegler

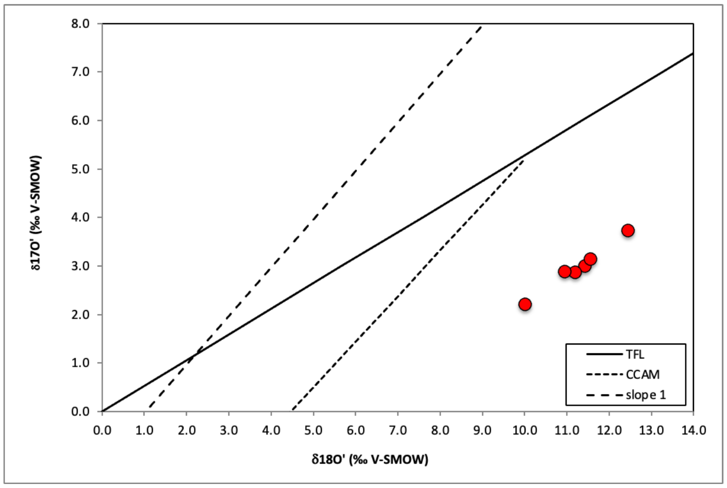

Fig. S-8. Oxygen isotope values for Hillsborough (red points) in a diagram with the Terrestrial Fraction Line (TFL), Carbonaceous Chondrite Anhydrous Mineral (CCAM) line, and a "slope 1" displaced from the CCAM line.

The results (Table S-3, Fig. S-8) all lie within the CM carbonaceous chondrite field (*15*). The range was $\delta^{18}O$ = 10.010 to 12.453 ‰, $\delta^{17}O$ = 2.205 to 3.730 ‰, and $\Delta^{17}O$ = -3.081 to -2.845 ‰, respectively. The average values are $\delta^{18}O$ = 11.265 ‰, $\delta^{17}O$ = 2.973 ‰, and $\Delta^{17}O$ = -2.975 ‰ (linearized with Terrestrial Fractionation Line slope = 0.528).

Table S-3. Oxygen isotopes.

ID	Mass (mg)	Date	$\delta^{17}O$ (‰)	$\delta^{18}O$ (‰)	$\Delta^{17}O$ (‰)	n	$\Delta^{17}O$ (‰)
1	4.60	20-Aug-24	3.002	11.429	-3.032	20	-2.941
2	5.30	20-Aug-24	3.730	12.453	-2.845	20	-2.746
3	3.80	20-Aug-24	2.875	11.192	-3.035	20	-2.945
4	4.70	20-Aug-24	3.139	11.564	-2.967	20	-2.874
5	4.90	21-Aug-24	2.205	10.010	-3.081	20	-3.000
6	7.40	21-Aug-24	2.887	10.944	-2.892	20	-2.804

Titanium isotopes
By: Gregory A. Brennecka and Jan H. Render

Isotope data in Table S-4 are reported in the epsilon notation, as relative deviations in parts per ten thousand from the OL-Ti bracketing standard:

$$\varepsilon^{i}Ti = \left(\frac{{}^{i}Ti/{}^{47}Ti}{{}^{i}Ti/{}^{47}Ti} - 1 \right) \times 10{,}000 \qquad (1)$$

Figure S-9 shows the Titanium isotopic composition of Hillsborough in relation to those of carbonaceous chondrite types.

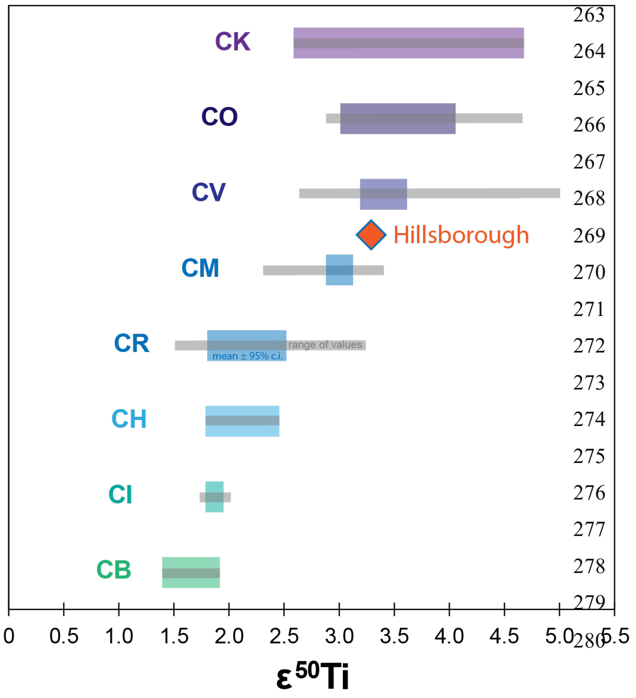

Fig. S-9. Titanium isotopic composition of Hillsborough in relation to those of carbonaceous chondrite types. Literature data shown was compiled in (*16*), where the colored boxes represent the average with a 95% confidence interval, and the gray bars represent the range of data in each meteorite group. Where sample sizes are small (e.g., CK, CH, CB), the most conservative uncertainties are shown. Chemical separation and measurement methods follow (*53*) with the modification of 8 s integrations as opposed to 4 s integrations.

Table S-4. Titanium isotopes for Hillsborough.

	ε^{46}Ti	ε^{48}Ti	ε^{50}Ti
Hillsborough.1	0.60	0.00	3.21
Hillsborough.2	0.50	-0.06	3.29
Hillsborough.3	0.63	0.06	3.40
Hillsborough.4	0.55	-0.07	3.38
Hillsborough.5	0.64	-0.04	3.16
Hillsborough.6	0.59	0.00	3.33
AVG	0.59	-0.02	3.30
2SD	*±0.11*	*±0.10*	*±0.19*
2SE	*±0.04*	*±0.04*	*±0.08*

Reflectance spectroscopy
By: Takahiro Hiroi

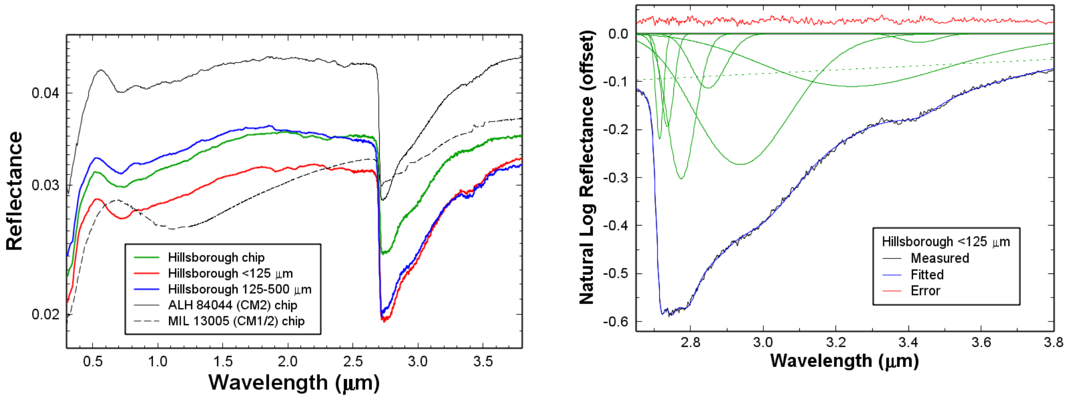

Fig. S-10 (A, left). VNIR-FTIR combined reflectance spectra of Hillsborough chip and powders along with those of other CM chondrite chips (*17, 54*). **(B, right).** Gaussian fitting of the 3-µm hydration band of a Hillsborough spectrum shown as an example. A continuum background (linear to wavelength) is shown in a green broken line, and Gaussians (in wavenumber) are shown in green solid lines.

As shown in Fig. S-10A, all the spectra of Hillsborough samples are highly consistent with one another and with typical CM2 chondrite spectra but dissimilar to the spectrum of this particular CM1/2 chondrite (a find). In addition, Gaussian fitting of the samples (Fig. S-10B) reveals that its 2.7-µm OH absorption band position and shape are consistent with those of both CM2 and CM1-1/2 chondrites (Fig. S-11). Therefore, the Hillsborough meteorite is spectrally CM type.

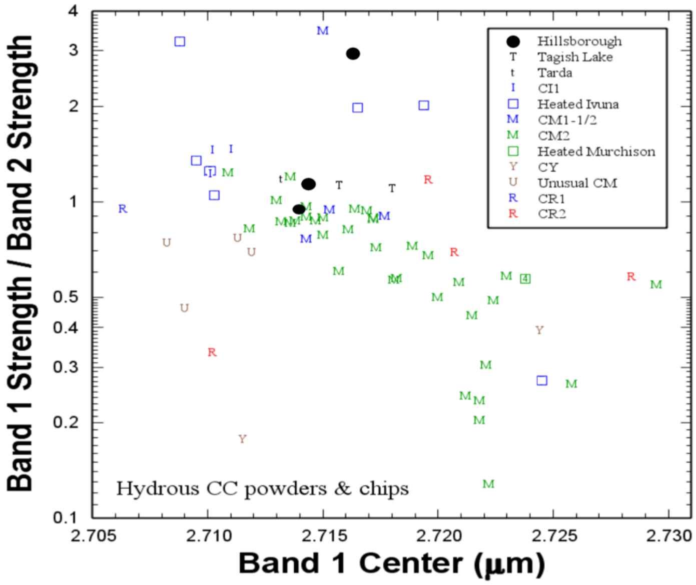

Fig. S-11. A plot of 2.7-µm band position and shape of the Hillsborough meteorite (•) in comparison with other carbonaceous chondrites (*17, 54*).

Cosmogenic radionuclides.

By: Kees C. Welten, Kunihiko Nishiizumi, Marc W. Caffee

Table S-5 shows results of the two samples; the ^{26}Al AMS measurement of the larger sample is in progress. Assuming expected ^{10}Be and ^{26}Al production rates in CM chondrites of ~22 and ~42 dpm/kg, respectively, for an object with a radius of ~20 cm the measured ^{10}Be and ^{26}Al concentrations in the Hillsborough CM chondrite indicate a 4π CRE age (as a small object in space) of 0.23 ± 0.02 Myr, overlapping with one of the main CRE age clusters of 0.2 Myr for CM chondrites (*18*). With this short CRE age, the ^{36}Cl concentration has reached only ~40% of the saturation value, so the measured ^{36}Cl concentration of ~9 dpm/kg corresponds to a ^{36}Cl production rate of ~22 dpm/kg. This value is much higher than the value of ~8 dpm/kg expected from spallation reactions on K, Ca, Fe and Ni, indicating that the Hillsborough meteorite contains a significant contribution of neutron-capture produced ^{36}Cl, which is consistent with observations in many other CM chondrites.

Table S-5. Concentrations of major elements (measured by ICP-OES) and of cosmogenic ^{10}Be, ^{26}Al and ^{36}Cl (measured by AMS) in two aliquots (5.10 mg and 50.04 mg) of Hillsborough CM chondrite.

**Element**	**5.10 mg**	**50.04 mg**
Mg (%)	11.4	11.5
Al (%)	1.09	1.13
P (%)	0.11	0.12
S (%)	3.4	3.6
K (ppm)	566	480
Ca (%)	1.67	1.26
Ti (ppm)	618	584
Mn (%)	0.18	0.17
Fe (%)	20.7	20.6
Co (ppm)	587	562
Ni (%)	1.25	1.22
**Nuclide**		
^{10}Be [dpm/kg]	2.58 ± 0.05	2.53 ± 0.04
^{26}Al [dpm/kg]	8.36 ± 0.23	-
^{36}Cl [dpm/kg]	10.4 ± 0.5	8.77 ± 0.16

Noble gases
By: Henner Busemann, Daniela Krietsch, Colin Maden

The light noble gases are dominated by a large contribution of solar wind (SW). The concentrations of ^{4}He and, particularly, ^{20}Ne are among the highest observed in CM chondrites (cf. *19*, and references given therein). This implies a comparatively long exposure of some of the examined Hillsborough material in the upper regolith layers of its parent body - or an unusual high fraction of SW-bearing grains in our sample. This is also reflected in the $^{3}He/^{4}He$ and $^{20}Ne/^{22}Ne$ ratios (Table S-6A) that almost reach the ratios measured in pure SW as, e.g., returned by the Genesis mission (*20*). Hillsborough is, thus, a regolith breccia, consistent with its comminuted character.

A comparatively long presence of the examined material at the surface of the parent asteroid also implies that some near-surface "pre-exposure" of the material on the parent body, in addition to the exposure to cosmic rays during its transfer through space (so-called "complex exposure history") cannot be excluded. This could include both exposure to galactic cosmic rays (GCRs, detectable in the upper few m within a regolith) and solar cosmic rays (SCRs, within the upper few cm). Due to the abundant SW presence, the $(^{21}Ne/^{22}Ne)_{cos}$ ratio cannot be used as a shielding indicator nor to detect possible SCR-derived Ne_{cos}.

Using the pre-atmospheric mass of Hillsborough of 53 ± 6 kg, the density of 1.89 ± 0.01 g/cm^3 (both this work), and assuming a spherical shape, its pre-atmospheric radius would have been 18.1–19.6 cm. Next to the bulk chemistry taken from this work (Tables S-5, S-7, S-8) and for Si and O from (*78*), the radius is one of the two geometric shielding parameters used in the (*79*) model to calculate potential cosmogenic noble gas production rates. To include a potential non-spherical shape, we chose pre-atmospheric radii of 10, 20 and 25 cm as input. We furthermore restricted possible shielding depths of our sample to 78 % of these radii, i.e. 8, 16 and 20 cm. The probability that our sample originates from the other, inner 22 % of the sphere is less than 1 % of the total volume (*79*). However, this did not change the determination of the production rate range given below.

Combining all information (radius, depth, chemistry), the production rate P_{21} for $^{21}Ne_{cos}$ is in the range 0.1199 and 0.2256 cm^3 STP ^{21}Ne / (g × Ma). The resulting nominal 4π (i.e., in space) total GCR exposure age would be 2.2 to 5.7 Ma. As discussed, additional exposure on the parent body cannot be excluded. If this pre-exposure occurred in the past (i.e., > 10 half-lives of ^{10}Be and ^{26}Al before ejection), it will not be monitored by these radionuclides anymore, while the noble gases were accumulated during both irradiation periods. The transfer time in space will then be given by the radionuclides.

The ^{36}Ar, ^{84}Kr, and ^{132}Xe concentrations as well as the $^{36}Ar/^{132}Xe$ and $^{84}Kr/^{132}Xe$ ratios are in the typical range observed in moderately to strongly aqueously altered CM chondrites that have lost most of their "Ar-rich" noble gas component that is susceptible to aqueous alteration (*19*), which agrees with Hillsborough's classification as CM1/2 chondrite. The Xe isotopic composition can be explained by

Q-Xe with a minor addition of ~2 % of Xe-HL (based on ^{132}Xe), some excess ^{129}Xe from the decay of short lived ^{129}I and probably some SW-Xe only visible in ^{124,126}Xe. Similarly, Kr is isotopically similar to Q-Kr with a minor addition of SW-Kr.

Table S-6A. Helium and Ne concentrations (in 10^{-8} cm^3 STP/g) and isotopic ratios.

Sample	^{4}He	^{3}He/^{4}He x 10^4	^{20}Ne	^{21}Ne$_{cosm}$	^{20}Ne/^{22}Ne	^{21}Ne/^{22}Ne
Hillsborough	104430<br>± 700	4.074<br>± 0.034	1579<br>± 40	0.58<br>± 0.10	13.40<br>± 0.33	0.03695<br>± 0.00009

Table S-6B. Argon concentrations (in 10^{-8} cm^3 STP/g) and isotopic ratios.

Sample	^{36}Ar	^{36}Ar/^{38}Ar	^{40}Ar/^{36}Ar
Hillsborough	124.6<br>± 2.1	5.188<br>± 0.018	7.22<br>± 0.15

Table S-6C. Krypton concentrations (in 10^{-10} cm^3 STP/g) and isotopic ratios.

Sample	^{84}Kr	^{78}Kr/^{84}Kr	^{80}Kr/^{84}Kr	^{82}Kr/^{84}Kr	^{83}Kr/^{84}Kr	^{86}Kr/^{84}Kr
		^{84}Kr = 100				
Hillsborough	83.96<br>± 0.69	0.603<br>± 0.009	3.933<br>± 0.021	19.96<br>± 0.08	20.02<br>± 0.08	30.76<br>± 0.12

Table S-6D. Xenon concentrations (in 10^{-10} cm^3 STP/g) and isotopic ratios.

Sample	^{132}Xe	^{124}Xe/^{132}Xe	^{126}Xe/^{132}Xe	^{128}Xe/^{132}Xe	^{129}Xe/^{132}Xe	^{130}Xe/^{132}Xe	^{131}Xe/^{132}Xe	^{134}Xe/^{132}Xe	^{136}Xe/^{132}Xe
		^{132}Xe = 100							
Hillsborough	98.48<br>±0.51	0.4707<br>±0.0056	0.4111<br>±0.0035	8.273<br>±0.047	107.23<br>±0.43	16.062<br>±0.061	82.08<br>±0.28	38.00<br>±0.15	32.08<br>±0.13

Paleomagnetic and rock magnetic analysis
By: Sonia M. Tikoo & Ji-In Jung

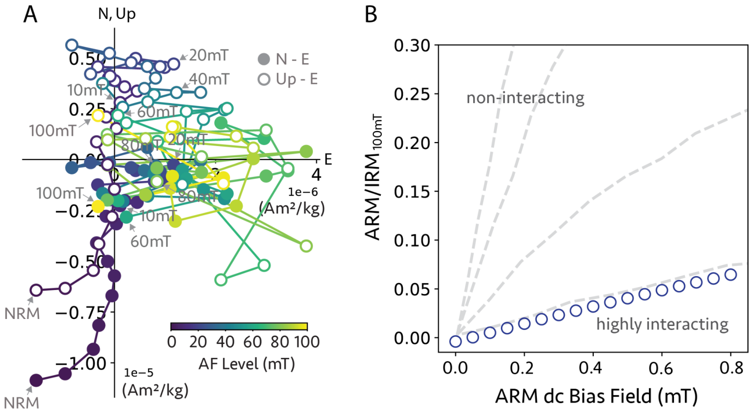

Fig. S-12. Magnetic results from the Hillsborough meteorite. **(A)** Vector-endpoint diagram showing AF demagnetization data. **(B)** ARM acquisition curve for the sample. From top to bottom, the gray dashed curves represent ARM acquisition curves for different reference materials: non-interacting single-domain magnetite, ultrasonic-treated magnetotactic bacteria, detergent-treated magnetotactic bacteria, and highly interacting chiton tooth magnetite (*80*).

The NRM was $1.27x10^{-5}$ Am^2/kg. A low coercivity component was removed below applied fields of 10 mT, and the remanence was unstable at higher AF levels (Fig. S-12A). The absence of high coercivity remanence in the sample, coupled with a low NRM/sIRM ratio of $1.23x10^{-4}$ suggests that Hillsborough may have experienced aqueous alteration and formation of its dominant magnetic minerals, magnetite and pyrrhotite, in a paleofield with intensity <~400 nT, using the magnetite chemical remanent magnetization paleointensity calibration from (*81*). The low paleointensity could be due to aqueous alteration on the parent body post-dating the dissipation of the solar nebula field (*21*), or loss of primary magnetic mineral orientations during brecciation. The ARM to IRM ratio may be utilized to assess the degree of magnetostatic interactions within samples, with high and low ratios indicating weak and strong magnetostatic interactions, respectively (*81*). Our sample exhibits highly interacting behavior (Fig. S-12B), consistent with the observation of magnetite plaquettes and framboids and aggregates of pyrrhotite in electron microscopy.

Elemental abundances and isotopes of C, N, and S

By: Nanako O. Ogawa, Yoshinori Takano, Naohiko Ohkouchi

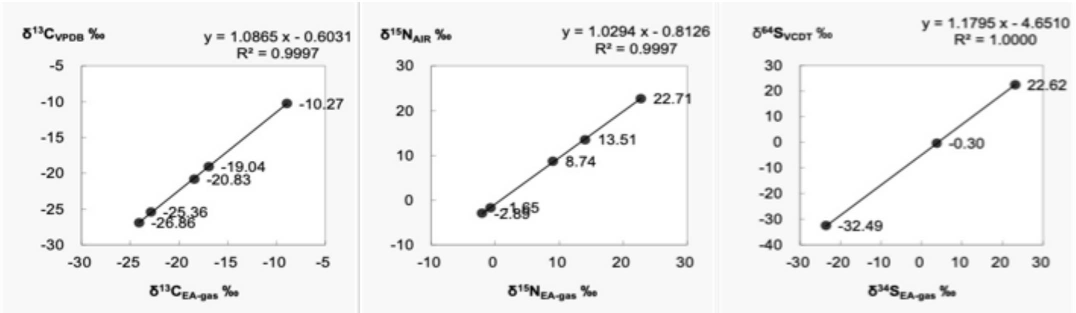

Fig. S-13. Calibration.

Table S-7A. C, N and S isotope standards.

ID	Material	$\delta^{13}C$ (‰ vs VPDB)	$\delta^{15}N$ (‰ vs Air)	$\delta^{34}S$ (‰ vs VCDT)	Reference
CERKU-01	DL-Alanine	-25.36 ± 0.08	-2.85 ± 0.04	-.-	Tayasu et al., 2011 (82)
CERKU-02	L-Alanine	-19.04 ± 0.04	22.71 ± 0.06	-.-	Tayasu et al., 2011 (82)
BG-T	L-Tyrosine	-20.83 ± 0.10	8.74 ± 0.09	-.-	Tayasu et al., 2011 (82)
BG-A	L-Alanine	-26.86 ± 0.04	-1.65 ± 0.06	-.-	Tayasu et al., 2011 (82)
BG-P	L-Proline	-10.27 ± 0.04	13.51 ± 0.02	-.-	Tayasu et al., 2011 (82)
IAEA-S-1	Silver Sulfide	-.-	-.-	-0.30 ± 0.03	IAEA
IAEA-S-2	Silver Sulfide	-.-	-.-	22.62 ± 0.08	IAEA
IAEA-S-3	Silver Sulfide	-.-	-.-	-32.49 ± 0.08	IAEA
Analytical error*	-.-	±0.19 (n=13)	±0.24 (n=12)	±0.74 (n=10)	

*Estimated by repeated analysis of BG-T ($\delta^{13}C$, $\delta^{15}N$) and IAEA-S-1 ($\delta^{34}S$)

Table S-7B. C, N and S isotopes and elemental abundances.

Run	Mass (mg)	C (wt.%)	N (wt.%)	S (wt.%)	$\delta^{13}C$ (‰ vs VPDB)	$\delta^{15}N$ (‰ vs Air)	$\delta^{34}S$ (‰ vs VCDT)
NC-1	0.300	1.70	0.070	-.-	-0.4	+27.8	-.-
NC-2	0.911	1.81	0.075	-.-	+0.1	+28.5	-.-
NC-3	0.954	1.76	0.075	-.-	+0.2	+30.0	-.-
NC-4	0.924	1.77	0.076	-.-	-0.5	+27.7	-.-
S-1	0.013	-.-	-.-	n.d.	-.-	-.-	-1.8
S-2	0.025	-.-	-.-	n.d.	-.-	-.-	-0.3
S-3	0.021	-.-	-.-	n.d.	-.-	-.-	-1.5
S-4	0.107	-.-	-.-	5.94	-.-	-.-	-0.3
S-5	0.109	-.-	-.-	6.09	-.-	-.-	-0.9
S-6	0.112	-.-	-.-	6.37	-.-	-.-	-1.0
Mean		1.76 ± 0.05	0.074 ± 0.030	6.13 ± 0.22	-0.2 ± 0.4	+28.5 ± 1.1	-1.0 ± 0.6

Table S-7B (cont.). C, N and S isotopes and elemental abundances.

Run	Mass Ratio C/N	Mass Ratio N/C	Mass Ratio S/C	Mass Ratio C/S	Date
NC-1	24.29	0.041	-.-	-.-	18-Nov-24
NC-2	24.13	0.041	-.-	-.-	18-Nov 24
NC-3	23.47	0.043	-.-	-.-	18-Nov 24
NC-4	23.29	0.043	-.-	-.-	18-Nov-24
S-1	-.-	-.-	-.-	-.-	29-Nov-24
S-2	-.-	-.-	-.-	-.-	29-Nov-24
S-3	-.-	-.-	-.-	-.-	29-Nov-24
S-4	-.-	-.-	-.-	-.-	29-Nov-24
S-5	-.-	-.-	-.-	-.-	29-Nov-24
S-6	-.-	-.-	-.-	-.-	29-Nov-24
**Mean**	**23.79 ± 0.49**	**0.042 ± 0.001**	**3.48 ± 0.15**	**0.29 ± 0.013**	

Results: The resulting carbon and nitrogen contents and their isotope values ($\delta^{13}C$, $\delta^{15}N$), as well as the sulfur content and $\delta^{34}S$ values, are summarized in Tables S-7B and discussed in the main text.

Bulk elemental abundances of H, C, N, and S
By: Queenie H. S. Chan, James Brakeley, Bianka Munday

Table S-8. Compilation of bulk elemental abundances.

	**H (wt%)**	**N (wt%)**	**C (wt%)**	**S (wt%)**	**Ref.**
Hillsborough (0.011g)	1.292 ± 0.018	0.100 ± 0.015	1.940 ± 0.035	3.076 ± 0.340	This study
Hillsborough (0.010g)	-.-	0.074 ± 0.030	1.76 ± 0.05	6.13 ± 0.22	Table S-7B
Hillsborough (0.055g)	-.-	-.-	-.-	3.5 ± 0.1	Table S-5
Murchison (CM2)	1.135 ± 0.057	0.104 ± 0.003	1.980 ± 0.030	3.40 ± 0.14	*Sephton et al., 2003 (83), Cody et al., 2024 (30)*
Orgueil (CI1)	1.564 ± 0.019	0.208	3.92	5.52 ± 0.13	*Sephton et al. 2003 (83), Alexander et al., 2012 (7)*

The results for bulk elemental abundances are summarized in Table S-8, where data for Hillsborough are compared to literature values of Murchison and Orgueil. The C/H versus C/N ratios of bulk Hillsborough are shown in Fig. S-14.

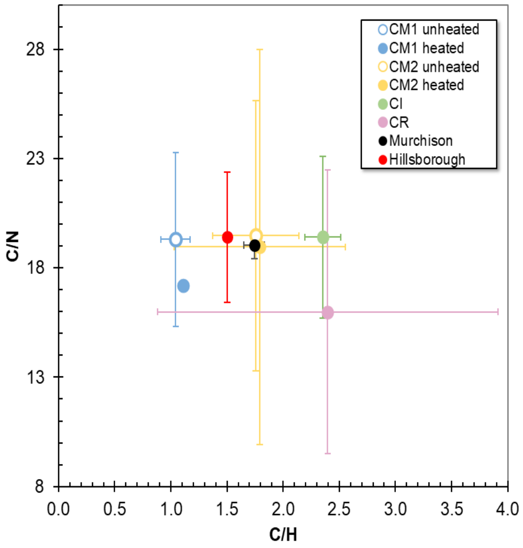

Fig. S-14. C/H versus C/N ratios of bulk Hillsborough (this study) compared to other carbonaceous chondrites (data from *83,7*).

FTIR analysis of individual grains

By: Yoko Kebukawa

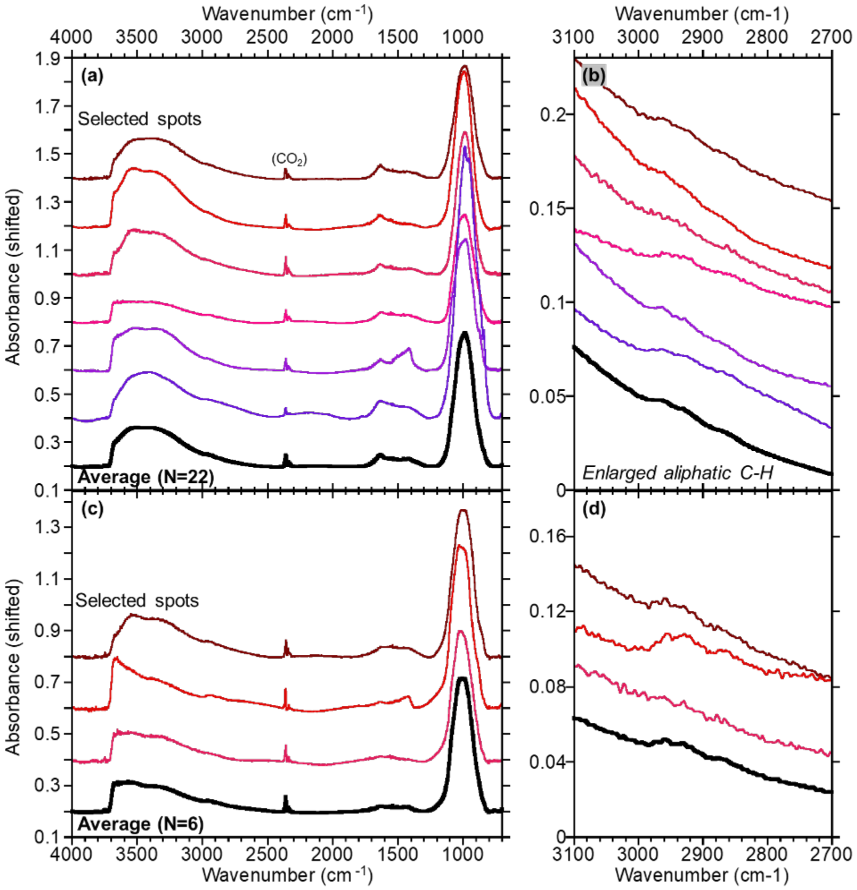

Fig. S-15. Infrared transmission spectra of the Hillsborough meteorite. **(A)** Some selected spectra obtained from 20 µm × 20 µm area and the average spectra (22 spots), and the enlarged aliphatic C-H spectra are shown in **(B)**. **(C)** Spectra (25 µm × 25 µm area) obtained by mild heating (60 °C) in an N_2 flow to remove terrestrial adsorbed water.

The spectra (Fig. S-15) show a large band at ~3400 cm^{-1} assigned to water (adsorbed/interlayer) with a shoulder at 3675 cm^{-1} assigned to structural OH, a strong SiO peak at ~1010 cm^{-1} consistent with phyllosilicates, a band at 1420 cm^{-1} assigned to carbonates, a band at 1630 cm^{-1} assigned to aromatic carbon with adsorbed water, and an aliphatic C-H triplet band at 2960, 2930, and 2860 cm^{-1} assigned to asymmetric stretch of CH_3, asymmetric stretch of CH_2, and symmetric stretch of CH_3+CH_2, respectively. The ~3400 cm^{-1} and 1630 cm^{-1} bands are decreased by mild heating. The shape of the 3400 cm^{-1} band shows some variation due to the heterogeneity of the OH-bearing minerals. There are some carbonate rich spots.

Scanning transmission X-ray microscopy and C-XANES spectroscopy
By: Yoko Kebukawa, Zach Gainsforth, Masanori Suzuki

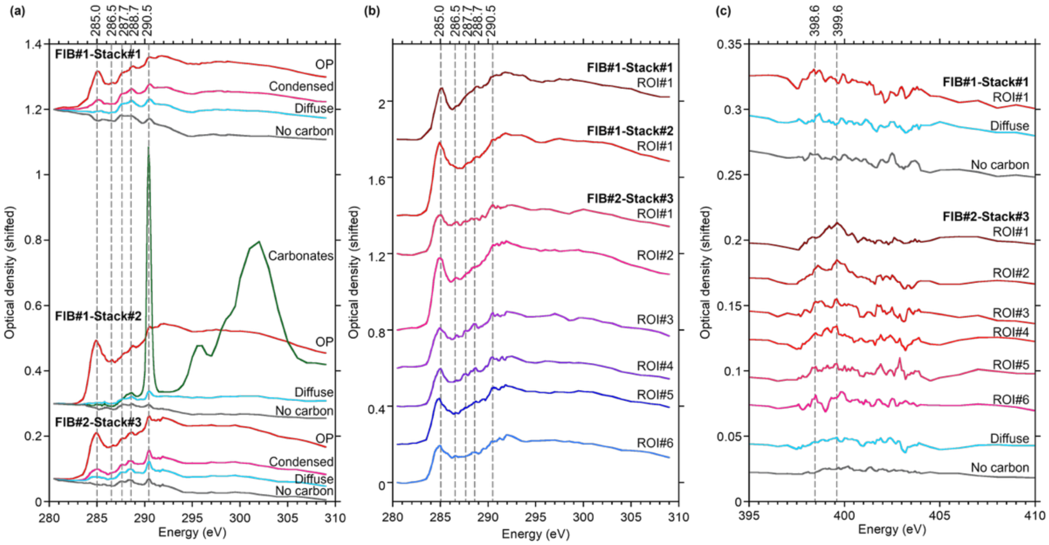

Fig. S-16. C-XANES spectra of the Hillsborough meteorite. **(A)** Average C-XANES spectra from each phase shown in phase images generated by the autoencoder. **(B)** C-XANES spectra from selected regions of interest (ROIs) on organic particles. ROI positions are indicated by numbers in Figs. S-17C–S-17E. **(C)** N-XANES spectra from the ROIs, compared to N-XANES from diffuse and no-carbon regions. C-XANES peak assignments are: 285.0 eV aromatic C, 286.5 eV C=O, 287.7 eV aliphatic C, 288.7 eV C(=O)O, and 290.5 eV organic/inorganic carbonates. N-XANES peak assignments are: 398.6 eV pyridinic N, and 399.6 eV pyridinic N/nitriles.

The average C-XANES spectrum from each phase is shown in Fig. S-16. Fig. S-17 shows the Scanning transmission X-ray microscope (STXM) image at 280 eV of FIB lamellae from the Hillsborough meteorite with the different phases identified in color. These results are discussed in the main text.

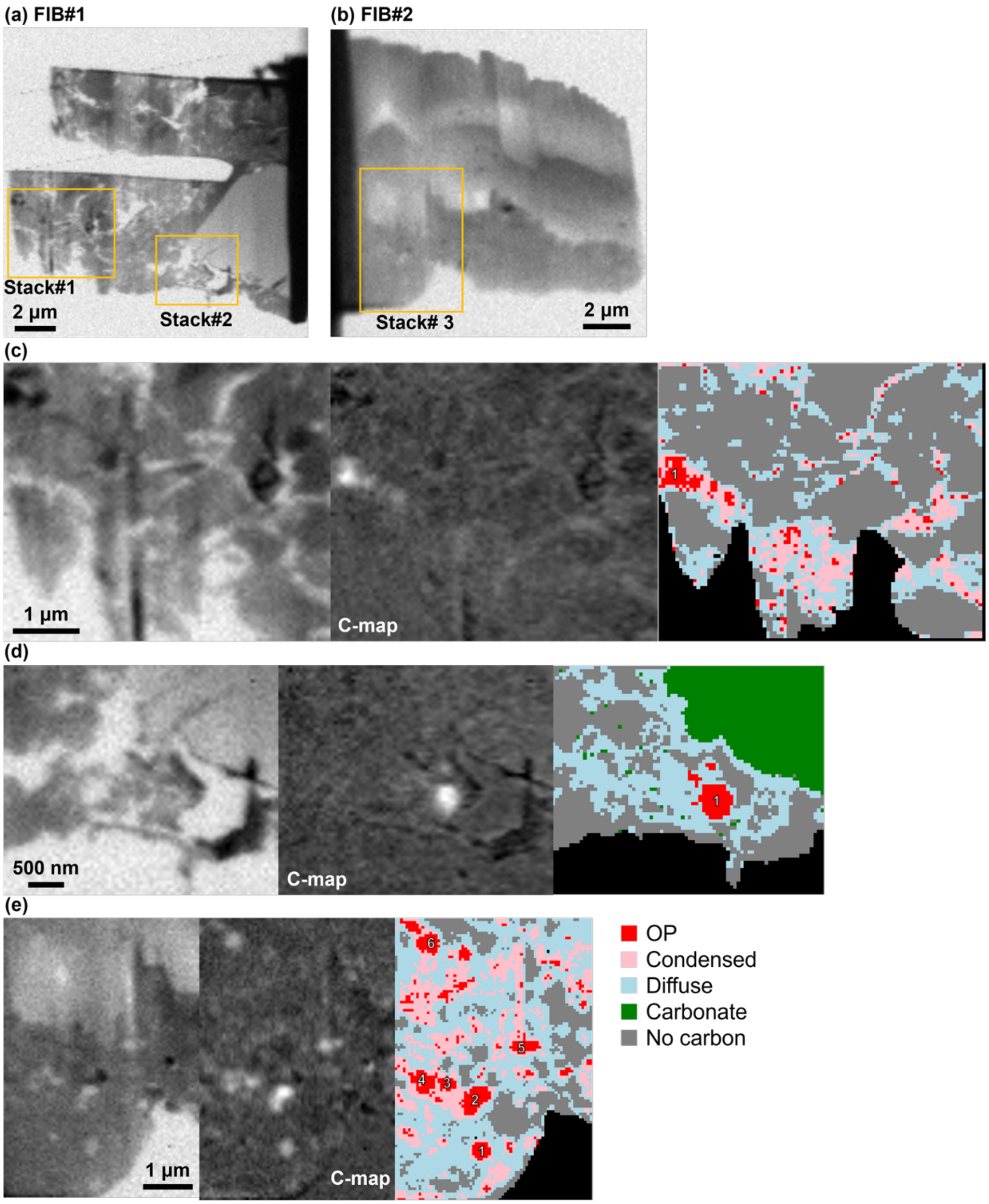

Fig. S-17. (A, B) Scanning transmission X-ray microscope (STXM) image at 280 eV of FIB lamellae from the Hillsborough meteorite. "Stack" images for carbon X-ray absorption near-edge structure (XANES) are obtained from areas indicated by rectangles. **(C–E)** (left panels) Enlarged STXM images at 280 eV, (middle panels) C-map obtained from two images at pre-edge (280 eV) and post-edge (292 eV), and (right panels) phase images generated from autoencoder machine learning of (c) Stack #1, (d) Stack#2, and (e) Stack#3. The average C-XANES spectrum from each phase is shown in Fig. S-16. Red: Organic Particles (OP), pink: condensed organics, light blue: diffuse organics, greed: carbonates, and gray: no/little organics.

Methanol-soluble and insoluble organic compounds
By: Stefan Ruchti, Philippe Schmitt-Kopplin, Jasmine Hertzog, Vincent Carré

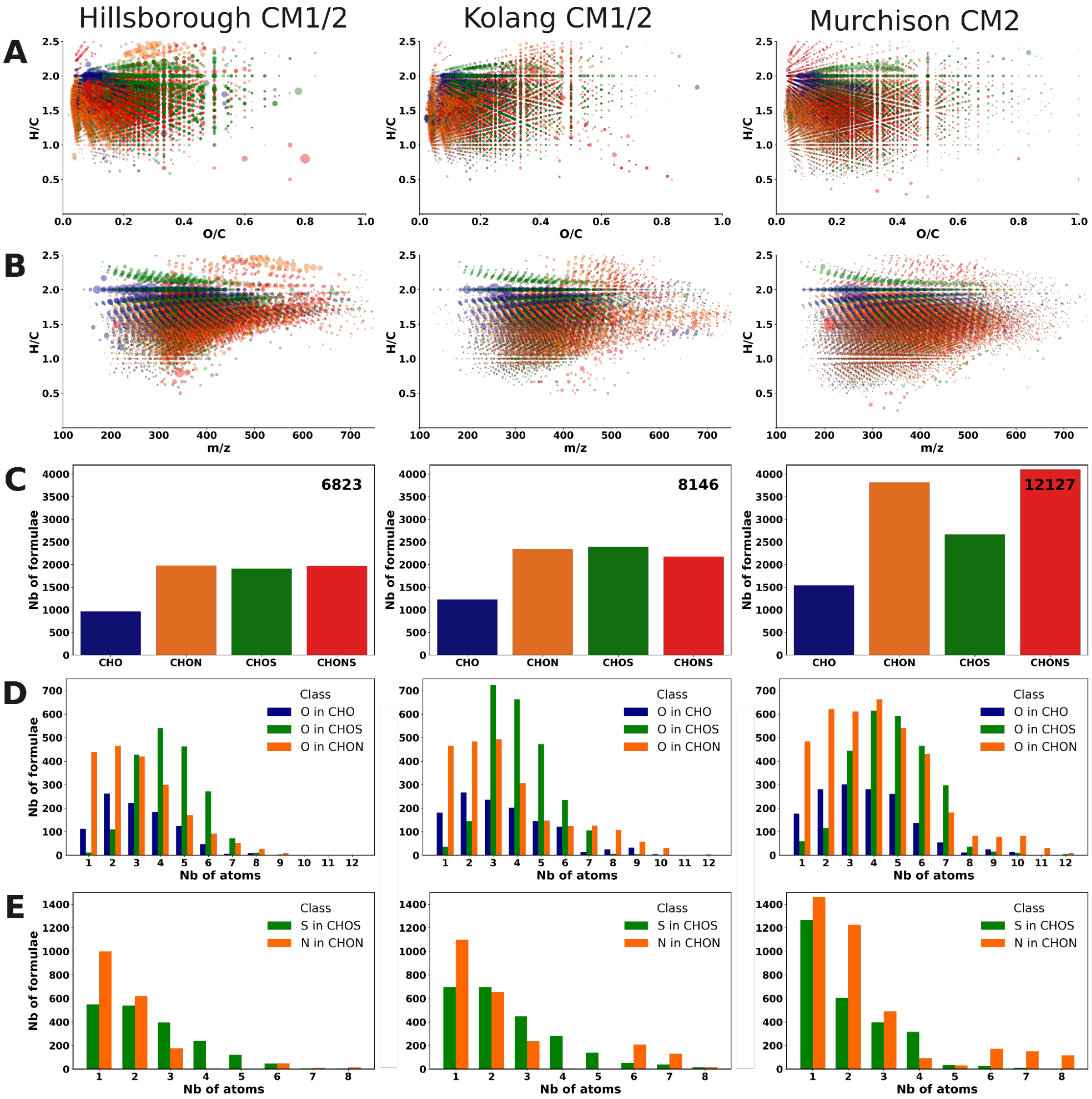

Fig. S-18. Data evaluation of the ESI(-) FT-ICR MS spectra of Hillsborough CM1/2 compared to Kolang CM1/2 and Murchison CM2 showing all CHO, CHNO, CHOS, and CHNOS chemical families with **(A)** classical van Krevelen diagrams **(B)** mass derived van Krevelen, **(C)** distributions of the chemical formula abundances of the various chemical families, **(D)** distribution of the formulas as a function of their abundance in oxygen and **(E)** distribution of the formulas a function of their abundance in nitrogen and sulfur. The bubble size in (A) and (B) is proportional to peak intensity of the mass spectrum and the color legend is reflected in (C).

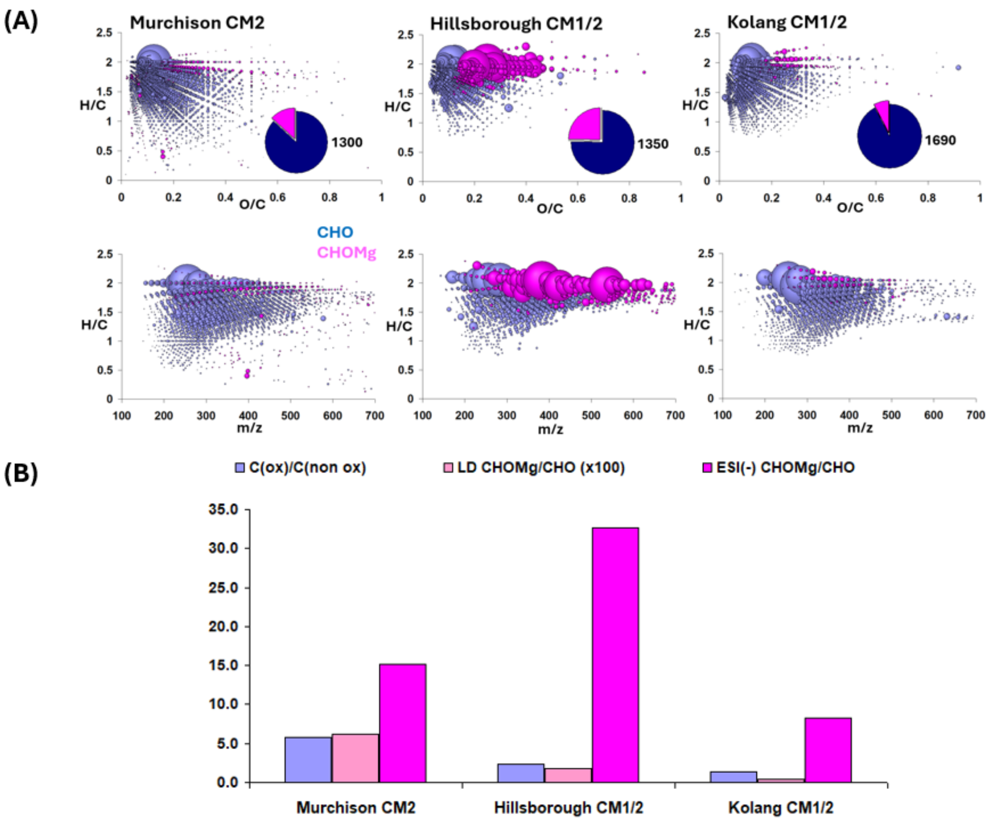

Fig. S-19. (A) Data evaluation from the ESI(-)-FT-ICR MS spectra of Hillsborough CM1/2 compared to Kolang CM1/2 and Murchison CM2 showing all CHO and CHOMg (*84*) chemical families with the classical van Krevelen diagrams and the mass-derived van Krevelen. The bubble size is proportional to peak intensity of the mass spectrum. **(B)** Ratio of oxygenated to non-oxygenated species from LDI(+)-FT-ICR MS in line with the CHOMg/CHO abundance ratios of the three meteorites from LDI(+)-FT-ICR MS and ESI(-)-FT-ICR MS.

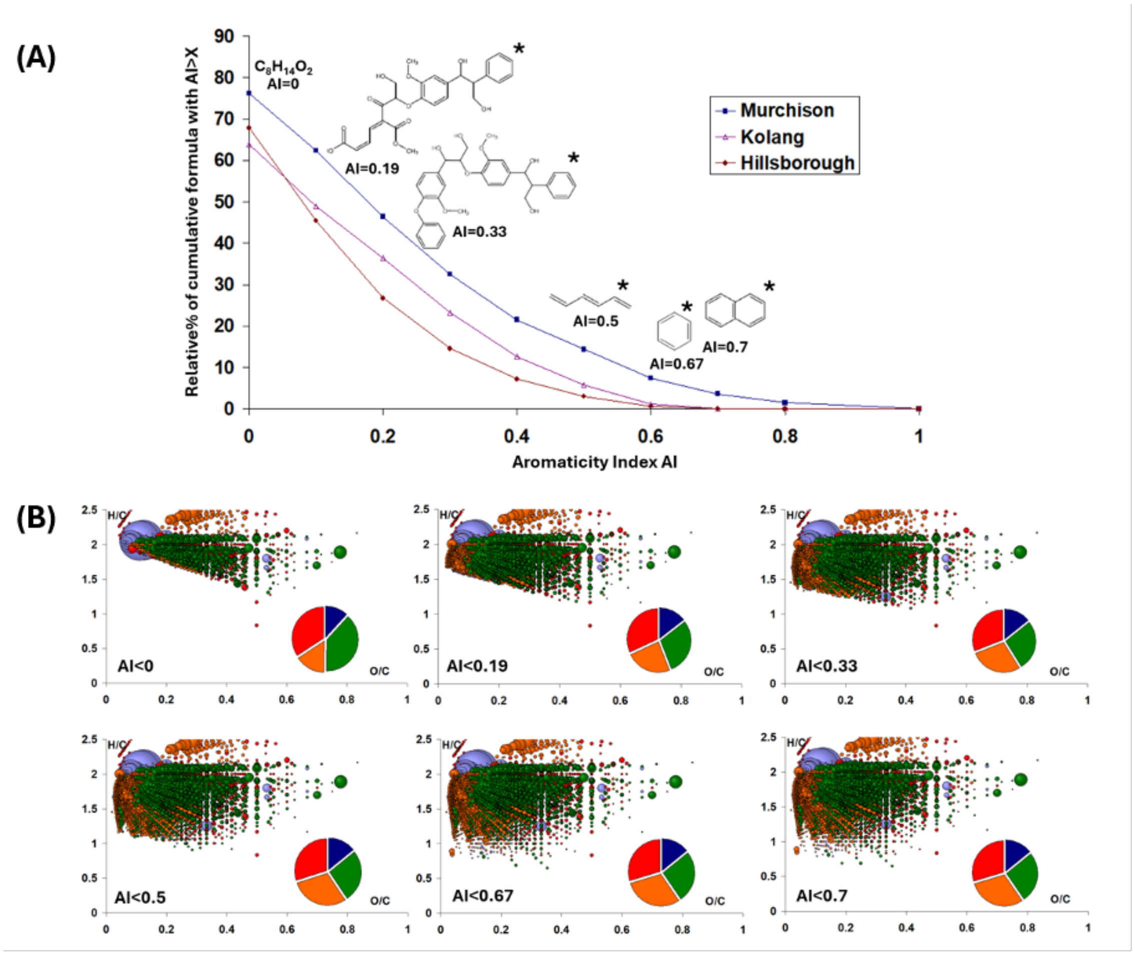

S-20. (A) Aromaticity index (AI) represented as a relative percent of cumulative AI (AI>x). AI was calculated for each annotated chemical formula following the equation: AI = (1+C-O-S-0.5H)/(C-O-S-N-P), where the letters specify the index of an element in the molecular sum formula (*85*). Example chemical structures are shown. **(B)** Visualization of the various regions in the van Krevelen of Hillsborough, corresponding to AI lower than selected values (AI<X; values were selected from the examples in (A)). Pie charts show the count of features assigned to chemical families as in previous figures (Blue: CHO, Orange: CHON, Green: CHOS, Red: CHONS).

Amino acids

By: Hannah L. McLain, Daniel P. Glavin, Jason P. Dworkin

Table S-9. Blank-corrected abundances (nmol/g) of identified 2- to 6-carbon amino acids in the non-hydrolyzed (free) and 6M HCl acid-hydrolyzed (total) hot-water extracts of the Hillsborough and Murchison measured by LC-FD/Q-ToF-MS.

	**Hillsborough (CM1/2)**		**Murchison (CM2)**	
	**Free ($nmol\ g^{-1}$)**	**Total ($nmol\ g^{-1}$)**	**Free ($nmol\ g^{-1}$)**	**Total ($nmol\ g^{-1}$)**
**Acidic amino acids**				
D-Aspartic acid	0.08 ± 0.01	0.360 ± 0.007	0.37 ± 0.01	1.50 ± 0.06
L-Aspartic acid	0.14 ± 0.01	1.60 ± 0.01	0.58 ± 0.01	5.28 ± 0.20
D-Glutamic acid	0.080 ± 0.006	0.40 ± 0.04	0.32 ± 0.02	2.17 ± 0.04
L-Glutamic acid	0.11 ± 0.01	2.45 ± 0.02	0.53 ± 0.01	10.24 ± 0.11
**Hydroxy amino acids**				
D-Serine	0.020 ± 0.001	0.160 ± 0.009	0.140 ± 0.005	0.310 ± 0.003
L-Serine	0.16 ± 0.01	3.22 ± 0.10	0.39 ± 0.02	7.54 ± 0.13
**C2 amino acid**				
Glycine	0.57 ± 0.03	23.25 ± 1.37	3.01 ± 0.10	38.58 ± 0.16
**C3 amino acids**				
β-Alanine	0.11 ± 0.01	0.56 ± 0.02	2.39 ± 0.10	7.11 ± 0.13
D-Alanine	0.30 ± 0.01	1.08 ± 0.03	1.46 ± 0.05	3.70 ± 0.07
L-Alanine	0.32 ± 0.01	5.09 ± 0.22	1.41 ± 0.04	7.21 ± 0.20
**C4 amino acids**				
D,L-α-Amino-*n*-butyric acid	0.18 ± 0.01	0.31 ± 0.02	1.07 ± 0.03	1.55 ± 0.02
D-β-Amino-*n*-butyric acid	0.10 ± 0.01	0.15 ± 0.04	1.35 ± 0.05	2.47 ± 0.06
L-β-Amino-*n*-butyric acid	0.06 ± 0.06	0.22 ± 0.05	1.69 ± 0.13	1.91 ± 0.42
ɣ-Amino-*n*-butyric acid	0.18 ± 0.01	1.40 ± 0.01	1.51 ± 0.05	5.01 ± 0.19
α-Aminoisobutyric acid	0.31 ± 0.01	1.86 ± 0.06	2.60 ± 0.11	12.40 ± 0.42
**C5 amino acids**				
D-Valine	0.08 ± 0.01	0.08 ± 0.01	0.28 ± 0.01	0.500 ± 0.004
L-Valine	0.11 ± 0.02	1.22 ± 0.05	0.20 ± 0.01	4.64 ± 0.07
D,L-Isovaline	0.47 ± 0.04	0.91 ± 0.12	4.67 ± 0.15	9.23 ± 0.12
**C6 amino acid**				
ε-Amino-*n*-caproic acid	1.97 ± 0.11	22.50 ± 1.10	1.82 ± 0.30	1.06 ± 0.01
**Sum C2-C6 amino acids**	**4**	**66**	**23**	**118**

Table S-10. Summary of the D/L ratios and corresponding L-enantiomeric excesses (%Lee = % L - % D) of protein amino acids measured in the Hillsborough and Murchison hot-water extracts. The enantiomeric ratios and Lee values were based on the average of three measurements of each amino acid of the non-hydrolyzed (free) and 6 M HCl-hydrolyzed (total) hot-water extracts after OPA/NAC derivatization and liquid chromatography with UV fluorescence and mass spectrometry detection. The errors shown were calculated by standard error propagation of the uncertainties given for the individual amino acid abundances in Table S-9.

	**Hillsborough (CM1/2)**				**Murchison (CM2)**			
	**Free**		**Total**		**Free**		**Total**	
**Amino Acid**	**D/L**	**Lee (%)**	**D/L**	**Lee (%)**	**D/L**	**Lee (%)**	**D/L**	**Lee (%)**
Aspartic Acid	0.57 ± 0.08	27 ± 5	0.23 ± 0.01	63 ± 1	0.64 ± 0.02	22 ± 1	0.28 ± 0.02	56 ± 2
Glutamic Acid	0.73 ± 0.09	16 ± 5	0.16 ± 0.02	72 ± 2	0.60 ± 0.04	25 ± 3	0.21 ± 0.01	65 ± 1
Serine	0.13 ± 0.01	78 ± 1	0.050 ± 0.003	91 ± 1	0.36 ± 0.02	47 ± 2	0.041 ± 0.001	92 ± 1
Alanine	0.94 ± 0.04	3.2 ± 2.2	0.21 ± 0.01	65 ± 1	1.04 ± 0.05	-1.7 ± 2.3	0.51 ± 0.02	32 ± 1
Valine	0.73 ± 0.16	16 ± 9	0.07 ± 0.01	88 ± 1	1.4 ± 0.1	-17 ± 4	0.11 ± 0.01	81 ± 1

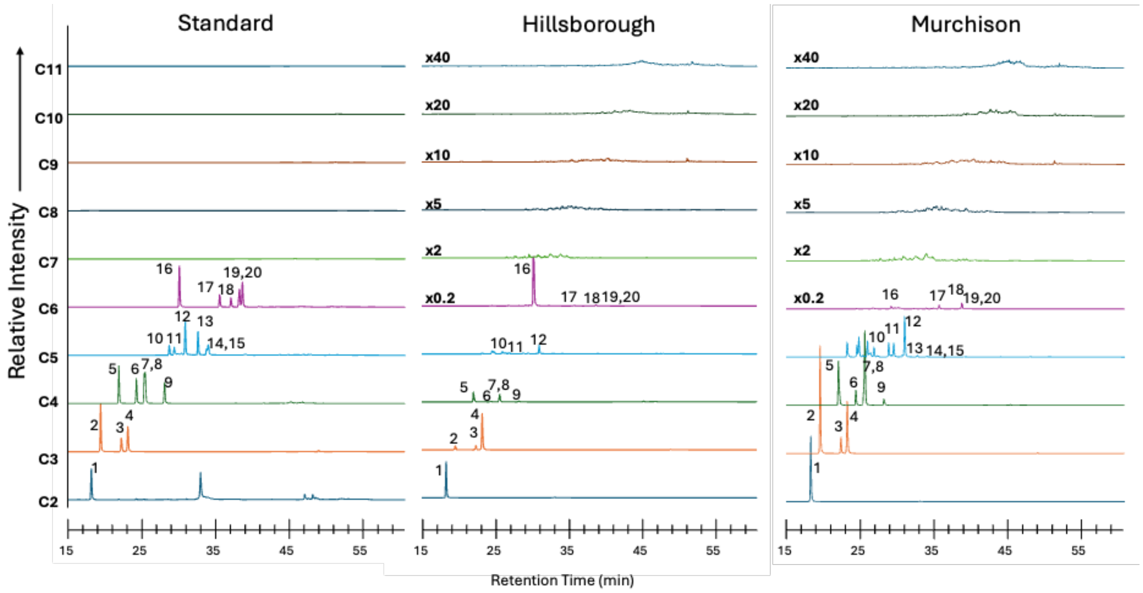

Fig. S-21. LC-ToF-MS chromatograms showing the elution of the C2 to C11 amino acids (H_2N-$(CH_2)_n$-COOH) in the acid-hydrolyzed, hot-water extracts of procedural blank, Hillsborough, and Murchison meteorites, respectively. The 15- to 60-min regions of the LC-ToF single-ion mass chromatograms corresponding to the *o*-phthaldialdehyde/*N*-acetyl-L-cysteine (OPA/NAC) derivatives of C2 to C11 aliphatic primary amino acids in positive ion mode via heated electrospray ionization and a 5 ppm mass accuracy with corresponding *m/z* values as follows: **C2**: *m/z* = 337.08527; **C3**: *m/z* = 351.10092; **C4**: *m/z* = 365.11657; **C5**: *m/z* = 379.13222; **C6**: *m/z* = 393.14787; **C7**: *m/z* = 407.16352; **C8**: *m/z* = 421.17917; **C9**: *m/z* = 435.19482; **C10**: *m/z* = 449.21047; and **C11**: *m/z* = 463.22612. Similar single-ion chromatograms were obtained for the non-hydrolyzed water extracts. Peaks were identified by comparisons of their retention times and exact monoisotopic masses with those in the amino acid standard analyzed on the same day, and are designated by peak number as follows: (1) glycine, (2) β-alanine, (3) D-alanine, (4) L-alanine, (5) γ-aminobutyric acid, (6) D-β-amino-*n*-butyric acid, (7) L-β-amino-*n*-butyric acid, (8) a-aminoisobutyric acid, (9) D,L-a-aminobutyric acid, (10) D-isovaline, (11) L-isovaline, (12) L-valine, (13) D-valine, (14) D-norvaline, (15) L-norvaline, (16) ε-amino-*n*-caproic acid, (17) L-isoleucine, (18) D-isoleucine, (19) D-leucine, and (20) L-leucine.

Table S-11. Detection metrics observed for selected C2 to C6 amino acids using the LC-FD/ToF-MS analytical technique. As a result of derivatization with OPA/NAC, 261 Da is added to the measured mass of each amino acid. Mass error was calculated using the following equation:

	Chemical Formula [M+H]+	SIC RT (min)	FLR RT (min)	Theoretical m/z	Experimental m/z	Mass Error (ppm)
**Acidic amino acids**						
D-aspartic acid	$C_{17}H_{19}N_2O_7S$	4.63	4.48	395.0913	395.0916	0.76
L-aspartic acid	$C_{17}H_{19}N_2O_7S$	4.98	4.85	395.0913	395.0922	2.28
D-glutamic acid	$C_{18}H_{21}N_2O_7S$	7.23	7.10	409.1069	409.1067	-0.49
L-glutamic acid	$C_{18}H_{21}N_2O_7S$	6.93	6.78	409.1069	409.1073	0.98
**Hydroxy amino acids**						
D-serine	$C_{16}H_{19}N_2O_6S$	12.53	12.40	367.0964	367.0961	-0.82
L-serine	$C_{16}H_{19}N_2O_6S$	12.98	12.82	367.0964	367.0969	1.36
**C2 amino acid**						
Glycine	$C_{15}H_{17}N_2O_5S$	18.12	17.98	337.0858	337.0855	-0.89
**C3 amino acids**						
β-alanine	$C_{16}H_{19}N_2O_5S$	19.37	19.40	351.1015	351.1020	1.42
D-alanine	$C_{16}H_{19}N_2O_5S$	22.14	22.00	351.1015	351.1018	0.85
L-alanine	$C_{16}H_{19}N_2O_5S$	23.01	22.87	351.1015	351.1014	-0.28
**C4 amino acids**						
D,L-α-amino-*n*-butyric acid	$C_{17}H_{21}N_2O_5S$	27.91	27.75	365.1171	365.1179	2.19
ɣ-amino-*n*-butyric acid	$C_{17}H_{21}N_2O_5S$	21.88	21.67	365.1171	365.1190	5.20
α-aminoisobutyric acid	$C_{17}H_{21}N_2O_5S$	25.32	25.20	365.1171	365.1177	1.64
**C5 amino acids**						
D-valine	$C_{18}H_{23}N_2O_5S$	32.38	32.22	379.1328	379.1310	-4.75
L-valine	$C_{18}H_{23}N_2O_5S$	30.67	30.52	379.1328	379.1312	-4.22
D-isovaline	$C_{18}H_{23}N_2O_5S$	28.55	28.38	379.1328	379.1322	-1.58
L-isovaline	$C_{18}H_{23}N_2O_5S$	29.21	29.05	379.1328	379.1320	-2.11
**C6 amino acid**						
ε-amino-*n*-caproic acid	$C_{19}H_{25}N_2O_5S$	29.90	29.75	393.1484	393.1491	1.78

The amino acid abundances (Table S-9) and their enantiomeric ratios (Table S-10) in the meteorite extracts and controls were determined by comparison of the peak areas generated from the sample and control UV fluorescence chromatograms (LC-FD, λ_{ex} = 340 nm, λ_{em} = 450 nm) of their OPA/NAC derivatives to the corresponding peak areas of amino acid standards run under the same chromatographic conditions (Fig. S-21) and included peak identification confirmation by accurate mass using a match tolerance of 10 ppm (Table S-11).

Carboxylic acids

By: José Aponte and Denise Buckner

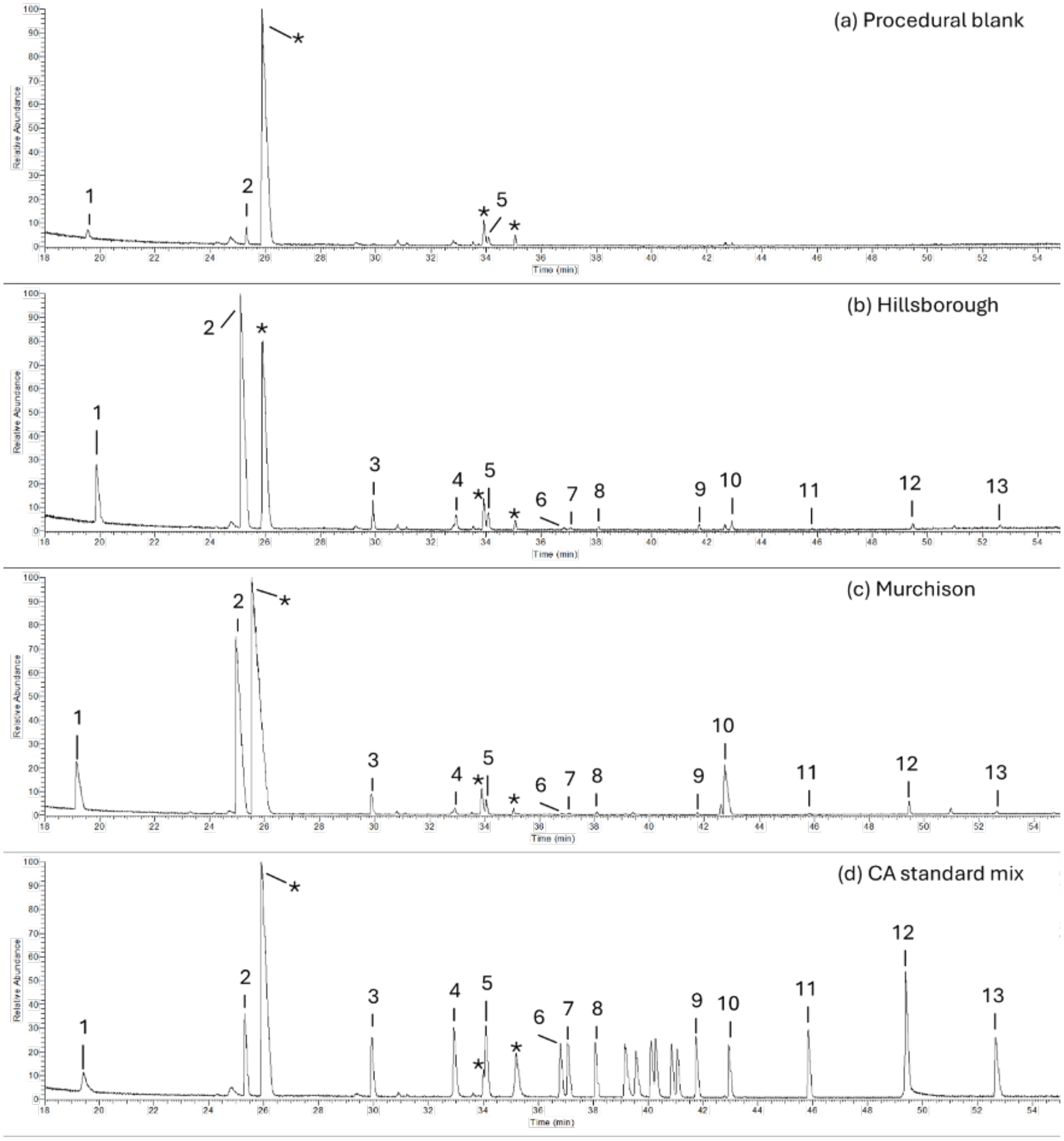

Figure S-22. Chromatograms from GC-MS analyses of carboxylic acids in hot water extracts from (a) procedural blank, (b) Hillsborough, (c) Murchison, and (d) a mix of carboxylic acid (CA) standards. Chromatograms displayed represent summed *m/z* values selected for the carboxylic acids of interest (*m/z* = 42 + 43 + 57 + 71 + 85 + 99 + 101 + 105 + 115). All extracts were derivatized with an *n*-propanol esterification protocol prior to analysis. Compounds include (1) formic, (2) acetic, (3) propanoic, (4) isobutyric, (5) butyric, (6) 2-methylbutyric, (7) isopentanoic, (8) pentanoic, (9) hexanoic, (10) oxalic, (11) benzoic, (12) succinic, and (13) glutaric acids. * indicates *n*-propanol and *n*-propanol derivatization byproducts, and unlabeled peaks in panel (d) correspond to CA standards in the 23-standard mix that were not detected in any of the meteorite samples.

Figure S-22 illustrates the selected ion chromatogram (summed m/z = 42 + 43 + 57 + 71 + 85 + 99 + 101 + 105 + 115) from GC-MS analyses of a procedural blank, the Hillsborough meteorite, the Murchison meteorite, and a mixture of carboxylic acid standards. The summed m/z values were selected for the carboxylic acids of interest. 13 carboxylic acids were detected in both Hillsborough and corresponding Murchison samples, including a suite of straight chain monocarboxylic acids C_1-$C_{6:0}$, three branched carboxylic acids with 4 to 5 carbons, benzoic acid, and three dicarboxylic acids with 2 to 5 carbons (Table S-12). Total carboxylic acids (Table S-13) were 1.78x more abundant in Murchison than Hillsborough, and both meteorites display molecular distributions and abundances consistent with CM chondrites (*68,86*). For both meteorites, acetic acid was the most abundant CA, formic acid was the second most abundant, and abundances of longer-chained MCAs decreased in concentration with increasing molecular weight.

In Hillsborough, isobutyric acid was 2.41x more abundant than butyric acid, but branched C_5 CAs were lower in concentration compared to pentanoic acid. In Murchison, both C_4 and C_5 straight-chain CAs predominated over branched counterparts. Dicarboxylic acids comprised a small fraction of Hillsborough total CAs (4.6%) but made up a larger percentage in Murchison (12.4%), primarily due to a high amount of oxalic acid (Fig. S-22).

Table S-12. Carboxylic acid abundances, in nmol g^{-1}, detected in hot water extracts from the Hillsborough and Murchison meteorites. Extracts were derivatized with *n*-propanol and analyzed with gas chromatography-mass spectrometry (GC-MS). Individual carboxylic values and standard deviations are blank-subtracted and based on the average of triplicate analyses of the same sample. Total carboxylic abundances represent the summed average values of individual compounds, and error was calculated by standard error propagation of the uncertainties for individual carboxylic acid abundances.

Carboxylic Acid	Hillsborough	Murchison
Formic	3,486 ± 409	6,419 ± 460
Acetic	5,774 ± 755	8,508 ± 498
Propanoic	271 ± 50	325 ± 20
Isobutyric	131 ± 19	53 ± 5
Butyric	54 ± 17	102 ± 12
2-Methylbutyric	14 ± 2	16 ± 1
Isopentanoic	10 ± 2	15 ± 1
Pentanoic	7 ± 3	17 ± 9
Hexanoic	132 ± 16	79 ± 2
Oxalic	111 ± 18	2,081 ± 105
Benzoic	10 ±1	17 ± 1
Succinic	16 ± 3	78 ± 6
Glutaric	18 ± 1	22 ± 1
**Total abundance**	**9,919 ± 1,567**	**17,658 ± 1,351**

Table S-13. Compound-specific $\delta^{13}C$ values (‰ VPDB) of carboxylic acids detected in hot water extracts from the Hillsborough and Murchison meteorites. Extracts were derivatized with *n*-propanol and analyzed with isotope ratio mass spectrometry (IRMS). Values and standard deviations for individual $\delta^{13}C$ values were calculated from N = 3 of the same sample.

Carboxylic Acid	Hillsborough	Murchison
Formic	+25.7 ± 12.3	+21.7 ± 7.5
Acetic	-19.1 ± 7.6	-13.9 ± 7.7
Propanoic	n.d.	+6.7 ± 4.7
Oxalic	+7.4 ± 9.7	+44.9 ± 9.8

Table 13 shows the $\delta^{13}C$ values for carboxylic acids that were detectable with IRMS, which includes formic, acetic, and oxalic acids for Hillsborough, and formic, acetic, propanoic, and oxalic acids for Murchison. Carboxylic acid $\delta^{13}C$ values varied across each sample, ranging from -19.1 to +25.7‰ in Hillsborough and -13.9 to +44.9‰ in Murchison values. Formic acid was enriched and displayed similar values for both Hillsborough and Murchison (+25.7 and +21.7‰), acetic acid was depleted for both meteorites (-19.1 and -13.9‰), and oxalic acid displayed positive $\delta^{13}C$ values in both meteorites but is relatively less enriched in Hillsborough (+7.4) compared to Murchison (+44.9).

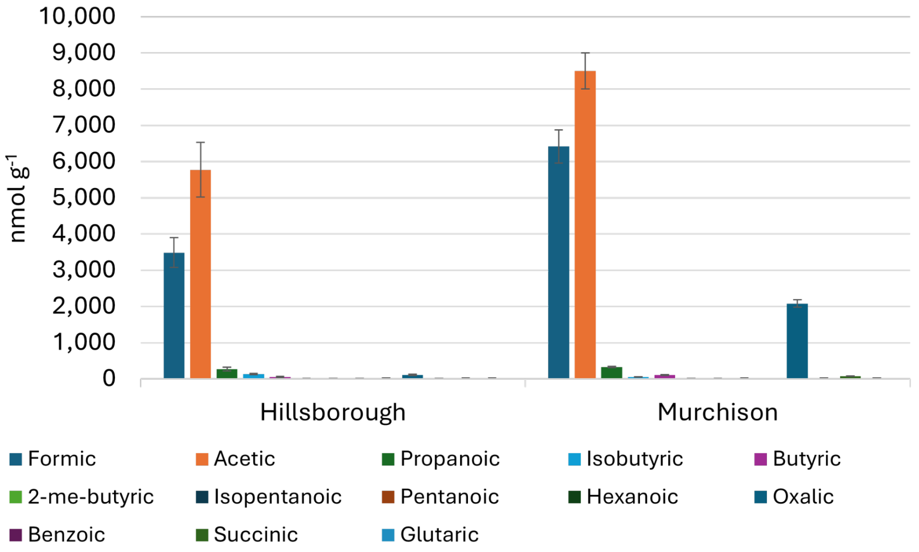

Fig. S-23. Abundances of individual carboxylic acids (nmol g^{-1}) detected in hot water extracts from the Hillsborough and Murchison meteorites. Abundances are blank-subtracted, and error bars represent the standard deviation calculated from triplicate measurements of the same sample. Extracts were derivatized with *n*-propanol. (a) displays all carboxylic acids, and (b) displays lower-abundance compounds only (formic, acetic, and oxalic acids excluded). Compounds include straight-chain and branched monocarboxylic acids (MCAs) from C_1- $C_{6:0}$, benzoic acid, and dicarboxylic acids (DCAs). Straight-chain MCAs: formic (C_1), acetic ($C_{2:0}$), propanoic ($C_{3:0}$), butyric ($C_{4:0}$), pentanoic ($C_{5:0}$), and hexanoic ($C_{6:0}$) acids. Branched

MCAs: isobutyric (C_4), 2-methylbutyric (C_5), and isopentanoic (C_5). DCAs: oxalic (C_2), succinic (C_4), and glutaric (C_5) acids.

Compound-specific stable ^{13}C isotopes (Table S-13) indicate that carboxylic acids in Hillsborough are indigenous, and values similar to Murchison suggest similar isotopic reservoirs in the respective parent bodies or the pre-accretionary cloud. Extraterrestrial carboxylic acids tend to display positive $\delta^{13}C$ values, while CAs in the terrestrial biosphere are generally more ^{13}C-depleted. Formic and oxalic acids in Hillsborough and Murchison were enriched (+25.7 to +44.9‰), consistent with an extraterrestrial origin, while the relatively depleted acetic acid values (-19.1 and -13.9‰) could point to terrestrial contamination (*87,88*). While oxalic acid displays positive isotopic values in both meteorites, oxalic acid in Hillsborough is less enriched than Murchison. Variability in oxalic acid values for the two meteorites could reflect differences in isotopic reservoirs between the two parent bodies or alternatively may indicate contributions from terrestrial contamination for Murchison. Overall, the molecular abundances, distributions, and isotopic signatures for carboxylic acids in Hillsborough (Fig. S-23) point to an extraterrestrial origin and display good agreement with Murchison and other CM1/2 and CM2 chondrites.

Pyrolysis-gas chromatography-triple quadrupole-mass spectrometry
By: Angel Mojarro

Pyrolysis of Hillsborough and Murchison to ~600°C released an identical suite of compounds primarily comprised aromatic hydrocarbons, polycyclic aromatic hydrocarbons (PAHs), and organosulfur compounds along with alkylated species (e.g., alkylbenzenes, alkylnaphthalenes, alkylthiophenes). Aromatic compounds included benzene, naphthalene, phenanthrene, anthracene, fluoranthene, pyrene, C1–C5 alkylbenzenes, and C1–C5 alkylnaphthalenes, C1–C4 alkylphenanthrenes, and C1–C2 alkylpyrenes (Table S-14). Organosulfur compounds included thiophene, benzothiophene, thienothiophenes, benzothiazole, bithiophene, dibenzothiophene, C1–C3 alkylthiophenes, and C1 alkylbenzothiophenes. Nitrogen-containing compounds included pyridine, aniline, benzonitrile, quinoline, indole, and carbazole while oxygen-containing compounds were benzaldehyde, phenol, benzofuran, dibenzofuran, and fluorenone (Fig. S-24).

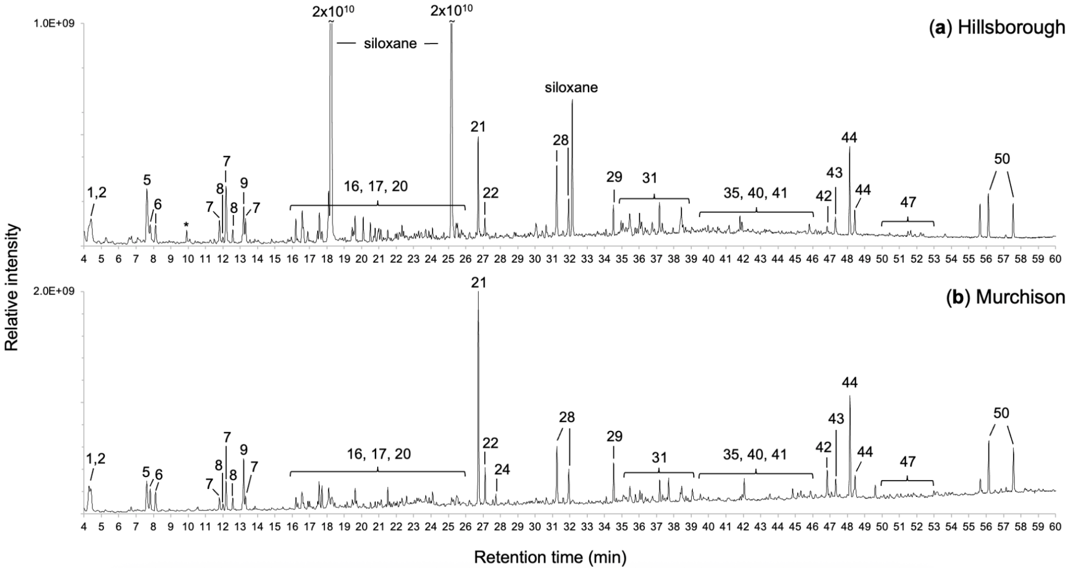

Fig. S-24. Total ion chromatograms (TIC) after flash pyrolysis to 600 °C of the (a) Hillsborough and (b) Murchison meteorites. Analytes were identified by diagnostic mass transitions and retention time. Peaks observable in the TIC are as follows: (1) benzene, (2) thiophene, (5) toluene, (6) C1-alkylthiophenes, (7) C2-alkylbenzenes, (8) C2-alkylthiophenes, (9) styrene, (16) C3-alkylbenzenes, (17) C3-alkylthiophenes, (20) C4-alkylbenzenes, (21) naphthalene, (22) benzothiophene, (28) C1-alkylnaphthalenes, (29) biphenyl, (31) C2-alkylnaphthalenes, (35) C3-alkylnaphthalenes, (40) C4-alkylnaphthalene, (41) C5-alkylnaphthalene, (42) fluorenone, (43) dibenzothiophene, (44) phenanthrene & anthracene, (47) C1-alkylphenanthrenes, (50) fluoranthene & pyrene.

Table S-14. Identified analytes, retention times, and mass transitions for Fused Silica (FS), Murchison (MU), and Hillsborough (HB).

#	Analyte	Time (min)	Precursor m/z	Product m/z	FS	MU	HB
1	Benzene	4.3 ± 1	78.1	52.1	-	+	+
			78.1	63.1	-	+	+
			78.1	77.1	-	+	+
2	Thiophene	4.4 ± 1	84.1	45	-	+	+
			84.1	58	-	+	+
			84.1	69	-	+	+
3	Dimethyl disulfide	6.7 ± 1	94	61	-	+	+
			94	64	-	+	+
			94	79	-	+	+
4	Pyridine	6.8 ± 1.5	52.1	26.1	-	+	+
			79.1	52.1	-	+	+
			79.1	77.1	-	n.d.	n.d.
5	Toluene	7.6 ± 1	91.1	39.1	-	+	+
			91.1	65.1	-	+	+
			92.1	91.1	-	+	+
6	C_1-Alkylthiophenes	8 ± 1.5	97.1	45	-	+	+
			97.1	53.1	-	+	+
			97.1	69	-	+	+
7	C_2-Alkylbenzenes	12.5 ± 3	91.1	65.1	-	+	+
			105.1	77.1	-	+	+
			106.1	91.1	-	+	+
8	C_2-Alkylthiophenes	13 ± 3	111.1	77.1	-	+	+
			112.1	97	-	+	+
			112.1	111	-	+	+
9	Styrene	13.2 ± 1	78.1	52.1	-	+	+
			104.1	78.1	-	+	+
			104.1	103.1	-	+	+
10	Benzaldehyde	16.5 ± 1	77.1	51.1	-	+	+
			105.1	77.1	-	+	+
			106.1	105.1	-	+	+
11	Dimethyl trisulfide	17 ± 1	79	64	-	+	+
			126	79	-	+	+
			126	111.1	-	+	+
12	Aniline	17.4 ± 1	93.1	65.1	-	+	+
			93.1	66.1	-	+	+
			93.1	92.1	-	+	+
13	Phenol	17.6 ± 1	66.1	65.1	-	+	+
			94.1	39.1	-	+	+
			94.1	66.1	-	+	+
14	Benzonitrile	17.7 ± 1	76.1	50	-	+	+
			103.1	50	-	+	+
			103.1	76.1	-	+	+
15	Benzofuran	18.3 ± 1	90.1	63.1	-	+	+
			90.1	89.1	-	+	+
			118.1	117.1	-	+	+
16	C_3-Alkylbenzenes	18.5 ± 6	105.1	77.1	-	+	+
			105.1	103.1	-	+	+
			120.2	105.1	-	+	+

#	Analyte	Time (min)	Precursor m/z	Product m/z	FS	MU	HB
17	C_3-Alkylthiophenes	19 ± 8	125.1	97.1	-	+	+
			126.1	111	-	+	+
			126.1	125.1	-	+	+
18	Indane	19.6 ± 1	115.1	89.1	-	+	+
			117.1	115.1	-	+	+
			118.1	117.1	-	+	+
19	Indene	20.5 ± 1	115.1	89	-	+	+
			115.1	114.2	-	+	+
			116.1	115.1	-	+	+
20	C_4-Alkylbenzenes	23 ± 8	119.1	77.1	-	+	+
			119.1	91.1	-	+	+
			134.2	119.1	-	+	+
21	Naphthalene	26.8 ± 1	128.1	78.1	-	+	+
			128.1	102.1	-	+	+
			128.1	127.1	-	+	+
22	Benzo[c]thiophene	27.1 ± 1	134.1	89.1	-	+	+
			134.1	90.1	-	+	+
			134.1	108	-	+	+
23	Benzothiazole	28.5 ± 1	108	69	-	+	+
			135	91	-	+	+
			135	108	-	+	+
24	Thieno[n,n]thiophenes	28.5 ± 2	96.1	70	-	+	+
			96.1	95	-	+	+
			140	96	-	+	+
25	Quinoline	29 ± 1	129.1	102.1	-	+	+
			129.1	128.1	-	+	+
			102.1	102.1	-	+	+
26	Indole	31.2 ± 1.5	89	63	-	+	+
			90	89.1	-	+	+
			117	89.1	-	+	+
27	C_1-Alkylbenzo[b]thiophenes	31.5 ± 2	147.1	77.1	-	+	+
			147.1	103.1	-	+	+
			148.1	147	-	+	+
28	C_1-Alkylnaphthalenes	31.6 ± 1.5	141.1	115.1	-	+	+
			142.1	115.1	-	+	+
			142.1	141.1	-	+	+
29	Biphenyl	34.5 ± 1	76.1	63.1	-	+	+
			154.1	152.1	-	+	+
			154.1	153.1	-	+	+
30	Bithiophene	36 ± 3	121	77	-	+	+
			166	121	-	+	+
			166	134	-	+	+
31	C_2-Alkylnaphthalenes	36.1 ± 4	141.1	115.1	-	+	+
			156.1	115.1	-	+	+
			156.1	141.1	-	+	+
32	Acenaphthylene	37.2 ± 1	76.1	63.1	-	+	+
			152.1	126.1	-	+	+
			152.1	151.1	-	+	+
33	Acenaphthene	38.5 ± 1	153.1	152.1	-	+	+
			154.1	153.1	-	+	+

#	Analyte	Time (min)	Precursor m/z	Product m/z	FS	MU	HB
34	Dibenzofuran	39.5 ± 1	139.1	113	-	+	+
			168.1	139	-	+	+
			169.1	140	-	+	+
35	C_3-Alkylnaphthalenes	40.5 ± 5.5	155.2	128.1	-	+	+
			155.2	153.1	-	+	+
			170.2	155.1	-	+	+
36	Phenalene	41.2 ± 1.5	165.1	115	-	+	+
			165.1	139	-	+	+
			166.1	165.1	-	+	+
37	Fluorene	41.8 ± 1.5	165.1	95.1	-	+	+
			165.1	109.1	-	+	+
			166.1	165.1	-	+	+
39	Benzophenone	43.4 ± 1	105	77.1	-	+	+
			182.1	105	-	+	+
			182.1	181.1	-	+	+
40	C_4-Alkylnaphthalene	44 ± 6	169.1	153.1	-	+	+
			184.1	154.1	-	+	+
			184.1	169.1	-	+	+
41	C_5-Alkylnaphthalene	46 ± 7.5	183.1	153.1	-	-	-
			183.1	168.1	-	-	-
			198.1	183.2	-	-	-
42	Fluorenone	46.8 ± 1	152.1	126	-	+	+
			152.1	151.1	-	+	+
			180.1	152.1	-	+	+
43	Dibenzothiophene	47.3 ± 2	139.1	113	-	+	+
			184.1	139.1	-	+	+
			184.1	152.1	-	+	+
44	Phenanthrene + Anthracene	48.3 ± 2.5	178.1	152.1	-	+	+
			178.1	176.1	-	+	+
			178.1	177.1	-	+	+
46	Carbazole	49.8 ± 1	167.1	139.1	-	+	+
			167.1	140.1	-	+	+
			167.1	166.1	-	+	+
47	C_1-Alkylphenanthrenes	52 ± 4	191.1	165.1	-	+	+
			192.1	165.1	-	+	+
			192.1	191.1	-	+	+
48	Phenylnaphtalene	53.7 ± 1.5	204.1	189.1	-	+	+
			204.1	202.1	-	+	+
			204.1	203.1	-	+	+
49	C_2-Alkylphenanthrenes	55.2 ± 4	206.1	190.1	-	+	+
			206.1	191.1	-	+	+
			206.1	205.1	-	+	+
50	Fluoranthene + Pyrene	56.8 ± 2.5	202.1	200.1	-	+	+
			202.1	201.1	-	+	+
			101.1	100.1	-	+	+
51	C_3-Alkylphenanthrenes	58.5 ± 6	220.1	189.1	-	+	+
			220.1	190.1	-	+	+
			220.1	219.1	-	+	+
52	C_1-Alkylpyrenes/fluoranthenes	60 ± 3	216.1	215.1	-	+	+
			216.1	189.1	-	+	+
			216.1	214.1	-	+	+

#	Analyte	Time (min)	Precursor m/z	Product m/z	FS	MU	HB
53	C_2-Alkylpyrenes	60 ± 4	215.1	213.1	-	+	+
			230.1	215.1	-	+	+
			230.1	229.1	-	+	+
54	C_4-Alkylphenanthrenes	61 ± 5	219.1	189.1	-	-	-
			219.1	204.1	-	-	-
			234.1	219.1	-	-	-
55	C_3-Alkylpyrenes	65 ± 6	244.1	215.1	-	-	-
			244.1	228.1	-	-	-
			244.1	229.1	-	-	-
56	Terphenyl series	65 ± 5	230.1	215.1	-	+	+
			230.1	228	-	+	+
			230.1	229.1	-	+	+
57	Triphenylene/Chrysene /Naphthacene	65.1 ± 3	228.1	202.1	-	+	+
			228.1	226.1	-	+	+
			228.1	227.1	-	+	+

Fig. S-25. Polycyclic aromatic hydrocarbons (PAHs) and siloxane contaminants (marked "*") measured in the Hillsborough meteorite by pyrolysis gas chromatography mass spectrometry (PyGC-MS).

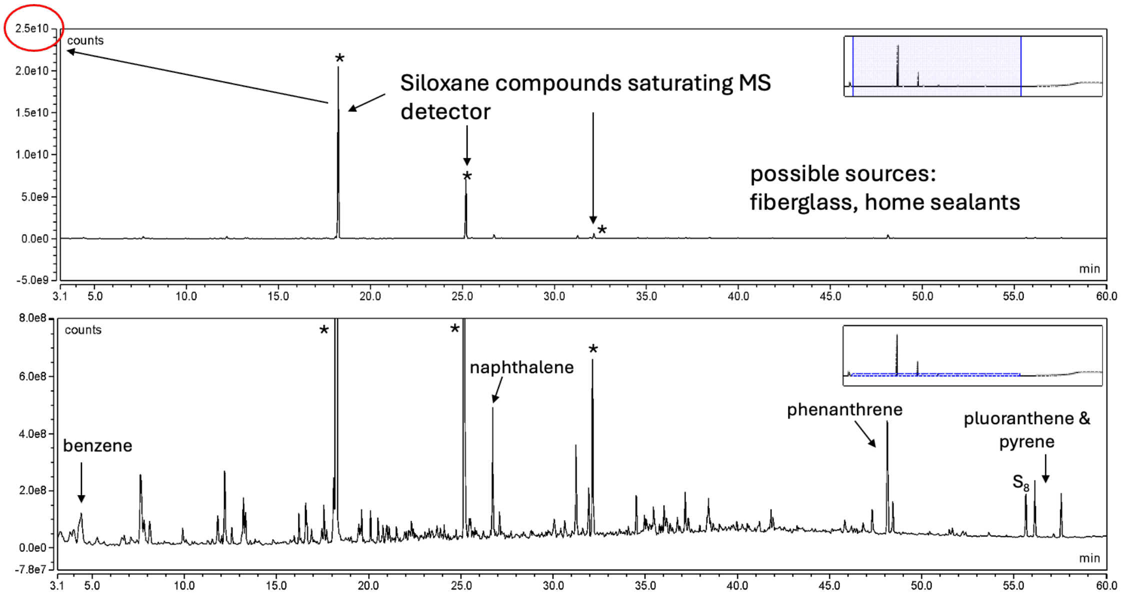

Fig. S-26. Comparison of the distributions of hydrocarbons measured in the Hillsborough and Murchison meteorites by PyGC-MS.

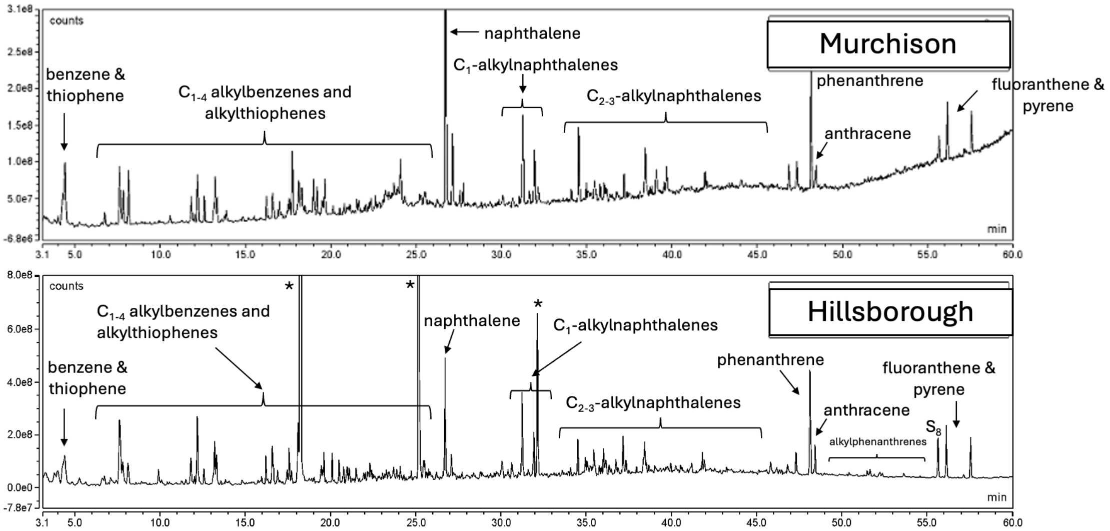

Fig. S-27. Comparison of the distributions of hydrocarbons measured in the Winchcombe and Hillsborough meteorites by PyGC-MS.

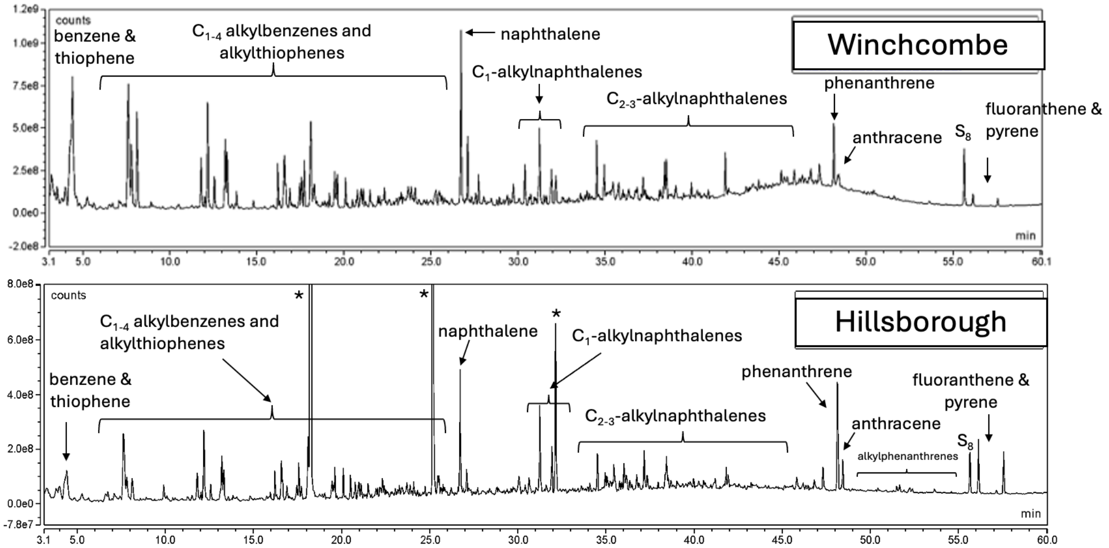

Amino acid content by GC-MS analysis

By: Queenie H. S. Chan, Diptimayee Behera, Jonathan S. Watson, Mark A. Sephton

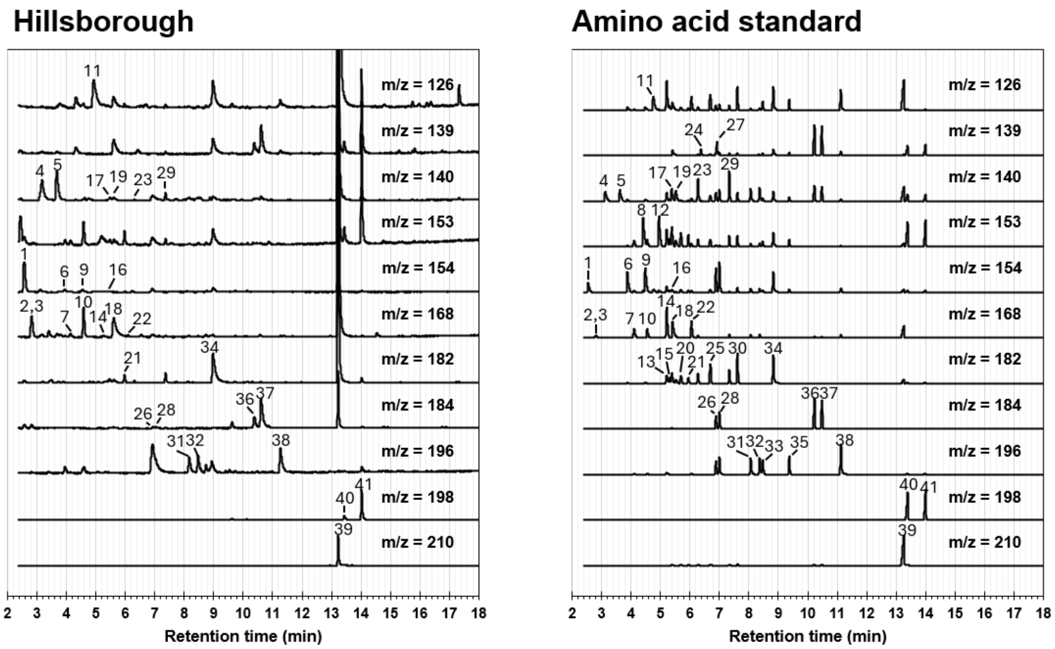

Fig. S-28. The 2–18 min regions of the GC-MS chromatograms. Single ion GC-MS traces (m/z 126, 139, 140, 153, 154, 168, 182, 184, 196, 198, and 210) of the derivatized (N-TFA, O-isopropyl) 6 N HCl-hydrolyzed hot-water extracts of the Hillsborough meteorite and the mixed amino acid standard. The peaks were identified by comparing the retention time and mass fragmentation pattern to those in the amino acid standard run on the same day. D- and L-isovaline enantiomers could not be separated under the chromatographic conditions (Peaks #2 and #3) but their separation was achieved on a different column (CP-Chirasil-Dex CB GC Column). The amino acids are designated by peak number as follows: (1) α-aminoisobutyric acid, (2) D-isovaline, (3) L-isovaline, (4) D-alanine, (5) L-alanine, (6) D-α-amino-n-butyric acid, (7) D-valine, (8) D-threonine, (9) L-α-amino-n-butyric acid, (10) L-valine, (11) Glycine, (12) L-threonine, (13) D-β-aminoisobutyric acid, (14) D-norvaline, (15) L-β-aminoisobutyric acid, (16) D-isoleucine, (17) D-β-amino-n-butyric acidb, (18) β-alanine, (19) L-β-amino-n-butyric acidb, (20) L-alloisoleucine, (21) L-isoleucine, (22) L-norvaline, (23) D-leucine, (24) D-serine, (25) D-norleucine, (26) R-3-aminopentanoic acid, (27) L-serine, (28) S-3-aminopentanoic acid, (29) L-leucine, (30) L-norleucine, (31) D-4-amino-pentanoic acid, (32) L-4-amino-pentanoic acid, (33) D-2-aminoheptanoic acid, (34) γ-amino-n-butyric acid, (35) L-2-aminoheptanoic acid, (36) D-aspartic acid, (37) L-aspartic acid, (38) 5-aminopentanoic acid, (39) ε-amino-n-caproic acid, (40) D-glutamic acid, and (41) L-glutamic acid.

Table S-15. Blank-corrected abundances (nmol/g) of identified two- to six-carbon amino acids in the non-hydrolyzed (free) and 6M HCl acid-hydrolyzed (total) hot-water extracts of the Hillsborough and Murchison meteorites measured by GC-MS.

	Hillsborough (CM1/2)		Murchison (CM2)	
	**Free ($nmol\ g^{-1}$)**	**Total ($nmol\ g^{-1}$)**	**Free ($nmol\ g^{-1}$)**	**Total ($nmol\ g^{-1}$)**
**Acidic amino acids**				
D-Aspartic acid	0.02 ± 0.01	3.71 ± 3.23	0.99 ± 0.35	1.84 ± 0.72
L-Aspartic acid	0.03 ± 0.00	11.52 ± 10.07	5.07 ± 1.84	6.71 ± 2.95
D-Glutamic acid	n.f.	6.54 ± 2.85	0.50 ± 0.20	3.07 ± 1.10
L-Glutamic acid	n.f.	38.63 ± 14.42	1.92 ± 0.72	17.31 ± 4.39
**Hydroxy amino acids**				
D-Serine	n.f.	n.f.	1.60 ± 1.08	0.88 ± 0.41
L-Serine	n.f.	n.f.	2.18 ± 1.69	n.f.
**C2 amino acid**				
Glycine	3.28 ± 0.85	101.03 ± 34.42	24.40 ± 3.90	20.23 ± 10.94
**C3 amino acids**				
β-Alanine	0.90 ± 0.21	34.34 ± 13.06	7.89 ± 1.90	9.42 ± 4.67
D-Alanine	1.55 ± 0.35	62.93 ± 26.20	10.89 ± 2.38	15.47 ± 9.05
L-Alanine	1.42 ± 0.35	68.46 ± 24.43	11.30 ± 1.59	16.97 ± 11.47
**C4 amino acids**				
D,L- α -Amino-*n*-butyric acid	0.06 ± 0.01	23.34 ± 7.77	2.74 ± 0.71	5.38 ± 2.57
D-β-Amino-*n*-butyric acid	0.10 ± 0.06	7.17 ± 3.29	1.03 ± 0.57	1.10 ± 0.71
L-β-Amino-*n*-butyric acid	0.09 ± 0.10	8.58 ± 3.95	0.85 ± 0.51	1.65 ± 0.89
ɣ-Amino-*n*-butyric acid	0.03 ± 0.04	26.05 ± 10.89	3.29 ± 1.14	11.28 ± 4.77
α-Aminoisobutyric acid	3.25 ± 0.58	78.62 ± 31.71	38.83 ± 6.86	18.39 ± 11.83
**C5 amino acids**				
D-Valine	n.f.	9.66 ± 4.63	0.36 ± 0.35	1.72 ± 1.05
L-Valine	n.f.	61.69 ± 22.33	5.63 ± 0.60	15.68 ± 9.55
D,L-Isovaline	4.25 ± 0.74	82.70 ± 40.24	51.87 ± 8.15	19.77 ± 12.89
**C6 amino acid**				
ε-Amino-*n*-caproic acid	n.f.	207.96 ± 100.04	31.61 ± 8.58	141.77 ± 37.32
**Sum C2-C6 amino acids**	**14.98**	**624.97**	**171.34**	**166.87**

Table S-16. The D/L ratios and corresponding L-enantiomeric excesses (% Lee = % L − % D) of amino acids measured in the Hillsborough and Murchison hot-water extracts. The errors shown were calculated by standard error propagation of the uncertainties given for the individual amino acid abundances.

	Hillsborough (CM1/2)				Murchison (CM2)			
	**Free**		**Total**		**Free**		**Total**	
**Amino Acid**	**D/L**	**Lee (%)**	**D/L**	**Lee (%)**	**D/L**	**Lee (%)**	**D/L**	**Lee (%)**
Aspartic Acid	0.71 ± 0.31	17.2 ± 15.2	0.32 ± 1.23	51.3 ± 45.5	0.19 ± 0.51	67.4 ± 13.8	0.28 ± 0.59	56.9 ± 19.8
Glutamic Acid	n.r.	n.r.	0.17 ± 0.57	71.0 ± 14.2	0.26 ± 0.55	58.8 ± 18.1	0.18 ± 0.44	69.9 ± 11.2
Serine	n.r.	n.r.	n.r.	n.r.	n.r.	15.2 ± 50.2	n.r.	n.r.
Alanine	1.09 ± 0.34	-4.1 ± 16.8	0.92 ± 0.55	4.2 ± 27.4	0.96 ± 0.26	1.8 ± 13.0	0.91 ± 0.89	4.6 ± 44.6
Valine	n.r.	n.r.	0.08 ± 0.33	72.9 ± 14.1	0.06 ± 0.33	87.9 ± 3.7	0.11 ± 0.86	80.3 ± 15.3
Isovaline	0.96 ± 0.25	2.2 ± 12.3	1.02 ± 0.69	-0.9 ± 34.4	0.86 ± 0.22	7.3 ± 11.1	1.02 ± 0.92	-1.2 ± 46.1

Fig. S-28 shows the 2–18 min regions of the GC-MS chromatograms of the Hillsborough meteorite and the mixed amino acid standard. Blank-corrected abundances (nmol/g) of identified two- to six-carbon amino acids in the non-hydrolyzed (free) and 6M HCl acid-hydrolyzed (total) hot-water extracts of the Hillsborough and Murchison meteorites measured by GC-MS are given in Table S-15. The D/L ratios and corresponding L-enantiomeric excesses (% Lee = % L − % D) of amino acids measured in the Hillsborough and Murchison hot-water extracts are given in Table S-16. Results are discussed in the main text.

REFERENCES

1. S. De Angelis, C. Carli, F. Tosi, P. Beck, O. Brissaud, B. Schmitt, S. Potin, M. C. De Sanctis, F. Capaccioni, G. Piccioni, NIR reflectance spectroscopy of hydrated and anhydrous sodium carbonates at different temperatures. *Icarus* **317**, 388–411 (2019).

2. N. Stein, B. L. Ehlmann, D. J. Stevenson, J. Castillo-Rogez, C. A. Raymond, Bright Na-carbonate exposures reveal recent, widespread mobilization of material in Ceres' shallow subsurface. *J. Geophys. Res.* **128**, e2023JE007868 (2023).

3. M. J. Poston, S. R. Baker, J. E. C. Scully, E. M. Carey, L. E. Mc Keown, J. C. Castillo-Rogez, C. A. Raymond, Experimental examination of brine and water lifetimes after impact on airless worlds. *Planet. Sci. J.* **5**, 40 (2024).

4. I. F. Pamerleau, M. M. Sori, J. E. C. Scully, An ancient and impure frozen ocean on Ceres implied by its ice-rich crust. *Nat. Astron.* **8**, 1373–1379 (2024).

5. T. Matsumoto, T. Noguchi, A. Miyzek, Y. Igami, M. Matsumoto, T. Yada, M. Uesugi, M. Yasutake, K. Uesugi, A. Takeuchi, H. Yuzawa, T. Ohigashi, T. Araki, Sodium carbonates on Ryugu as evidence of highly saline water in the outer Solar System. *Nat. Astron.* **8**, 1536–1543 (2024).

6. T. J. McCoy, S. S. Russell, T. J. Zega, K. L. Thomas-Keprta, S. A. Singerling, F. E. Brenker, N. E. Timms, W. D. A. Rickard, J. J. Barnes, G. Libourel, S. Ray, C. M. Corrigan, P. Haenecour, Z. Gainsforth, G. Dominguez, A. J. King, L. P. Keller, M. S. Thompson, S. A. Sandford, R. H. Jones, An evaporite sequence from ancient brine recorded in Bennu samples. *Nature* **637**, 1072–1077 (2025).

7. C. M. O'D. Alexander, R. Bowden, M. Fogel, K. Howard, C. Herd, L. Nittler, The provenances of asteroids, and their contributions to the volatile inventories of the terrestrial planets. *Science* **337**, 721–723 (2012).

8. R. C. Greenwood, I. A. Franchi, R. Findlay, J. A. Malley, M. Ito, A. Yamaguchi, M. Kimura, N. Tomioka, M. Uesugi, N. Imae, N. Shirai, T. Ohigashi, M. C. Liu, K. A. McCain, N. Matsuda, K. D. McKeegan, K. Uesugi, A. Nakato, K. Yogata, H. Yuzawa, Y. Kodama, A.

Tsuchiyama, M. Yasutake, K. Hirahara, A. Tekeuchi, S. Sekimoto, I. Sakurai, I. Okada, Y. Karouji, S. Nakazawa, T. Okada, T. Saiki, S. Tanaka, F. Terui, M. Yoshikawa, A. Miyazaki, M. Nishimura, T. Yada, M. Abe, T. Usui, S. Watanabe, Y. Tsuda, Oxygen isotope evidence from Ryugu samples for early water delivery to Earth by CI chondrites. *Nat. Astron.* **7**, 29–38 (2023).

9. A. J. King, P. F. Schofield, S. S. Russell, Type 1 aqueous alteration in CM carbonaceous chondrites: Implications for the evolution of water-rich asteroids. *Meteorit. Planet. Sci.* **52**, 1197–1215 (2017).

10. Meteoritical Bulletin (2026). https://www.lpi.usra.edu/meteor/metbull.cfm?code=87290.

11. E. A. Silber, D. C. Bowman, C. G. Carr, D. P. Eisenberg, B. R. Elbing, B. Fernando, M. A. Garcés, R. Haaser, S. Krishnamoorthy, C. A. Langston, Y. Nishikawa, J. Webster, J. F. Anderson, S. Arrowsmith, S. Bazargan, L. Beardslee, B. Beck, J. W. Bishop, P. Blom, G. Bracht, D. L. Chichester, A. Christe, J. Clarke, K. Cummins, J. Cutts, L. Danielson, C. Donahue, K. Eack, M. Fleigle, D. Fox, A. Goel, D. Green, Y. Hasumi, C. Hayward, D. Hicks, J. Hix, S. Horton, E. Hough, D. P. Huber, M. A. Hunt, J. Inman, S. M. A. Islam, J. Izraelevitz, J. D. Jacob, J. Johnson, R. J. Kc, A. Komjathy, E. Lam, J. LaPierre, K. Lewis, R. D. Lewis, P. Liu, L. Martire, M. McCleary, E. A. McGhee, I. Mitra, A. Nag, L. O. Giraldo, K. Pearson, M. Plaisir, S. K. Popenhagen, H. Rassoul, M. R. Giannone, M. Samnani, N. Schmerr, K. Spillman, G. Srinivas, S. K. Takazawa, A. Tempert, R. Turley, C. Van Beek, L. Viens, O. A. Walsh, N. Weinstein, R. White, B. Williams, T. C. Wilson, S. Wyckoff, M. Yamamoto, Z. Yap, T. Yoshiyama, C. Zeiler, Geophysical observations of the 2023 September 24 OSIRIS-REx sample return capsule reentry. *Planet. Sci. J.* **5**, 47 (2024).

12. E. A. Silber, J. Trigo-Rodriguez, I. Oseghae, E. Peña Asensio, M. B. Boslough, R. Whitaker, C. Pilger, P. Lubin, V. Sawal, C. Hetzer, R. Longenbaugh, P. Jenniskens, B. Bailey, E. Mas Sanz, P. Hupe, A. N. Cohen, T. R. Edwards, S. Egan, R. E. Silber, S. Czarnowski, M. Ronac Giannone, Multi-parameter constraints on empirical infrasound period–yield relations for bolides and implications for planetary defense. *Astron. J.* **170**, 38 (2025).

13. N. Braukmüller, F. Wombacher, D. C. Hezel, R. Escoube, C. Münker, The chemical composition of carbonaceous chondrites: Implications for volatile element depletion, complementarity and alteration. *Geochim. Cosmochim. Acta* **239**, 17–48 (2018).

14. R. J. Macke, G. J. Consolmagno, D. T. Britt, Density, porosity, and magnetic susceptibility of carbonaceous chondrites. *Meteorit. Planet. Sci.* **46**, 1842–1862 (2011).

15. M. D. Suttle, A. J. King, P. F. Schofield, H. Bates, S. S. Russell, The aqueous alteration of CM chondrites, a review. *Geochim. Cosmochim. Acta* **299**, 219–256 (2021).

16. M. Rüfenacht, P. Morino, Y.-J. Lai, M. A. Fehr, M. K. Haba, M. Schönbächler, Genetic relationships of solar system bodies based on their nucleosynthetic Ti isotope compositions and sub-structures of the solar protoplanetary disk. *Geochim. Cosmochim. Acta* **355**, 110–125 (2023).

17. T. Hiroi, H. Kaiden, N. Imae, K. Misawa, H. Kojima, S. Sasaki, M. Matsuoka, T. Nakamura, D. L. Bish, K. Ohtsuka, K. T. Howard, K. R. Robertson, R. E. Milliken, UV-visible-infrared spectral survey of Antarctic carbonaceous chondrite chips. *Polar Sci.* **29**, 100723 (2021).

18. M. E. Zolensky, A. Takenouchi, T. Mikouchi, T. Gregory, K. Nishiizumi, M. W. Caffee, M. A. Velbel, D. K. Ross, A. Zolensky, L. Le, N. Imae, A. Yamaguchi, The nature of the CM parent asteroid regolith based on cosmic ray exposure ages. *Meteorit. Planet. Sci.* **56**, 49–55 (2021).

19. D. Krietsch, H. Busemann, M. E. I. Riebe, A. J. King, C. M.O'D. Alexander, C. Maden, Noble gases in CM carbonaceous chondrites: Effect of parent body aqueous and thermal alteration and cosmic ray exposure ages. *Geochim. Cosmochim. Acta* **310**, 240–280 (2021).

20. V. S. Heber, R. Wieler, H. Baur, C. Olinger, T. A. Friedmann, D. S. Burnett, Noble gas composition of the solar wind as collected by the Genesis mission. *Geochim. Cosmochim. Acta* **73**, 7414–7432 (2009).

21. B. P. Weiss, X.-N. Bai, R. R. Fu, History of the solar nebula from meteorite paleomagnetism. *Sci. Adv.* **7**, aba5967 (2021).

22. V. K. Pearson, M. A. Sephton, I. A. Franchi, J. M. Gibson, I. Gilmour, Carbon and nitrogen in carbonaceous chondrites: Elemental abundances and stable isotopic compositions. *Meteorit. Planet. Sci.* **41**, 1899–1918 (2006).

23. Y. Kebukawa, K. Okudaira, H. Yabuta, S. Hasegawa, M. Tabata, Y. Furukawa, M. Ito, A. Nakato, A. L. D. Kilcoyne, K. Kobayashi, S.-I. Yokobori, E. Imai, Y. Kawaguchi, H. Yano, A. Yamagishi, STXM-XANES analyses of Murchison meteorite samples captured by aerogel after hypervelocity impacts: A potential implication of organic matter degradation for micrometeoroid collection experiments. *Geochem. J.* **53**, 53–67 (2019).

24. G. Danger, A. Ruf, J. Maillard, J. Hertzog, V. Vinogradoff, P. Schmitt-Kopplin, C. Afonso, N. Carrasco, I. Schmitz-Afonso, L. Le Sergeant d'Hendecourt, L. Remusat, Unprecedented molecular diversity revealed in meteoritic insoluble organic matter: The Paris Meteorite's case. *Planet. Sci. J.* **1**, 55 (2020).

25. P. Schmitt-Kopplin, N. Hertkorn, M. Harir, F. Moritz, M. Lucio, L. Bonal, E. Quirico, Y. Takano, J. P. Dworkin, H. Naraoka, S. Tachibana, T. Nakamura, T. Noguchi, R. Okazaki, H. Yabuta, H. Yurimoto, K. Sakamoto, T. Yada, M. Nishimura, A. Nakato, A. Miyazaki, K. Yogata, M. Abe, T. Usui, M. Yoshikawa, T. Saiki, S. Tanaka, F. Terui, S. Nakazawa, T. Okada, S.-I. Watanabe, Y. Tsuda, Hayabusa2-initial-analysis SOM team, Soluble organic matter molecular atlas of Ryugu reveals cold hydrothermalism on C-type asteroid parent body. *Nat. Commun.* **14**, 6525 (2023).

26. M. Lecasble, L. Remusat, J.-C. Viennet, B. Laurent, S. Bernard, Polycyclic aromatic hydrocarbons in carbonaceous chondrites can be used as tracers of both pre-accretion and secondary processes. *Geochim. Cosmochim. Acta* **335**, 2432–2255 (2022).

27. K. Slavicinska, D. Duca, D. Egorov, T. Mitra, Y. Carpentier, C. Focsa, C. J. Bennett, C. Pirim, Link between polycyclic aromatic hydrocarbon size and aqueous alteration in carbonaceous chondrites revealed by laser mass spectrometry. *ACS Earth Space Chem.* **6**, 1413–1428 (2022).

28. D. P. Glavin, M. P. Callahan, J. P. Dworkin, J. E. Elsila, The effects of parent body processes on amino acids in carbonaceous chondrites. *Meteorit. Planet. Sci.* **45**, 1948–1972 (2010).

29. D. P. Glavin, J. E. Elsila, H. L. McLain, J. C. Aponte, E. T. Parker, J. P. Dworkin, D. H. Hill, H. C. Connolly, D. S. Lauretta, Extraterrestrial amino acids and L enantiomeric excesses in the CM2 carbonaceous chondrite Aguas Zarcas and Murchison. *Meteorit. Planet. Sci.* **56**, 148–173 (2021).

30. G. D. Cody, C. M. O. Alexander, D. I. Foustoukos, H. Busemann, S. Eckley, A. S. Burton, E. L. Berger, M. Nuevo, S. A. Sandford, D. P. Glavin, J. P. Dworkin, H. C. Connolly, D. S. Lauretta, The nature of insoluble organic matter in Sutter's Mill and Murchison carbonaceous chondrites: Testing the effect of X-ray computed tomography and exploring parent body organic molecular evolution. *Meteorit. Planet. Sci.* **59**, 3–22 (2024).

31. M. E. Zolensky, R. N. Clayton, T. Mayeda, J. Chokai, O. R. Norton, Carbonaceous chondrite clasts in the halite-bearing H5 chondrite Zag. *Meteorit. Planet. Sci.* **38**, abstract id.5216 (2003).

32. Y. Kebukawa, M. Ito, M. E. Zolensky, R. C. Greenwood, Z. Rahman, H. Suga, A. Nakato, Q. H. S. Chan, M. Fries, Y. Takeichi, Y. Takahashi, K. Mase, K. Kobayashi, A novel organic-rich meteoritic clast from the outer solar system. *Nat. Sci. Rep.* **9**, 3169 (2019).

33. M. E. Zolensky, D. W. Mittlefehldt, M. E. Lipschutz, M.-S. Wang, R. N. Clayton, T. Mayeda, M. M. Grady, C. Pillinger, D. Barber, CM chondrites exhibit the complete petrologic range from type 2 to 1. *Geochim. Cosmochim. Acta* **61**, 5099–5115 (1997).

34. L. Browning, H. Y. McSween Jr., M. E. Zolensky, On the origin of rim textures surrounding anhydrous silicate grains in CM carbonaceous chondrites. *Meteorit. Planet. Sci.* **35**, 1015–1023 (2000).

35. D. R. Frank, G. R. Huss, K. Nagashima, M. E. Zolensky, "Sub-zero alteration in an isotopically heavy brine preserved in a pristine H chondrite xenolith" in *Proceedings of the 51st Lunar and Planetary Science Conference, held March 16–20, 2020, The Woodlands, Texas* (LPI Contribution No. 2020), p. 2911.

36. P. Jenniskens, H. A. R. Devillepoix, Review of asteroid-meteor-meteorite type links. *Meteorit. Planet. Sci.* **60**, 928–973 (2025).

37. B. Harvison, M. de Prá, N. Pinilla-Alonso, V. Lorenzi, J. de León, D. Morate, J. Licandro, A. Arredondo, H. Campins, PRIMASS near-infrared study of the Erigone collisional family. *Icarus* **412**, 115973 (2024).

38. M. Brož, P. Vernazza, M. Marsset, R. P. Binzel, F. DeMeo, M. Birlan, F. Colas, S. Anghel, S. Bouley, C. Blanpain, J. Gattacceca, S. Jeanne, L. Jorda, J. Lecubin, A. Malgoyre, A. Steinhausser, J. Vaubaillon, B. Zanda, Source regions of carbonaceous meteorites and near-Earth objects. *Astron. Astrophys.* **689**, A183 (2024).

39. S. Fornasier, C. Lantz, D. Perna, H. Campins, M. A. Barucci, D. Nesvorný, Spectral variability on primitive asteroids of the Themis and Beagle families: Space weathering effects or parent body heterogeneity? *Icarus* **269**, 1–14 (2016).

40. M. Marsset, P. Vernazza, M. Birlan, F. DeMeo, R. P. Binzel, C. Dumas, J. Milli, M. Popescu, Compositional characterisation of the Themis family. *Astron. Astrophys.* **586**, A15 (2016).

41. D. Takir, J. P. Emery, Outer Main Belt asteroids: Identification and distribution of our 3-mm spectral groups. *Icarus* **219**, 641–654 (2012).

42. H. Campins, K. Hargrove, N. Pinilla-Alonso, E. S. Howell, M. S. Kelley, J. Licandro, T. Mothé-Diniz, Y. Fernández, J. Ziffer, Water ice and organics on the surface of the asteroid 24 Themis. *Nature* **464**, 1320–1321 (2010).

43. H. H. Hsieh, B. Novaković, K. J. Walsh, N. Schörghofer, Potential Themis-family asteroid contribution to the Jupiter-family comet population. *Astron. J.* **159**, 179 (2020).

44. D. Nesvorny, P. Jenniskens, H. F. Levison, W. F. Bottke, D. Vokrouhlicky, M. Gounelle, Carbonaceous micrometeorites. Implications for hot debris disks. *Astron. J.* **713**, 816–836 (2010).

45. M. Hankey, V. Perlerin, D. Meisel, The All-Sky-6 and the video meteor archive system of the AMS ltd. *Planet. Space Sci.* **190**, 105005 (2020).

46. P. Jenniskens, M. D. Fries, Q.-Z. Yin, M. Zolensky, A. N. Krot, S. A. Sandford, D. Sears, R. Beauford, D. S. Ebel, J. M. Friedrich, K. Nagashima, J. Wimpenny, A. Yamakawa, K.

Nishiizumi, Y. Hamajima, M. W. Caffee, K. C. Welten, M. Laubenstein, A. M. Davis, S. B. Simon, P. R. Heck, E. D. Young, I. E. Kohl, M. H. Thiemens, M. H. Nunn, T. Mikouchi, K. Hagiya, K. Ohsumi, T. A. Cahill, J. A. Lawton, D. Barnes, A. Steele, P. Rochette, K. L. Verosub, J. Gattacceca, G. Cooper, D. P. Glavin, A. S. Burton, J. P. Dworkin, J. E. Elsila, S. Pizzarello, R. Ogliore, P. Schmitt-Kopplin, M. Harir, N. Hertkorn, A. Verchovsky, M. Grady, K. Nagao, R. Okazaki, H. Takechi, T. Hiroi, K. Smith, E. A. Silber, P. G. Brown, J. Albers, D. Klotz, M. Hankey, R. Matson, J. A. Fries, R. J. Walker, I. Puchtel, C. T. A. Lee, M. E. Erdman, G. R. Eppich, S. Roeske, Z. Gabelica, M. Lerche, M. Nuevo, B. Girten, S. P. Worden, Radar–enabled recovery of the Sutter's Mill meteorite, a carbonaceous chondrite regolith breccia. *Science* **338**, 1583–1587 (2012).

47. A. J. King, L. Daly, J. Row, K. H. Joy, R. C. Greenwood, H. A. R. Devillepoix, M. D. Suttle, Q. H. S. Chan, S. S. Russell, H. C. Bates, J. F. J. Bryson, P. L. Clay, D. Vida, M. R. Lee, A. O'Brien, L. J. Hallis, N. R. Stephen, R. Tartèse, E. K. Sansom, M. C. Towner, M. Cupak, P. M. Shober, P. A. Bland, R. Findlay, I. A. Franchi, A. B. Verchovsky, F. A. J. Abernethy, M. M. Grady, C. J. Floyd, M. van Ginneken, J. Bridges, L. J. Hicks, R. H. Jones, J. T. Mitchell, M. J. Genge, L. Jenkins, P.-E. Martin, M. A. Sephton, J. S. Watson, T. Salge, K. A. Shirley, R. J. Curtis, T. J. Warren, N. E. Bowles, F. M. Stuart, L. G. D. Di Nicola, A. J. Boyce, K. M. M. Shaw, T. Elliott, R. C. J. Steele, P. Povinec, M. Laubenstein, D. Sanderson, A. Cresswell, A. J. T. Jull, I. Sýkora, S. Sridhar, R. J. Harrison, F. M. Willcocks, C. S. Harrison, D. Hallatt, P. J. Wozniakiewicz, M. J. Burchell, L. S. Alesbrook, A. Dignam, N. V. Almeida, C. L. Smith, B. Clark, E. R. Humpreys-Williams, P. F. Schofield, L. T. Cornwell, V. Spathis, G. H. Morgan, M. J. Perkins, R. Kacerek, P. Campbell-Burns, F. Colas, B. Zanda, P. Vernazza, S. Bouley, S. Jeane, M. Hankey, G. S. Collins, J. S. Young, C. Shaw, J. Horak, D. Jones, N. James, S. Bosley, A. Shuttleworth, P. Dickinson, I. McMullan, D. Robson, A. R. D. Smedley, B. Stanley, R. Bassom, M. McIntyre, A. A. Suttle, R. Fleet, L. Bastiaens, M. B. Ihász, S. McMullan, S. J. Boazman, Z. I. Dickeson, P. M. Grindrod, A. E. Pickersgil, C. J. Weir, F. M. Suttle, S. Farrelly, I. Spencer, S. Naqvi, B. Mayne, D. Skilton, D. Kirk, A. Mounsey, S. E. Mounsey, S. Mounsey, P. Godfrey, L. Bond, V. Bond, C. Wilcock, H. Wilcock, R. Wilcock, The Winchcombe meteorite, a unique and pristine witness from the outer solar system. *Sci. Adv.* **8**, eabq3925 (2022).

48. R. A. Ketcham, Computational methods for quantitative analysis of three-dimensional features in geological specimens. *Geosphere* **1**, 32–41 (2005).

49. A. Cardona, S. Saalfeld, J. Schindelin, I. Arganda-Carreras, S. Preibisch, M. Longair, P. Tomancak, V. Hartenstein, R. J. Douglas, TrakEM2 software for neural circuit reconstruction. *PLOS ONE* **7**, e38011 (2012).

50. J. M. Friedrich, M. M. Chen, S. A. Giordano, O. K. Matalka, J. W. Strasser, K. A. Tamucci, M. L. Rivers, D. S. Ebel, Size-frequency distributions and physical properties of chondrules from x-ray computed microtomography and digital data extraction. *Microsc. Res. Tech.* **85**, 1814–1824 (2022).

51. T. Zingg, Beitrag zur Schotteranalyse: Die Schotteranalyse und ihre Anwendung auf die Glattalschotter. *Schweizerische Mineralogische Und Petrographische Mitteilungen* **15**, 39–140.41 (1935).

52. S. J. Blott, K. Pye, Particle Shape: A review and new methods of characterization and classification. *Sedimentology* **55**, 31–63 (2008).

53. D. L. Schrader, Z. A. Torrano, D. I. Foustoukos, C. M. O'D. Alexander, J. Render, G. A. Brennecka, Reassessing the proposed "CY chondrites": Evidence for multiple meteorite types and parent bodies from Cr-Ti-H-C-N isotopes and bulk elemental compositions. *Geochim. Cosmochim. Acta* **390**, 24–37 (2025).

54. T. Hiroi, K. Ohtsuka, M. E. Zolensky, M. J. Rutherford, R. E. Milliken, "Tagish Lake is still the only possible meteorite sample from D-type asteroids" in *Proceedings of the 53th Lunar and Planetary Science Conference, held 7–11 March, 2022 at The Woodlands, Texas* (LPI 2020), p. 1149.

55. K. Nishiizumi, M. W. Caffee, Y. Hamajima, R. C. Reedy, K. C. Welten, Exposure history of the Sutter's Mill carbonaceous chondrite. *Meteorit. Planet. Sci.* **49**, 2056–2063 (2014).

56. P. Sharma, M. Bourgeois, D. Elmore, D. Granger, M. E. Lipschutz, X. Ma, T. Miller, K. Mueller, F. Rickey, P. Simms, S. Vogt, PRIME lab AMS performance, upgrades and research applications. *Nucl. Instrum. Methods Phys. Res.* **172**, 112–123 (2000).

57. H. Busemann, H. Baur, R. Wieler, Primordial noble gases in “Phase Q” in carbonaceous and ordinary chondrites studied by closed system stepped etching. *Meteorit. Planet. Sci.* **35**, 949–973 (2000).

58. M. E. I. Riebe, K. C. Welten, M. M. M. Meier, R. Wieler, M. I. F. Barth, D. Ward, A. Bischoff, M. W. Caffee, K. Nishiizumi, H. Busemann, Cosmic-ray exposure ages of six chondritic Almahata Sitta fragments. *Meteorit. Planet. Sci.* **52**, 2353–2374 (2017).

59. J. L. Kirschvink, R. E. Kopp, T. D. Raub, C. T. Baumgartner, J. W. Holt, Rapid, precise, and high-sensitivity acquisition of paleomagnetic and rock-magnetic data: Development of a low-noise automatic sample changing system for superconducting rock magnetometers. *Geochem. Geophys. Geosyst.* **9**, 1–18 (2008).

60. N. O. Ogawa, Y. Kebukawa, M. Zolensky, Y. Takano, N. Ohkouchi, “Isotopic measurements of <100 ng carbon and nitrogen through EA/IRMS and its application to extraterrestrial materials” in *Proceedings of the 82nd Annual Meeting of the Meteoritical Society, held 7–12 July 2019 in Sapporo, Japan* (LPI 2019), p. 6208.

61. N. O. Ogawa, T. Nagata, H. Kitazato, N. Ohkouchi, Ultra-sensitive elemental analyzer/isotope ratio mass spectrometer for stable nitrogen and carbon isotope analyses in *Earth, Life, and Isotopes*, N. Ohkouchi, I. Tayasu, K. Koba Eds. (Kyoto Univ. Press 2010), pp. 339–353.

62. Y. Isaji, N. O. Ogawa, C. J. Boreham, Y. Kashiyama, N. Ohkouchi, Evaluation of $\delta^{13}C$ and $\delta^{15}N$ uncertainties associated with the compound-specific isotope analysis of geoporphyrins. *Anal. Chem.* **92**, 3152–3160 (2020).

63. A. Bischoff, C. M. O’D. Alexander, J.-A. Barrat, C. Burkhardt, H. Busemann, D. Degering, T. Di Rocco, M. Fischer, T. Fockenberg, D. I. Foustoukos, J. Gattacceca, J. R. A. Godinho, D. Harries, D. Heinlein, J. L. Hellmann, N. Hertkorn, A. Holm, A. J. T. Jull, I. Kerraouch, A. J. King, T. Kleine, D. Koll, J. Lachner, T. Ludwig, S. Merchel, C. A. K. Mertens, P. Morino, W.

Neumann, A. Pack, M. Patzek, S. Pavetich, M. P. Reitze, M. Rüfenacht, G. Rugel, C. Schmidt, P. Schmitt-Kopplin, M. Schönbächler, M. Trieloff, A. Wallner, K. Wimmer, E. Wölfer, The old, unique C1 chondrite Flensburg – Insight into the first processes of aqueous alteration, brecciation, and the diversity of water-bearing parent bodies and lithologies. *Geochim. Cosmochim. Acta* **293**, 142–186 (2021).

64. Y. Kebukawa, E. Quirico, E. Dartois, H. Yabuta, L. Bejach, L. Bonal, A. Dazzi, A. Deniset-Besseau, J. Duprat, C. Engrand, J. Mathurin, J. Barosch, G. D. Cody, B. De Gregorio, M. Hashiguchi, K. Kamide, D. Kilcoyne, M. Komatsu, Z. Martins, G. Montagnac, S. Mostefaoui, L. R. Nittler, T. Ohigashi, T. Okumura, L. Remusat, S. Sandford, M. Shigenaka, R. Stroud, H. Suga, Y. Takahashi, Y. Takeichi, Y. Tamenori, M. Verdier-Paoletti, D. Wakabayashi, S. Yamashita, H. Yurimoto, T. Nakamura, T. Noguchi, R. Okazaki, H. Naraoka, K. Sakamoto, S. Tachibana, T. Yada, M. Nishimura, A. Nakato, A. Miyazaki, K. Yogata, M. Abe, T. Okada, T. Usui, M. Yoshikawa, T. Saiki, S. Tanaka, F. Terui, S. Nakazawa, S.-I. Watanabe, Y. Tsuda, Infrared absorption spectra from organic matter in the asteroid Ryugu samples: Some unique properties compared to unheated carbonaceous chondrites. *Meteorit. Planet. Sci.* **59**, 1845–1858 (2024).

65. Y. Takeichi, N. Inami, H. Suga, C. Miyamoto, T. Ueno, K. Mase, Y. Takahashi, K. Ono, Design and performance of a compact scanning transmission x-ray microscope at the Photon Factory. *Rev. Sci. Instrum.* **87**, 013704 (2016).

66. Y. Kebukawa, J. Mathurin, E. Dartois, A. Dazzi, A. Deniset-Besseau, J. Duprat, L. Rémusat, T. Noguchi, A. Miyake, Y. Igami, M. Verdier Paoletti, M. E. Zolensky, C. Engrand, C. Sandt, F. Borondics, S. Yamashita, D. Wakabayashi, Y. Takeichi, Y. Takahashi, Complex mixture of organic matter in a xenolithic clast from the Zag meteorite revealed by coordinated analyses using AFM-IR, NanoSIMS and STXM/XANES. *Icarus* **400**, 115582 (2023).

67. Z. Gainsforth, G. Dominguez, K. Amano, M. Matsumoto, Y. Fujioka, E. Kagawa, T. Nakamura, S. Tachibana, T. Morita, M. Kikuiri, H. Yurimoto, T. Noguchi, R. Okazaki, H. Yabuta, H. Naraoka, K. Sakamoto, T. Yada, M. Nishimura, A. Nakato, A. Miyazaki, K. Yogata, M. Abe, T. Okada, T. Usui, M. Yoshikawa, T. Saiki, S. Tanaka, F. Terui, S. Nakazawa, S.-I. Watanabe, Y. Tsuda, Hayabusa2 Initial Analysis Stone Team, Coevolution of

phyllosilicate, carbon, sulfide, and apatite in Ryugu's parent body. *Meteorit. Planet. Sci.* **59**, 2073–2096 (2024).

68. P. Schmitt-Kopplin, Z. Gabelica, R. D. Gougeon, A. Fekete, B. Kanawati, M. Harir, I. Gebefuegi, G. Eckel, N. Hertkorn, High molecular diversity of extraterrestrial organic matter in Murchison meteorite revealed 40 years after its fall. *Proc. Natl. Acad. Sci. U.S.A.* **107**, 2763–2768 (2010).

69. J. C. Aponte, H. L. McLain, D. N. Simkus, J. E. Elsila, D. P. Glavin, E. T. Parker, J. P. Dworkin, D. H. Hill, H. C. Connolly Jr., D. S. Lauretta, Extraterrestrial organic compounds and cyanide in the CM2 carbonaceous chondrites Aguas Zarcas and Murchison. *Meteorit. Planet. Sci.* **55**, 1509–1524 (2020).

70. J. C. Aponte, R. Tarozo, M. R. Alexandre, C. M. D. Alexander, S. B. Charnley, C. Hallmann, R. E. Summons, Y. Huang, Chirality of meteoritic free and IOM-derived monocarboxylic acids and implications for prebiotic organic synthesis. *Geochim. Cosmochim. Acta* **131**, 1–12 (2014).

71. J. Borovička, O. Popova, P. Spurný, The Maribo CM2 meteorite fall – Survival of weak material at high entry speed. *Meteorit. Planet. Sci.* **54**, 1024–1041 (2019).

72. M. Fries, J. Fries, Weather radar detection and analysis of bolides in the day or night. *Adv. Astron* **2025**, 3041760 (2025).

73. M. R. Lee, T. Salge, I. Maclaren, Magnesium phosphate in the Cold Bokkeveld (CM2) carbonaceous chondrite. *Meteorit. Planet. Sci.* **60**, 2017–2025 (2025).

74. S. Russell, M. Suttle, A. King, Abundance and importance of petrological type 1 chondritic material. *Meteorit. Planet. Sci.* **57**, 277–301 (2022).

75. M. Weisberg, H. Huber, The GRO 95577 CR1 chondrite and hydration of the CR parent body. *Meteorit. Planet. Sci.* **42**, 1495–1503 (2007).

76. J. M. Friedrich, E. M. Riveros, R. J. Macke, S. J. Jaret, M. L. Rivers, D. S. Ebel, Physical properties, internal structure, and the three-dimensional petrography of CI chondrites. *Meteorit. Planet. Sci.* **60**, 632–645 (2025).

77. C. M. O'D. Alexander, Quantitative models for the elemental and isotopic fractionations in chondrites: The carbonaceous chondrites. *Geochim. Cosmochim. Acta* **254**, 277–309 (2019).

78. I. Leya, J. Masarik, Cosmogenic nuclides in stony meteorites revisited. *Meteorit. Planet. Sci.* **44**, 1061–1086 (2009).

79. R. E. Kopp, B. P. Weiss, A. C. Maloof, H. Vali, C. Z. Nash, J. L. Kirschvink, Chains, clumps, and strings: Magnetofossil taphonomy with ferromagnetic resonance spectroscopy. *Earth Planet. Sci. Lett.* **247**, 10–25 (2006).

80. C. Maurel, J. Gattacceca, Estimating paleointensities from chemical remanent magnetizations of magnetite using non-heating methods. *J. Geophys. Res. Planets* **128**, e2023JE007779 (2023).

81. I. Tayasu, R. Hirasawa, N. O. Ogawa, N. Ohkouchi, K. Yamada, New organic reference materials for carbon- and nitrogen-stable isotope ratio measurements provided by Center for Ecological Research, Kyoto University, and Institute of Biogeosciences, Japan Agency for Marine-Earth Science and Technology. *Limnology* **12**, 261–266 (2011).

82. M. A. Sephton, A. B. Verchovsky, P. A. Bland, I. Gilmour, M. M. Grady, I. P. Wright, Investigating the variations in carbon and nitrogen isotopes in carbonaceous chondrites. *Geochim. Cosmochim. Acta* **67**, 2093–2108 (2003).

83. A. Ruf, B. Kanawati, N. Hertkorn, Q.-Z. Yin, F. Moritz, M. Harir, M. Lucio, B. Michalke, J. Wimpenny, S. Shilobreeva, B. Bronsky, V. Saraykin, Z. Gabelica, R. D. Gougeon, E. Quirico, S. Ralew, T. Jakubowski, H. Haack, M. Gonsior, P. Jenniskens, N. W. Hinman, P. Schmitt-Kopplin, Previously unknown class of metalorganic compounds revealed in meteorites. *Proc. Natl. Acad. Sci. U.S.A.* **114**, 2819–2824 (2017).

84. B. P. Koch, T. Dittmar, From mass to structure: An aromaticity index for high-resolution mass data of natural organic matter. *Rapid Commun. Mass Spectrom.* **30**, 250–250 (2016).

85. J. C. Aponte, H. K. Woodward, N. M. Abreu, J. E. Elsila, J. P. Dworkin, Molecular distribution, 13C-isotope, and enantiomeric compositions of carbonaceous chondrite monocarboxylic acids. *Meteorit. Planet. Sci.* **54**, 415–430 (2019).

86. F. Robert, S. Epstein, The concentration and isotopic composition of hydrogen, carbon and nitrogen in carbonaceous meteorites. *Geochim. Cosmochim. Acta* **46**, 81–95 (1982).

87. D. P. Glavin, J. P. Dworkin, C. M. O'D. Alexander, J. C. Aponte, A. A. Baczynski, J. J. Barnes, H. A. Bechtel, E. L. Berger, A. S. Burton, P. Caselli, A. H. Chung, S. J. Clemett, G. D. Cody, G. Dominguez, J. E. Elsila, K. K. Farnsworth, D. I. Foustoukos, K. H. Freeman, Y. Furukawa, Z. Gainsforth, H. V. Graham, T. Grassi, B. M. Giuliano, V. E. Hamilton, P. Haenecour, P. R. Heck, A. E. Hofmann, C. H. House, Y. Huang, H. H. Kaplan, L. P. Keller, B. Kim, T. Koga, M. Liss, H. L. McLain, M. A. Marcus, M. Matney, T. J. McCoy, O. M. McIntosh, A. Mojarro, H. Naraoka, A. N. Nguyen, M. Nuevo, J. A. Nuth III, Y. Oba, E. T. Parker, T. S. Peretyazhko, S. A. Sandford, E. Santos, P. Schmitt-Kopplin, F. Seguin, D. N. Simkus, A. Shahid, Y. Takano, K. L. Thomas-Keprta, H. Tripathi, G. Weiss, Y. Zheng, N. G. Lunning, K. Righter, H. C. Connolly Jr., D. S. Lauretta, Abundant ammonia and nitrogen-rich soluble organic matter in samples from asteroid (101955) Bennu. *Nat. Astron* **9**, 199–210 (2025).
